# Natural Products as Nutritional Supplements in Human Disease Prevention and Management: From Molecular Mechanisms to Clinical Translation

**DOI:** 10.3390/nu18142362

**Published:** 2026-07-18

**Authors:** Antonios Dakanalis, Sousana K. Papadopoulou, Maria Mentzelou, Athanasios Migdanis, Ioannis Migdanis, Constantinos Giaginis

**Affiliations:** 1 Department of Mental Health, Fondazione IRCSS San Gerardo dei Tintori, 20900 Monza, Italy; 2 Department of Medicine and Surgery, University of Milano Bicocca, Via Cadore 38, 20900 Monza, Italy; 3 Department of Nutritional Sciences and Dietetics, School of Health Sciences, International Hellenic University, 57400 Thessaloniki, Greece; souzpapa@gmail.com; 4 Department of Food Science and Nutrition, School of Environment, University of Aegean, 81400 Myrina, Lemnos, Greece; maria.mentzelou@hotmail.com; 5 Nutrition and Dietetics Department, University of Thessaly, 42132 Trikala, Greece; amigdanis@gmail.com; 6 Faculty of Medicine, University of Thessaly, 41110 Larissa, Greece; imigdanis@uth.gr

**Keywords:** natural products, nutritional supplements, human diseases, antioxidants, anti-inflammatory, bioavailability, preclinical evidence, clinical trials

## Abstract

Background/Objectives: Natural products derived from plants, animals, and microorganisms have long been used as nutritional supplements and are increasingly recognized for their potential role in preventing and managing human diseases. This narrative review aims to summarize current evidence on the therapeutic relevance of natural products as dietary supplements across major disease categories and to highlight their mechanisms of action, clinical efficacy, and safety considerations. Methods: A narrative literature review was conducted using peer-reviewed articles, systematic reviews, and clinical studies focusing on natural products used as nutritional supplements in disease management. Relevant data were analyzed thematically, with emphasis on bioactive compounds, mechanisms of action, and evidence from preclinical and clinical research. Results: Natural products, particularly plant-derived polyphenols, flavonoids, terpenoids, omega-3 fatty acids, and probiotic-derived metabolites, exhibit diverse biological activities, including antioxidant, anti-inflammatory, immunomodulatory, and antimicrobial effects. Evidence suggests potential benefits in cardiovascular diseases, metabolic disorders such as diabetes and obesity, neurodegenerative conditions, certain cancers, gastrointestinal disorders, and infectious diseases. However, clinical efficacy varies depending on compound type, dosage, and formulation. Key limitations include low bioavailability, variability in composition, and insufficient large-scale clinical trials. Safety concerns such as herb–drug interactions and lack of standardization remain significant challenges. Conclusions: Natural products as nutritional supplements represent a promising adjunct strategy in the prevention and management of various human diseases. While preclinical and early clinical evidence is encouraging, stronger clinical validation, improved standardization, and clearer regulatory frameworks are required to fully integrate these agents into evidence-based medical practice.

## 1. Introduction

Natural products have played a pivotal role in human health throughout history, serving as the foundation of traditional medical systems such as Ayurveda, Traditional Chinese Medicine, Traditional Korean Medicine, Traditional Japanese Medicine (Kampo), Unani medicine, and diverse indigenous and healing practices worldwide. Historical evidence from ancient Egyptian, Greek, Chinese, Indian, Korean, Japanese, and other traditional medical systems indicates that plant-derived preparations, fermented products, and animal-derived substances were extensively employed for disease prevention, treatment, and the promotion of health and longevity [1,2,3]. Advances in modern pharmacology and natural product chemistry have increasingly validated many of these traditional applications, leading to the identification of numerous bioactive compounds with significant therapeutic potential [1,2]. Indeed, a substantial proportion of contemporary pharmaceuticals are either directly derived from natural sources or inspired by natural-product scaffolds, underscoring their enduring importance in drug discovery and preventive medicine [3].

Natural products used as nutritional supplements comprise a diverse group of bioactive compounds derived from plant, animal, and microbial sources that provide physiological benefits beyond basic nutritional requirements. These include plant-derived phytochemicals such as polyphenols, flavonoids, terpenoids, and alkaloids; animal-derived bioactive compounds such as omega-3 fatty acids; and microbiome-related interventions including probiotics, prebiotics, and microbial metabolites [3,4]. Increasing evidence indicates that these compounds exert pleiotropic biological effects through modulation of multiple molecular and cellular pathways. For example, dietary polyphenols possess antioxidant, anti-inflammatory, antimicrobial, and cardiometabolic activities and have been implicated in the prevention of numerous chronic diseases [5,6]. Importantly, contemporary research has demonstrated that many natural compounds function not merely as direct antioxidants but also as regulators of intracellular signaling networks, gene expression, redox homeostasis, and immune responses [7,8].

Interest in natural products as preventive and therapeutic interventions has intensified in response to the growing global burden of non-communicable diseases, including cardiovascular disease (CVD), obesity, type 2 diabetes, neurodegenerative disorders, and cancer [9,10]. Epidemiological and clinical studies consistently suggest that dietary patterns rich in bioactive phytochemicals are associated with improved metabolic health and reduced disease risk [9,10]. Concurrently, advances in molecular biology, systems biology, and omics technologies have substantially improved understanding of the mechanisms underlying these effects, revealing the capacity of natural compounds to modulate key biological processes such as inflammation, oxidative stress, immune regulation, apoptosis, and cellular signaling pathways, including nuclear factor-κB (NF-κB) and mitogen-activated protein kinase (MAPK) signaling [11,12,13]. Furthermore, increasing consumer demand for natural and preventive healthcare approaches has contributed to the rapid expansion of the global nutraceutical market [6,9]. Nevertheless, despite extensive preclinical evidence, clinical outcomes remain variable and often inconsistent, highlighting the challenges associated with translating mechanistic findings into reproducible therapeutic benefits.

Despite the rapidly expanding literature, current knowledge remains fragmented. Many reviews focus on individual classes of natural compounds or specific disease categories, limiting a comprehensive understanding of their broader therapeutic potential. Moreover, mechanistic and preclinical findings are frequently emphasized without sufficient integration of clinical evidence or consideration of important translational challenges, including bioavailability, pharmacokinetic variability, formulation-dependent efficacy, safety, and regulatory standardization [6,8]. Emerging evidence further indicates that interactions between natural products and the gut microbiota substantially influence metabolism, bioavailability, and biological activity, adding another layer of complexity to their clinical application and contributing to interindividual variability in treatment responses [11,14].

Importantly, although the clinical manifestations of cardiovascular, metabolic, neurodegenerative, oncological, gastrointestinal, and infectious diseases differ substantially, these conditions share several underlying pathological processes, including chronic inflammation, oxidative stress, immune dysregulation, metabolic dysfunction, and alterations in host–microbiome interactions. Consequently, examining natural products across diverse disease domains provides an opportunity to identify common mechanistic pathways and translational principles that may not be apparent in disease-specific reviews.

Accordingly, there is a need for a comprehensive and integrative evaluation of natural products that bridges mechanistic insights, preclinical evidence, and clinical outcomes across multiple disease domains. The present narrative review aims to critically examine the role of natural products as nutritional supplements in the prevention and management of human diseases. Specifically, it synthesizes current evidence regarding major classes of bioactive compounds, their mechanisms of action, and their clinical relevance in cardiovascular, metabolic, neurodegenerative, oncological, gastrointestinal, and infectious diseases. In addition, the review addresses key translational challenges, including bioavailability, standardization, safety, regulatory considerations, and interindividual variability. By integrating evidence across molecular, experimental, and clinical levels, this review seeks to provide a comprehensive and clinically relevant perspective on the opportunities and limitations of natural products in contemporary healthcare, while identifying critical research priorities for their evidence-based implementation. Unlike previous reviews that focus on individual compound classes or specific disease categories, the present review adopts an integrative translational framework that examines natural products across diverse disease domains, while emphasizing common molecular mechanisms, clinical evidence, and translational challenges.

## 2. Methods

### 2.1. Study Design

This study was conducted as a comprehensive narrative review designed to provide an interdisciplinary and translational synthesis of the current evidence regarding the role of natural products, nutraceuticals, and microbiome-derived interventions in the prevention and management of human diseases. Narrative reviews are particularly suited for integrating heterogeneous forms of evidence originating from diverse scientific disciplines, including molecular biology, nutritional sciences, pharmacology, microbiology, epidemiology, and clinical medicine. Such an approach facilitates the contextual interpretation of complex findings, identification of knowledge gaps, and formulation of future research priorities that may not be adequately captured through conventional systematic review methodologies [15,16].

The review encompassed major classes of bioactive natural products, including plant-derived phytochemicals, animal-derived bioactive compounds, probiotics, prebiotics, postbiotics, and microbiome-derived metabolites. Particular emphasis was placed on their molecular mechanisms of action, biological targets, therapeutic applications, translational relevance, safety profiles, and clinical efficacy across major disease domains, including cardiovascular diseases, metabolic disorders, neurodegenerative diseases, cancer, gastrointestinal disorders, and infectious diseases. A narrative review methodology was selected because the objective extended beyond the evaluation of a single intervention–outcome relationship and instead sought to integrate evidence across multiple compound classes, mechanistic pathways, experimental models, and clinical contexts. This approach enables the synthesis of emerging concepts and multifaceted evidence that may not be amenable to formal quantitative meta-analysis.

Overall, given the breadth and complexity of the topic, which encompasses multiple classes of natural products, diverse disease categories, and heterogeneous study designs, a narrative review methodology was deemed the most appropriate approach for synthesizing and critically interpreting the available evidence.

### 2.2. Literature Search Strategy

A structured and comprehensive literature search was performed using four major electronic databases: PubMed, Scopus, Web of Science, and Google Scholar. The search primarily covered publications published between January 2015 and June 2025 to capture recent advances in natural product research, nutraceutical development, functional foods, microbiome science, and precision nutrition. Foundational studies, seminal publications, and highly cited landmark articles published prior to this period were also included when necessary to provide historical context and support key scientific concepts.

Search strategies incorporated both controlled vocabulary terms and free-text keywords related to natural products, nutraceuticals, dietary bioactive compounds, microbiome-derived metabolites, and disease-specific outcomes. Representative search terms included “natural products,” “nutraceuticals,” “functional foods,” “dietary supplements,” “polyphenols,” “flavonoids,” “phenolic acids,” “stilbenes,” “alkaloids,” “terpenoids,” “omega-3 fatty acids,” “probiotics,” “prebiotics,” “postbiotics,” “short-chain fatty acids,” “gut microbiota,” “oxidative stress,” “inflammation,” “epigenetics,” “cardiovascular disease,” “metabolic syndrome,” “type 2 diabetes,” “obesity,” “neurodegenerative disease,” “Alzheimer’s disease,” “Parkinson’s disease,” “cancer,” “infectious diseases,” “immune modulation,” and “chronic disease prevention.” Boolean operators (AND, OR, NOT), phrase searching, truncation functions, and database-specific filters were applied, where appropriate, to optimize search sensitivity and specificity. Literature searches were updated periodically throughout manuscript preparation to ensure inclusion of newly published evidence.

To enhance comprehensiveness and minimize publication omission bias, the reference lists of eligible articles, systematic reviews, meta-analyses, consensus statements, and authoritative reports were manually screened to identify additional relevant studies not captured through electronic database searches. This supplementary snowball-searching strategy is widely recommended for narrative evidence syntheses and integrative reviews [17].

### 2.3. Eligibility Criteria

Publications were selected according to predefined eligibility criteria based on their scientific relevance and contribution to the objectives of the review. Eligible sources included peer-reviewed original research articles, randomized controlled trials (RCTs), non-randomized clinical studies, prospective and retrospective observational studies, cohort studies, case–control studies, cross-sectional investigations, systematic reviews, meta-analyses, mechanistic in vitro studies, animal experiments, consensus statements, and professional guidelines directly related to natural products and nutraceutical interventions.

Studies were included if they investigated natural products, nutraceuticals, functional foods, dietary bioactive compounds, probiotics, prebiotics, postbiotics, or microbiome-derived metabolites and reported mechanistic, physiological, clinical, epidemiological, or therapeutic outcomes relevant to disease prevention, health promotion, or disease management. Studies focusing exclusively on synthetic pharmaceutical agents without direct relevance to natural products were excluded. Additional exclusion criteria comprised conference abstracts lacking sufficient methodological detail, editorials, commentaries, opinion articles without supporting primary evidence, duplicate publications, retracted studies, reports with inadequate methodological transparency, and publications not available in English.

To strengthen the clinical relevance of the synthesis, priority was given to higher levels of evidence, particularly systematic reviews, meta-analyses, randomized controlled trials, and large prospective cohort studies. Mechanistic and preclinical studies were incorporated primarily to support biological plausibility, elucidate mechanisms of action, and provide translational context.

### 2.4. Data Extraction and Evidence Organization

Relevant information was extracted manually using a standardized thematic framework developed specifically for the purposes of this review. Extracted variables included the category and source of the natural product, principal bioactive constituents, molecular targets, signaling pathways, proposed mechanisms of action, study design, experimental model, population characteristics, disease area investigated, intervention characteristics (including dosage, formulation, and treatment duration), primary outcomes, key findings, and reported safety considerations.

To facilitate interdisciplinary integration and comparative analysis, evidence was organized according to compound class, biological mechanism, and disease category. Compound classes included polyphenols, alkaloids, terpenoids, omega-3 fatty acids, probiotics, and microbiome-derived metabolites. Mechanistic categories included antioxidant activity, anti-inflammatory signaling, immune modulation, epigenetic regulation, metabolic reprogramming, and gut microbiota interactions. Disease domains included cardiovascular diseases, metabolic disorders, neurodegenerative diseases, cancer, gastrointestinal disorders, and infectious diseases.

This thematic framework enabled systematic integration and comparison of mechanistic, preclinical, clinical, and population-level evidence while maintaining conceptual coherence across diverse scientific disciplines. Afterwards, final study selection, data extraction, and evidence verification were also independently performed by the two reviewers (A.D. and C.G.), with discrepancies also resolved through discussion and consensus. Titles, abstracts, and full texts were then screened independently by the two reviewers (A.D. and C.G.), and disagreements were resolved through discussion and consensus. When multiple studies addressed similar topics, greater emphasis was placed on systematic reviews, meta-analyses, randomized controlled trials, and large prospective cohort studies due to their higher level of evidence.

**Figure 1:**
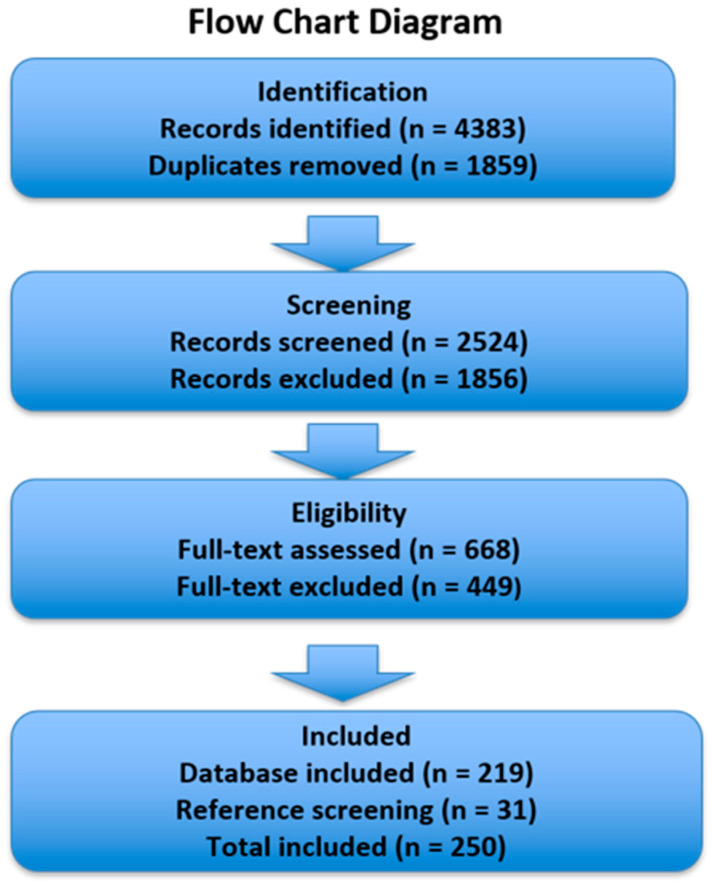
Flow diagram illustrating the literature search, screening, eligibility assessment, and study selection process for inclusion in the narrative review.

The initial literature search identified 4383 records across all databases. Following removal of duplicate entries (n = 1859), 2524 records remained for title and abstract screening. Records were excluded during title/abstract screening due to the following reasons: (i) not related to natural products/nutraceuticals, (ii) not focused on disease prevention or management, (iii) non-human/non-relevant experimental context, and (iv) conference abstracts/editorials/commentaries. After exclusion of the above non-relevant publications (n = 1859), 668 full-text articles were assessed for eligibility. After thoroughly reading the 668 full-text articles, 449 were excluded due to the following reasons: (i) insufficient relevance to review objectives, (ii) no specific natural product intervention evaluated, (iii) inadequate methodological information, (iv) not available in English, and (v) full text unavailable. Finally, 219 studies met the predefined inclusion criteria through database screening. An additional 31 studies were identified through manual reference-list searches, resulting in a final evidence base comprising 250 studies included in the qualitative synthesis. A flow diagram summarizing the study selection process is presented in Figure 1.

### 2.5. Evidence Synthesis

A qualitative narrative synthesis was undertaken to integrate and critically evaluate evidence across mechanistic, preclinical, clinical, and epidemiological domains. The included literature was systematically organized according to major disease categories and underlying biological mechanisms to identify recurring patterns, areas of consensus, conflicting findings, translational challenges, and emerging research priorities.

To enhance the scientific rigor of the synthesis, greater evidentiary weight was assigned to systematic reviews, meta-analyses, RCTs, and large prospective cohort studies, as these study designs generally provide the most robust evidence regarding clinical efficacy, safety, and translational relevance. Mechanistic in vitro studies and animal experiments were incorporated primarily to establish biological plausibility, elucidate molecular and cellular mechanisms of action, and provide a translational framework for interpreting clinical observations.

Particular consideration was given to the consistency and reproducibility of findings across studies, the magnitude and clinical relevance of reported effects, dose–response relationships, formulation-dependent bioavailability, safety profiles, and the overall strength of evidence supporting therapeutic applications. Given the substantial heterogeneity among studies with respect to populations, interventions, formulations, outcome measures, and methodological approaches, quantitative pooling of results was not considered appropriate. Accordingly, evidence was synthesized narratively to provide a balanced and context-specific interpretation of the current literature, consistent with established methodological frameworks for narrative and integrative reviews in nutritional and nutraceutical research [18].

Although a formal risk-of-bias assessment was not performed due to the narrative nature of the review, the strength of evidence was considered during data interpretation. Greater emphasis was placed on findings supported by systematic reviews, meta-analyses, randomized controlled trials, and large prospective studies, whereas evidence derived from preclinical and mechanistic investigations was interpreted primarily as supportive of biological plausibility rather than definitive clinical efficacy.

Particular attention was given to the translational relevance of the included studies, including the magnitude and reproducibility of reported clinical effects, consistency across populations, potential sources of heterogeneity, and discrepancies between preclinical and clinical findings.

### 2.6. Methodological Considerations and Limitations

Several methodological considerations should be acknowledged when interpreting the findings of this review. First, although a structured and transparent narrative review methodology was employed, the study was not conducted as a formal systematic review. While comprehensive database searches, predefined eligibility criteria, and independent study selection by two reviewers were implemented to enhance methodological rigor, the review did not adhere to PRISMA reporting guidelines and did not include formal risk-of-bias assessment, GRADE evaluation, or quantitative evidence grading procedures [17,18]. Consequently, the potential influence of publication bias, selection bias, and subjective interpretation cannot be entirely excluded.

Second, the evidence base was characterized by substantial heterogeneity in study design, participant characteristics, intervention protocols, product formulations, bioavailability profiles, analytical methodologies, and outcome measures. Such variability limits direct comparability among studies and complicates assessment of the overall strength and consistency of the evidence. Additional challenges arise from the inherent complexity of natural products, including variability in botanical sources, extraction methods, manufacturing processes, compositional standardization, dosing regimens, and reporting practices. These factors may significantly influence biological activity, reproducibility, and translational applicability across studies.

Furthermore, many natural-product interventions continue to be supported predominantly by mechanistic and preclinical evidence, whereas high-quality clinical trials remain limited for several compound classes and disease indications. Consequently, caution is warranted when extrapolating experimental findings to clinical settings.

To minimize selection bias, literature searches were conducted across multiple databases, predefined eligibility criteria were applied, reference lists were manually screened, and study selection and evidence verification were independently performed by the two reviewers (A.D. and C.G.). Nevertheless, the absence of formal quantitative evidence grading and risk-of-bias assessment should be considered when interpreting the conclusions of this narrative synthesis.

Despite these limitations, every effort was made to ensure comprehensive coverage of recent, influential, and methodologically robust literature. By integrating evidence from molecular, experimental, clinical, and population-based research, this review provides a multidisciplinary overview of the current state of knowledge regarding natural products and nutraceutical interventions. Moreover, it highlights critical gaps in the literature and identifies priorities for future research, product standardization, regulatory harmonization, clinical translation, and evidence-based implementation in disease prevention and management. Finally, efforts were made to enhance transparency and reproducibility through predefined eligibility criteria, structured literature searches, independent study selection, and standardized evidence organization.

## 3. Classification of Natural Products Used as Nutritional Supplements

**Figure 2:**
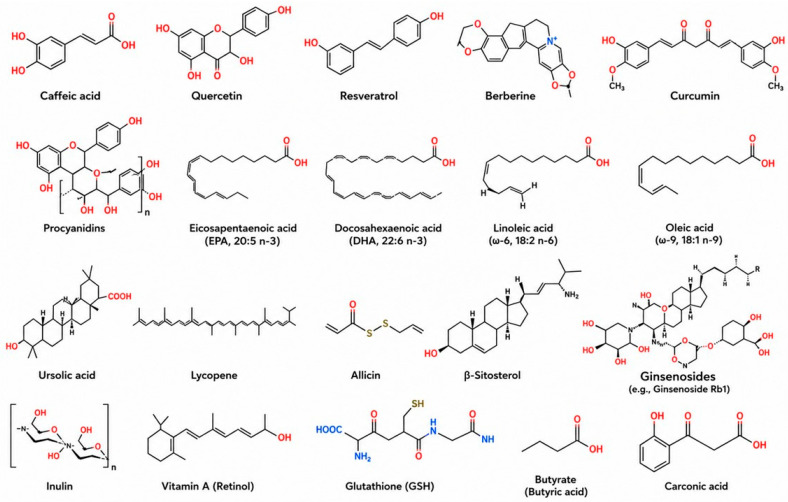
Chemical structures of representative bioactive compounds of the major classes of natural products: phenolic acids (caffeic acid), flavonoids (quercetin, procyanidins), stilbenes (resveratrol), alkaloids (berberine), curcuminoids (curcumin), omega fatty acids (eicosapentaenoic acid [EPA], docosahexaenoic acid [DHA], linoleic acid, oleic acid), terpenoids and carotenoids (ursolic acid, lycopene, vitamin A [retinol], carnosic acid), organosulfur compounds (allicin), phytosterols (β-sitosterol), saponins (ginsenosides), polysaccharides and prebiotics (inulin), endogenous antioxidants (glutathione), and microbiome-derived metabolites (postbiotics) (butyrate).

Natural products used as nutritional supplements comprise a structurally and functionally diverse group of bioactive compounds derived from plant, animal, and microbial sources. These compounds encompass a broad range of chemical classes with distinct physicochemical properties, biological activities, pharmacokinetic profiles, and therapeutic applications. As illustrated in Figure 2, representative naturally occurring compounds exhibit considerable structural diversity, reflecting their varied origins and mechanisms of action. Classification of natural products is commonly based on their biological source and chemical architecture, providing a framework for understanding their molecular targets, metabolic fate, and potential roles in disease prevention and management.

In addition to their nutritional functions, many natural compounds act as pleiotropic bioactive molecules capable of modulating a wide range of molecular targets and signaling networks, including redox-sensitive pathways, inflammatory mediators, metabolic regulators, and gene-expression programs. Advances in nutraceutical research have further highlighted their involvement in gut–organ axis communication, epigenetic regulation, immune modulation, and host–microbiome interactions, underscoring their potential contributions to chronic disease prevention and adjunctive therapeutic strategies [1,13,19,20].

Given their considerable chemical and biological diversity, natural products encompass multiple classes of compounds that differ substantially in their mechanisms of action, pharmacokinetic properties, and clinical applications. Accordingly, this section provides an overview of the principal categories of natural products commonly utilized as dietary supplements, with particular emphasis on their bioactive constituents, major pharmacological activities, and key bioavailability limitations. A comprehensive classification of natural products, together with representative compounds and their primary dietary sources, is presented in Table 1.

### 3.1. Plant-Derived Compounds

Plant-derived compounds constitute the largest and most extensively studied group of natural products used in nutritional and therapeutic applications. These compounds are predominantly secondary metabolites that evolved as plant defense molecules against pathogens, UV radiation, and environmental stress, while simultaneously exhibiting significant pharmacological activities in humans [7,8,9,10]. Despite their broad bioactivity, a major limitation of many plant-derived compounds is their low oral bioavailability due to poor absorption, extensive first-pass metabolism, and rapid systemic elimination. To address these challenges, modern delivery strategies such as nanoencapsulation, solid lipid nanoparticles, and phytosome-based formulations have been developed to enhance stability and systemic exposure [7,8,9,10].

#### 3.1.1. Polyphenols

Polyphenols are a broad class of phytochemicals characterized by multiple phenolic hydroxyl groups and aromatic ring structures. They are widely distributed in fruits, vegetables, tea, coffee, cocoa, whole grains, herbs, and wine. Major subclasses include phenolic acids, flavonoids, stilbenes, lignans, and tannins. Beyond their classical antioxidant capacity via reactive oxygen species (ROS) scavenging, polyphenols act as modulators of cellular signaling networks, transcription factors, and epigenetic regulators, thereby exerting systemic biological effects.

Mechanistically, polyphenols regulate several intracellular pathways, including NF-κB, MAPK, PI3K/Akt, and nuclear factor erythroid 2-related factor 2 (Nrf2) signaling, contributing to anti-inflammatory, cardioprotective, neuroprotective, and anticancer effects [4,12]. In addition, polyphenols influence mitochondrial biogenesis, endothelial nitric oxide synthase (eNOS) activity, and lipid metabolism, thereby improving vascular function and metabolic homeostasis [21,22]. Recent evidence also highlights their role in modulating gut microbiota composition and microbial-derived metabolite production, leading to the formation of bioactive phenolic metabolites with enhanced systemic activity and interindividual variability in response [21,22].

#### 3.1.2. Flavonoids

Flavonoids represent one of the most abundant and structurally diverse subclasses of polyphenols, encompassing flavonols, flavones, flavanones, isoflavones, flavan-3-ols (catechins), and anthocyanins. These compounds are widely recognized for their antioxidant, anti-inflammatory, vasodilatory, and anti-thrombotic properties. In cardiovascular systems, flavonoids enhance endothelial nitric oxide bioavailability, reduce oxidative stress, and inhibit vascular smooth muscle proliferation, collectively improving vascular homeostasis.

Flavonoids also regulate glucose and lipid metabolism through modulation of AMP-activated protein kinase (AMPK), peroxisome proliferator-activated receptors (PPARs), and insulin signaling pathways. At the cellular level, they influence apoptosis, autophagy, and cell cycle arrest, thereby contributing to their anticancer potential [23,24]. Furthermore, flavonoids exert immunomodulatory effects by regulating cytokine secretion, macrophage polarization, and T-cell differentiation, making them relevant in chronic inflammatory and metabolic disorders [23,24].

#### 3.1.3. Terpenoids

Terpenoids (isoprenoids) constitute a structurally diverse class of natural compounds derived from isoprene (C5) units, forming monoterpenes, sesquiterpenes, diterpenes, triterpenes, and tetraterpenes (e.g., carotenoids). These compounds are widely present in essential oils, medicinal plants, and dietary sources such as fruits, vegetables, and herbs.

Terpenoids exhibit a marked spectrum of biological activities, including anti-inflammatory, antimicrobial, antiviral, antioxidant, and anticancer effects. Mechanistically, they regulate inflammatory mediators such as cytokines, cyclooxygenase-2 (COX-2), and inducible nitric oxide synthase (iNOS), while also modulating oxidative stress pathways and cell proliferation signaling [25]. Carotenoids, including β-carotene, lycopene, lutein, and zeaxanthin, are particularly important due to their ability to quench singlet oxygen and reduce lipid peroxidation in biological membranes [25]. In addition, carotenoids contribute to immune regulation, ocular health, and potential protection against age-related degenerative diseases [25].

### 3.2. Animal-Derived Bioactive Compounds

Animal-derived bioactive compounds represent an important class of nutritional supplements with significant relevance in cardiovascular, neurological, musculoskeletal, and inflammatory conditions. These compounds often act through membrane incorporation, lipid mediator biosynthesis, or receptor-mediated peptide signaling, thereby influencing systemic physiological processes and inflammatory resolution pathways.

#### 3.2.1. Omega-3 Fatty Acids

Omega-3 polyunsaturated fatty acids (PUFAs), primarily eicosapentaenoic acid (EPA) and docosahexaenoic acid (DHA), are predominantly obtained from marine sources such as fish oil, krill oil, and microalgae. These fatty acids are essential structural components of phospholipid membranes and play a critical role in maintaining membrane fluidity, receptor function, and intracellular signaling.

Beyond structural roles, omega-3 PUFAs exert potent anti-inflammatory effects by competitively inhibiting arachidonic acid-derived eicosanoid synthesis and promoting the biosynthesis of specialized pro-resolving mediators (SPMs), including resolvins, protectins, and maresins [26,27]. These lipid mediators actively orchestrate the resolution phase of inflammation rather than merely suppressing inflammatory initiation. Clinical and mechanistic evidence supports their beneficial roles in cardiovascular disease, neurodegenerative disorders, psychiatric conditions, and chronic inflammatory states [26,27].

#### 3.2.2. Bioactive Peptides

Bioactive peptides are short amino acid sequences (typically 2–20 residues) derived from enzymatic hydrolysis or gastrointestinal digestion of dietary proteins from milk, eggs, fish, and meat. These peptides exhibit physiological activities beyond basic nutrition and are increasingly recognized as functional food components [28].

Their biological activities include antihypertensive effects via angiotensin-converting enzyme (ACE) inhibition, antioxidant activity through radical scavenging and metal chelation, antimicrobial effects against pathogenic bacteria, and immunomodulatory functions [27,28]. Additionally, bioactive peptides may enhance mineral absorption, modulate gut microbiota composition, and regulate satiety and metabolic signaling pathways, highlighting their importance in nutraceutical development and functional food design [29].

### 3.3. Microbial-Derived Products

Microbial-derived products have gained substantial attention due to their central role in host–microbiome interactions and systemic disease modulation. These compounds influence host physiology through metabolic, immunological, and neuroendocrine pathways, forming a key component of the gut–brain–immune axis and contributing to metabolic homeostasis.

#### 3.3.1. Probiotics

Probiotics are defined as live microorganisms that, when administered in adequate amounts, confer a health benefit to the host. Common probiotic genera include Lactobacillus, Bifidobacterium, and Saccharomyces. These microorganisms contribute to intestinal microbial balance by competing with pathogens, enhancing epithelial barrier integrity, producing antimicrobial compounds, and modulating mucosal and systemic immune responses [30,31].

Clinical studies demonstrate beneficial effects of probiotics in gastrointestinal disorders such as irritable bowel syndrome, inflammatory bowel disease, and antibiotic-associated diarrhea [30,31]. Emerging evidence also suggests systemic effects, including modulation of metabolic parameters, cholesterol reduction, improvement in insulin sensitivity, and regulation of the gut–brain axis affecting mood and cognitive function [30,31].

#### 3.3.2. Postbiotics and Microbial Metabolites

Postbiotics refer to non-viable microbial cells, structural components, and metabolic byproducts that confer health benefits to the host. These include short-chain fatty acids (SCFAs) such as butyrate, propionate, and acetate, as well as bacterial cell wall fragments, enzymes, and peptides. Unlike probiotics, postbiotics are non-living, offering advantages in stability, safety, and controlled therapeutic formulation.

SCFAs are key metabolic mediators that regulate intestinal epithelial integrity, immune homeostasis, and energy metabolism [32,33]. Butyrate serves as a primary energy source for colonocytes and functions as an epigenetic regulator through histone deacetylase inhibition, thereby modulating gene expression involved in inflammation and barrier function [32,33]. Increasing evidence suggests that postbiotics may play a role in the prevention and management of metabolic syndrome, inflammatory diseases, colorectal cancer, and neurodegenerative disorders through systemic signaling and gut–organ axis communication [32,33].

## 4. Mechanisms of Action of Natural Products: A Comparative Mechanistic Analysis

Natural products derived from plant, animal, and microbial sources exert their biological activities through overlapping yet mechanistically distinct pathways. Although all three categories share core functional properties—particularly antioxidant, anti-inflammatory, and immunomodulatory effects—their primary molecular targets, pharmacodynamic profiles, and systems-level effects differ substantially [3,10,11,14,19,20,25,26,27,28,29,30,31,32,33]. These differences reflect their divergent chemical structures, metabolic fates, and evolutionary biological roles [3,10,11,14,19,20,25,26,27,28,29,30,31,32,33]. A mechanistic comparison is therefore essential for understanding their supplementary contributions to human health and for guiding rational design of multi-component nutraceutical interventions.

Overall, plant-derived compounds predominantly act as redox-active signaling modulators with broad transcriptional effects; animal-derived bioactive compounds function primarily through lipid mediator biology and membrane-associated signaling; and microbial-derived products exert systemic effects via host–microbiome interactions, metabolic reprogramming, and epigenetic regulation.

### 4.1. Antioxidant Mechanisms: Chemical Scavenging vs. Endogenous Defense Activation vs. Metabolic Redox Control

Plant-derived phytochemicals, particularly polyphenols, flavonoids, and carotenoids, represent the most extensively characterized class of direct chemical antioxidants. Their antioxidant activity is primarily attributed to electron-donating hydroxyl groups and conjugated π-electron systems, which enable efficient scavenging of reactive oxygen and nitrogen species (ROS/RNS) and chelation of transition metal ions involved in Fenton-type reactions [34,35]. However, growing evidence suggests that their biological efficacy in vivo depends less on direct radical scavenging and more on their ability to activate endogenous antioxidant pathways, particularly through modulation of Nrf2, NF-κB, MAPK, and other redox-sensitive signaling networks [34,35]. Consequently, plant-derived compounds provide a dual mode of action, combining immediate chemical antioxidant activity with broader transcriptional regulation of cellular defense systems.

In contrast, animal-derived omega-3 fatty acids exhibit a fundamentally different antioxidant profile. EPA and DHA possess limited direct radical-scavenging capacity because they lack the phenolic structures characteristic of plant antioxidants. Instead, their antioxidant effects are mediated indirectly through incorporation into cellular membranes, alteration of membrane fluidity and phospholipid composition, modulation of membrane-associated signaling pathways, and generation of SPMs [36,37]. By promoting the resolution of inflammation, omega-3 fatty acids reduce secondary ROS production from activated neutrophils, macrophages, and other immune cells. Compared with plant phytochemicals, animal-derived bioactive compounds therefore exert weaker direct antioxidant effects but stronger regulation of inflammation-driven oxidative damage [36,37].

Microbial-derived metabolites, particularly SCFAs, represent a third and mechanistically distinct antioxidant strategy based on metabolic and epigenetic regulation [38,39]. Rather than directly scavenging free radicals or modifying membrane composition, SCFAs such as butyrate, propionate, and acetate enhance mitochondrial efficiency, improve cellular energy metabolism, and stimulate endogenous antioxidant defenses through histone deacetylase (HDAC) inhibition and activation of Nrf2-dependent gene expression [38,39]. As a result, microbial metabolites generally produce slower but more sustained effects on redox homeostasis by remodeling transcriptional programs involved in antioxidant protection, inflammation control, and cellular metabolism.

### 4.2. Anti-Inflammatory Mechanisms: Enzymatic Inhibition vs. Pro-Resolving Signaling vs. Microbiota-Mediated Immunoregulation

Plant-derived compounds primarily exert anti-inflammatory effects by suppressing pro-inflammatory signaling pathways. Polyphenols and terpenoids inhibit NF-κB and MAPK signaling (extracellular-signal-regulated kinase [ERK], c-Jun N-terminal kinase [JNK], and p38), thereby reducing the expression of pro-inflammatory cytokines, chemokines, and adhesion molecules, while also downregulating COX-2 and lipoxygenase (LOX) activity and the subsequent production of prostaglandins and leukotrienes [40,41]. Consequently, plant-derived compounds mainly act by limiting the initiation and amplification of inflammatory responses.

In contrast, animal-derived omega-3 fatty acids function through a distinct pro-resolving mechanism. EPA and DHA serve as precursors of SPMs, including resolvins, protectins, and maresins, which actively terminate inflammation by enhancing macrophage efferocytosis, reducing neutrophil infiltration, and promoting tissue repair [42,43]. Thus, unlike plant phytochemicals that primarily suppress inflammatory signaling, omega-3 fatty acids facilitate the active resolution phase of inflammation [42,43].

Microbial-derived compounds regulate inflammation through host–microbiome immunometabolic interactions. SCFAs modulate immune responses via G-protein-coupled receptor activation (GPR41 and GPR43) and HDAC inhibition, promoting regulatory T-cell differentiation while suppressing pro-inflammatory Th17 responses [38,44]. In addition, probiotics and postbiotics indirectly reduce systemic inflammation by strengthening intestinal barrier integrity and limiting lipopolysaccharide (LPS) translocation and metabolic endotoxemia [45].

### 4.3. Immunomodulatory Mechanisms: Transcriptional Regulation vs. Lipid Signaling vs. Gut-Immune Axis Programming

Plant-derived compounds modulate immunity primarily through transcriptional regulation of NF-κB, signal transducer and activator of transcription (STAT), and activator protein-1 (AP-1) signaling pathways, enabling context-dependent suppression of excessive inflammation while supporting host defense mechanisms [46]. In contrast, animal-derived bioactive compounds, particularly bioactive peptides and omega-3 fatty acids, regulate immune responses through receptor-mediated signaling and lipid mediator class switching, promoting a shift from pro-inflammatory to pro-resolving immune states [42,47].

Microbial-derived products exert immunomodulatory effects through direct interactions with the gut-associated immune system. Probiotics influence dendritic cell maturation and T-cell differentiation via interactions with Gut-Associated Lymphoid Tissue (GALT), whereas postbiotics and SCFAs act as metabolic signals linking microbial activity to systemic immune regulation [32,33,45]. Compared with plant- and animal-derived compounds, microbial metabolites generally produce more sustained effects on mucosal immunity and immune tolerance through microbiome-dependent and epigenetic mechanisms [32,33,45].

### 4.4. Antimicrobial Mechanisms: Direct Microbicidal Action vs. Host-Mediated Defense vs. Ecological Resistance

Plant-derived natural products exhibit the strongest direct antimicrobial activity among natural product classes, acting through membrane disruption, inhibition of nucleic acid synthesis, and interference with essential microbial enzymes [48]. Terpenoids and essential oils are particularly effective because their lipophilic properties facilitate membrane permeabilization and loss of cellular homeostasis [48]. Consequently, plant-derived compounds primarily target pathogens directly and can exert wide-ranging-spectrum antimicrobial effects.

In contrast, animal-derived compounds generally contribute to antimicrobial defense indirectly by enhancing host immune competence, strengthening barrier function, and modulating inflammatory microenvironments [49]. Although certain bioactive peptides possess direct antimicrobial properties, including bacteriocin-like activity, their overall antimicrobial efficacy is typically less pronounced and more host-mediated than that of plant-derived compounds [49].

Microbial-derived products employ a fundamentally different strategy based on ecological regulation rather than pathogen eradication. Probiotics suppress pathogen colonization through competitive adhesion, nutrient competition, and bacteriocin production, while postbiotics enhance epithelial barrier integrity and stimulate host antimicrobial peptide expression [50,51]. Unlike plant-derived antimicrobials that directly eliminate pathogens, microbial-derived interventions reshape the microbial ecosystem to favor beneficial communities and reduce pathogen fitness [50,51].

### 4.5. Signaling Pathway Modulation: Multi-Level Network Regulation Across Biological Systems

Natural products converge on several conserved signaling pathways, although their primary points of intervention differ substantially. Plant-derived compounds predominantly target transcriptional and stress-response networks, including NF-κB, MAPK, phosphatidylinositol-3-kinase (PI3K)/Akt, and Nrf2, thereby regulating inflammation, oxidative stress, apoptosis, and cellular proliferation [51,52]. In contrast, animal-derived omega-3 fatty acids primarily influence lipid-mediated signaling pathways, modulating eicosanoid biosynthesis and SPMs networks to shift cellular responses from inflammatory activation toward resolution and tissue repair [53].

Microbial-derived metabolites act through a third, mechanistically distinct pathway involving epigenetic, metabolic, and neuroimmune regulation. Through HDAC inhibition, GPCR activation, and modulation of the gut–brain axis, SCFAs influence neurotransmitter production, vagal signaling, hypothalamic function, and systemic immune responses [38,39,54]. Compared with plant-derived compounds, which mainly regulate intracellular transcriptional programs, and animal-derived bioactive compounds, which primarily modulate lipid signaling cascades, microbial metabolites exert broader effects on host metabolism and inter-organ communication networks [38,39,54].

### 4.6. Integrated Comparative Perspective

**Figure 3:**
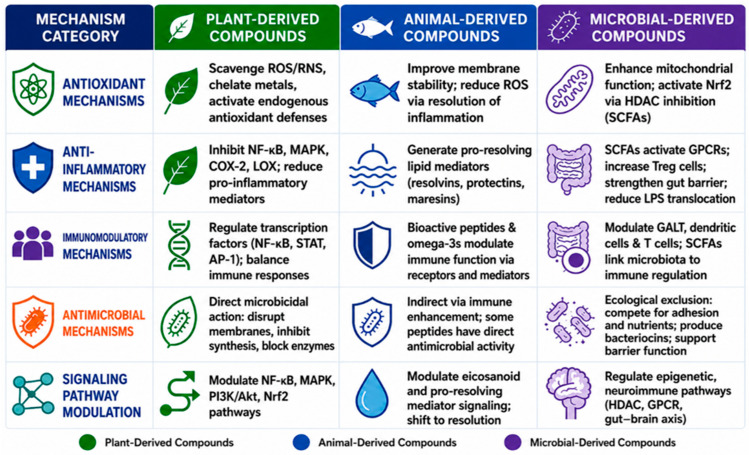
Principal molecular mechanisms underlying the biological activities of major classes of natural compounds.

Although plant-, animal-, and microbial-derived natural products share antioxidant, anti-inflammatory, and immunomodulatory properties, they differ substantially in their primary mechanisms of action. In Figure 3, the principal molecular mechanisms underlying the biological activities of major classes of natural compounds are depicted. Plant-derived compounds predominantly modulate intracellular signaling networks and redox-sensitive pathways, animal-derived bioactive compounds primarily influence membrane-associated and lipid-mediator signaling, whereas microbial-derived products exert many of their effects through host–microbiome interactions, metabolic regulation, and epigenetic mechanisms. Together, these observations suggest that distinct classes of natural products may influence health through partially overlapping but mechanistically diverse pathways promoting broader and more sustained therapeutic effects than single-compound interventions in complex chronic diseases. Such mechanistic diversity may contribute to the broad spectrum of biological activities attributed to natural products and supports continued investigation of their potential roles in disease prevention and management.

Notably, the mechanistic overlap among diverse disease states provides a strong rationale for the broad scope of the present review. Because oxidative stress, chronic inflammation, impaired immune regulation, and metabolic disturbances contribute to the pathogenesis of numerous chronic and acute diseases, natural products that target these interconnected pathways may exhibit therapeutic relevance across multiple clinical settings.

## 5. Therapeutic Applications Across Disease Categories

Dietary bioactive compounds derived from plant, animal, and microbial sources exhibit pleiotropic biological activities across a broad spectrum of pathological conditions. Their mechanistic relevance is primarily attributed to coordinated modulation of interconnected signaling networks governing inflammation, oxidative stress, metabolic regulation, epigenetic remodeling, and neuroimmune communication. Key pathways implicated include NF-κB, MAPK, PI3K/Akt, Nrf2, Janus kinase (JAK)/STAT, and gut–brain axis–associated signaling systems.

However, although these compounds are frequently conceptualized as multiple targeting modulators of systems-level homeostasis, the strength of evidence supporting clinical efficacy varies substantially across disease domains. In general, cardiometabolic conditions exhibit the most consistent translational support, whereas neurodegenerative and oncological applications remain largely preclinical or adjunctive, reflecting differences in disease complexity, intervention timing, and bioavailability constraints [55].

Notably, the disease categories discussed in the following sections should not be viewed as independent entities but rather as examples of clinical conditions in which common pathophysiological mechanisms are operative. Accordingly, the objective is not to provide exhaustive disease-specific reviews but to illustrate how shared biological activities of natural products may translate into therapeutic benefits across diverse pathological contexts. Moreover, it should be noted that although mechanistic and preclinical evidence provides important biological rationale for the therapeutic use of natural products, clinical translation remains variable. Therefore, interpretation of the available evidence requires consideration of effect size, reproducibility, participant heterogeneity, intervention characteristics, and the existence of studies reporting null or inconsistent findings. In Table 2, preclinical evidence of natural compounds, their key molecular mechanisms, and biological outcomes in experimental models is summarized.

### 5.1. Cardiovascular Diseases (CVDs)

CVDs are the leading cause of global mortality and encompass a diverse group of disorders affecting the heart and blood vessels [56]. Their development is driven by both modifiable risk factors (e.g., hypertension, dyslipidemia, smoking, diabetes, and obesity) and non-modifiable factors such as age and genetics [56]. Atherosclerosis, the central pathogenic process, is characterized by endothelial dysfunction, lipid accumulation, inflammation, and plaque formation, which may ultimately lead to plaque rupture, thrombosis, myocardial infarction, or stroke [57]. These multifactorial mechanisms highlight the importance of preventive and targeted therapeutic approaches.

Natural compounds exert cardioprotective effects through interconnected actions on lipid metabolism, inflammation, oxidative stress, endothelial function, thrombosis, and gut microbiota–host interactions, thereby modulating atherosclerosis and other key pathways involved in CVD pathogenesis.

#### 5.1.1. Integrated Mechanisms of Cardiovascular Protection

Natural compounds exert cardioprotective effects through complementary actions on lipid metabolism, inflammation, oxidative stress, endothelial function, thrombosis, and gut microbiota; however, major nutraceutical classes differ considerably in their molecular targets, lipid-lowering potency, cardiometabolic effects, and strength of clinical evidence, as discussed below. Thus, they range from drug-like agents with defined molecular targets to broader multiple targeting dietary modulators.

Among lipid-lowering interventions, red yeast rice exhibits the greatest potency because monacolin K directly inhibits 3-hydroxy-3-methylglutaryl coenzyme A (HMG-CoA) reductase, producing statin-like low-density lipoprotein (LDL) reductions [58]. Berberine lowers LDL indirectly through ERK/JNK-mediated upregulation of LDL receptors while also improving glucose metabolism [59]. Plant sterols and dietary fibers reduce cholesterol absorption and increase bile acid excretion, producing moderate but consistent lipid improvements [60]. In contrast, polyphenols such as epigallocatechin gallate (EGCG) and resveratrol exert weaker lipid effects but broader cardiometabolic actions through AMPK activation [61].

Anti-inflammatory effects are mediated primarily by polyphenols including curcumin, resveratrol, quercetin, and EGCG, which suppress NF-κB and nucleotide-binding domain, leucine-rich–containing family, pyrin domain–containing-3 (NLRP3) signaling and reduce pro-inflammatory cytokines [62]. Berberine and resveratrol additionally link metabolic and inflammatory regulation through AMPK activation [62]. Oxidative stress is reduced mainly through Nrf2–ARE activation by compounds such as curcumin, sulforaphane, EGCG, CoQ10, and cocoa flavanols, enhancing endogenous antioxidant defenses, although clinical benefits are generally less consistent than those observed for lipid-lowering agents [63].

For vascular function, beetroot nitrates produce the strongest acute effects by increasing nitric oxide bioavailability, whereas cocoa flavanols exert moderate eNOS-mediated benefits and L-arginine/citrulline show weaker effects due to metabolic limitations [64]. Anti-thrombotic actions of garlic, omega-3 fatty acids, and Ginkgo biloba are generally modest and substantially less potent than conventional antiplatelet therapies [65].

Gut microbiota modulation represents a distinct mechanism whereby dietary fibers and polyphenol-rich foods promote SCFAs production, reduce trimethylamine N-oxide (TMAO) formation, and improve metabolic and inflammatory signaling [66]. Unlike compounds targeting single pathways, microbiota-directed interventions exert more extensive systems-level effects but show greater interindividual variability [66]. This variability is ascribed to the fact that their efficacy depends on host-specific factors, including baseline gut microbiota composition, microbial metabolic capacity, dietary patterns, medication use, and genetic background, all of which may influence the generation of bioactive microbial metabolites and downstream physiological responses [66].

Overall, red yeast rice and omega-3 fatty acids demonstrate the strongest clinical evidence. Berberine and plant sterols provide moderate metabolic effects, whereas polyphenols, flavanols, and microbiota-targeted interventions offer broader but generally less potent cardioprotective effects. Consequently, natural compounds are best viewed as complementary multiple targeting modulators rather than replacements for established cardiovascular therapies.

#### 5.1.2. Preclinical Evidence

Preclinical evidence from in vitro and animal studies demonstrates that natural compounds exert cardioprotective effects through diverse pathways, although their potency and mechanisms vary considerably. Lipid-lowering agents generally produce the strongest direct metabolic effects, whereas polyphenols and microbiota-targeted interventions exhibit broader pleiotropic actions.

In lipid metabolism, red yeast rice (monacolin K) shows the most potent cholesterol-lowering activity through direct HMG-CoA reductase inhibition, while berberine enhances LDL receptor expression via ERK/JNK signaling and improves glucose metabolism. Plant sterols (β-sitosterol) and dietary fibers (β-glucans, pectin) reduce cholesterol absorption and increase bile acid excretion, resulting in moderate but consistent lipid improvements [67].

Polyphenols such as curcumin, resveratrol, EGCG, and quercetin exert strong anti-inflammatory effects by suppressing NF-κB, NLRP3, COX-2, and iNOS signaling and reducing pro-inflammatory cytokines, whereas berberine links inflammatory and metabolic regulation through AMPK activation [68]. Antioxidant protection is mediated primarily through Nrf2–ARE activation by sulforaphane, curcumin, EGCG, CoQ10, and cocoa flavanols, increasing endogenous antioxidant defenses and reducing oxidized LDL and lipid peroxidation [63].

Endothelial function is enhanced through nitric oxide (NO)-dependent mechanisms. Beetroot nitrates produce the strongest effects via the nitrate–nitrite–NO pathway, while cocoa flavanols stimulate eNOS activity and L-arginine/citrulline provide indirect NO support [69]. In contrast, anti-thrombotic effects are generally modest; garlic-derived allicin, omega-3 fatty acids (EPA/DHA), and Ginkgo biloba reduce platelet aggregation but remain less potent than conventional antiplatelet agents [70,71].

Gut microbiota modulation represents a distinct systems-level mechanism. Polyphenol-rich foods and dietary fibers increase SCFAs production, improve bile acid metabolism, and reduce TMAO formation, thereby influencing inflammation, lipid metabolism, and atherosclerosis progression [72].

Overall, red yeast rice exhibits the strongest lipid-lowering activity, polyphenols provide the most robust anti-inflammatory and antioxidant effects, beetroot nitrates are the most effective enhancers of endothelial function, and microbiota-targeted interventions offer the broadest systemic benefits. These findings support the multiple targeting cardioprotective potential of natural compounds while highlighting differences in potency, mechanism specificity, and translational relevance [63,67,68,69,70,71,72].

#### 5.1.3. Clinical Evidence

Clinical evidence regarding the efficacy of natural products in the prevention and management of CVDs is derived primarily from RCTs, systematic reviews, and meta-analyses evaluating effects on cardiovascular risk factors and, less frequently, hard clinical endpoints. Overall, the evidence reveals a clear hierarchy of efficacy: interventions targeting established risk factors such as dyslipidemia and endothelial dysfunction generally demonstrate more consistent benefits than those aimed at inflammation, oxidative stress, or microbiome modulation. Moreover, only a limited number of interventions have demonstrated reproducible reductions in major adverse cardiovascular events.

Among lipid-lowering strategies, red yeast rice containing monacolin K exhibits the strongest and most pharmacologically comparable effects to conventional therapy, producing LDL-cholesterol (LDL-C) reductions of approximately 15–25%, similar to low-intensity statins in selected populations [73]. In comparison, plant sterols and stanols achieve more modest but highly reproducible LDL-C reductions of 5–15% through inhibition of intestinal cholesterol absorption, with efficacy reaching a plateau at intakes above 2–3 g/day [74]. Dietary fibers such as oat β-glucans and psyllium generally produce smaller lipid improvements than red yeast rice but possess a stronger safety profile and broader guideline support as adjunctive therapies [75]. Berberine occupies an intermediate position, providing simultaneous improvements in lipid and glycemic parameters; however, its evidence base remains less robust due to small study populations, short intervention periods, and methodological heterogeneity [59,76].

Evidence for omega-3 fatty acids highlights the importance of formulation and dosage. Mixed EPA/DHA preparations have produced inconsistent cardiovascular outcomes across trials, whereas high-dose purified EPA demonstrated significant reductions in major adverse cardiovascular events in high-risk patients in studies such as REDUCE-IT [77]. Consequently, omega-3 fatty acids represent one of the clearest examples in nutraceutical research where clinical efficacy depends strongly on formulation, treatment intensity, and patient characteristics rather than on the nutrient class itself [77].

For blood pressure regulation, beetroot-derived nitrates, garlic, and Hibiscus sabdariffa extracts all demonstrate antihypertensive effects, although their magnitude is generally modest compared with conventional antihypertensive medications [78]. Beetroot nitrates consistently improve NO bioavailability and vascular function, whereas garlic appears most effective in untreated hypertensive individuals. Hibiscus may provide additional benefits through ACE-inhibitory and diuretic-like mechanisms, but the supporting evidence is more heterogeneous and less standardized [78].

Endothelial function represents one of the most responsive vascular endpoints in nutraceutical research. Cocoa flavanols consistently improve flow-mediated dilation (FMD) through enhanced eNOS activity and vascular reactivity [64]. However, Mediterranean dietary patterns provide broader cardioprotective effects, combining improvements in endothelial function, lipid metabolism, inflammation, and metabolic health. Large-scale trials such as PREDIMED demonstrated not only favorable biomarker changes but also significant reductions in cardiovascular events, making Mediterranean dietary interventions substantially better supported than most individual nutraceuticals [79].

In contrast, clinical evidence for anti-inflammatory and antioxidant interventions remains considerably weaker. Although polyphenol-rich diets and omega-3 fatty acids consistently reduce surrogate biomarkers such as C-reactive protein (CRP), oxidized LDL, and inflammatory cytokines, translation into measurable reductions in cardiovascular events has been less convincing [80]. This limitation is particularly evident for isolated antioxidant supplementation, where vitamin E and vitamin C monotherapy have repeatedly failed to demonstrate meaningful cardiovascular benefit despite strong mechanistic rationale, highlighting the superiority of whole-food and multi-component dietary approaches over reductionist antioxidant strategies [81,82].

Microbiome-targeted interventions represent an emerging but less mature area of cardiovascular prevention. High-fiber diets and polyphenol-rich foods reduce circulating TMAO levels and increase SCFAs production, changes associated with improved cardiometabolic profiles [83,84]. However, compared with lipid-lowering or blood-pressure-lowering interventions, evidence linking microbiome modulation directly to reductions in cardiovascular events remains largely indirect and requires long-term validation [83,84].

Clinical evidence supports a hierarchy of efficacy among natural interventions. Mediterranean dietary patterns and high-dose purified EPA provide the strongest evidence for reducing cardiovascular events, while red yeast rice exhibits the most potent lipid-lowering effects among individual nutraceuticals [73,77,79]. Plant sterols, dietary fibers, cocoa flavanols, nitrate-rich vegetables, and berberine consistently improve cardiometabolic risk factors, although evidence for berberine remains less mature [74,75,76]. In contrast, polyphenols, curcumin, resveratrol, garlic, Ginkgo biloba, and microbiota-targeted interventions primarily improve surrogate biomarkers and intermediate outcomes, with limited evidence for cardiovascular event reduction. Overall, natural products appear most effective as adjunctive, multiple targeting strategies complementing established cardiovascular therapies and healthy dietary patterns.

Notably, the stronger evidence supporting Mediterranean dietary patterns and high-dose EPA is largely attributable to findings from large-scale randomized controlled trials demonstrating reductions in major adverse cardiovascular events, whereas most nutraceutical interventions have primarily shown benefits on surrogate cardiometabolic markers, including lipid concentrations, blood pressure, oxidative stress, and inflammatory biomarkers, with less consistent evidence regarding cardiovascular event reduction [73,74,75,76,77].

Moreover, it should be noted that although improvements in inflammatory, oxidative stress, endothelial, and microbiome-related biomarkers have been consistently reported for several nutraceuticals, translation into significant reductions in cardiovascular events has been limited. This may reflect the multifactorial nature of cardiovascular disease, the modest magnitude of biomarker changes achieved, the variability in bioavailability and individual responsiveness, and the relative scarcity of large, long-term randomized controlled trials evaluating hard cardiovascular outcomes.

### 5.2. Metabolic Disorders

#### 5.2.1. Diabetes

Type 2 diabetes mellitus (T2DM) is a chronic metabolic disorder characterized by insulin resistance, progressive β-cell dysfunction, and persistent hyperglycemia. Closely associated with obesity, sedentary lifestyles, and population aging, T2DM is accompanied by chronic inflammation, oxidative stress, dyslipidemia, and endothelial dysfunction, which contribute to microvascular and macrovascular complications. Owing to its multifactorial pathophysiology, nutritional interventions, including natural bioactive compounds and microbiome-targeted strategies, have attracted increasing interest as supplementary approaches for improving glycemic control and metabolic health.

##### Mechanistic Comparison

Anti-diabetic effects of natural compounds arise through overlapping regulation of insulin signaling, inflammation, oxidative stress, and energy metabolism, although their mechanisms differ substantially in specificity and potency. Both plant-derived compounds and microbiota-derived metabolites improve glucose homeostasis through activation of PI3K/Akt signaling, inhibition of NF-κB-mediated inflammation, and enhancement of Nrf2-dependent antioxidant defenses, thereby promoting glucose uptake, reducing hepatic glucose production, and preserving β-cell function [85,86].

However, SCFAs possess a distinct mechanistic advantage because they act as endogenous signaling molecules rather than indirect metabolic modulators. By activating G-protein-coupled receptors (GPR41, GPR43, and GPR109A), SCFAs directly stimulate incretin secretion such as glucagon-like peptide-1 (GLP-1) and peptide YY (PYY), suppress hepatic gluconeogenesis, and regulate sympathetic nervous system activity, providing a direct link between gut microbial fermentation and systemic glucose control [87]. Furthermore, their ability to inhibit HDACs adds an epigenetic dimension to metabolic regulation, enabling coordinated improvements in insulin sensitivity, inflammation, and energy metabolism [88].

In contrast, polyphenols primarily exert indirect insulin-sensitizing effects through modulation of upstream cellular stress and inflammatory pathways. Compounds such as resveratrol, quercetin, curcumin, and EGCG regulate NF-κB, MAPK, and AMPK signaling, leading to reduced cytokine production, improved mitochondrial function, and enhanced peripheral insulin responsiveness [89,90]. Compared with SCFAs, however, their metabolic effects are generally less direct and more dependent on bioavailability, microbial biotransformation, and inter-individual differences in gut microbiota composition, factors that contribute to greater variability in clinical outcomes [89,90].

Overall, SCFAs and polyphenols both improve glycemic control but through distinct mechanisms. SCFAs exert more targeted metabolic effects via direct receptor-mediated (GPR41, GPR43, GPR109A) and epigenetic regulation, whereas polyphenols act primarily as wide-ranging anti-inflammatory and redox-modulating agents affecting multiple pathways involved in type 2 diabetes pathogenesis [87,88,89,90].

##### Preclinical Evidence

Animal studies consistently demonstrate that polyphenols such as curcumin, resveratrol, and quercetin improve glycemic control through modulation of PI3K/Akt and AMPK signaling, suppression of NF-κB-mediated inflammation, and activation of Nrf2-dependent antioxidant defenses [85,86,91]. Beyond improving fasting glucose, hemoglobin A1c (HbA1c), insulin sensitivity, and glucose tolerance, these compounds influence hepatic gluconeogenesis, adipocyte differentiation, lipid metabolism, and skeletal muscle glucose uptake, thereby targeting various components of metabolic syndrome simultaneously [85,86,91]. Several studies further demonstrate preservation of pancreatic β-cell mass and function through attenuation of oxidative stress, endoplasmic reticulum stress, and apoptosis, mechanisms that may slow progression from insulin resistance to overt diabetes [85,86]. In addition, polyphenols enhance mitochondrial biogenesis and function, improve cellular energy metabolism, and reduce ectopic lipid accumulation in liver and muscle tissues, thereby contributing to improved systemic metabolic homeostasis [86,91].

Despite these wide-ranging mechanistic effects, the magnitude of metabolic improvement varies considerably among compounds. Resveratrol appears particularly effective in activating AMPK and mitochondrial regulatory pathways, whereas curcumin exerts stronger anti-inflammatory effects through NF-κB inhibition, and quercetin demonstrates potent antioxidant and endothelial-protective properties [85,86]. However, their efficacy is highly dependent on formulation, dose, intervention duration, and bioavailability, with poor absorption, extensive first-pass metabolism, and rapid systemic clearance often limiting translational consistency. Advanced delivery systems, including nanoencapsulation, liposomal formulations, and phospholipid complexes, frequently enhance efficacy in animal models by improving tissue exposure and pharmacokinetic stability [85,86,91]. Variability in experimental diets, diabetes induction protocols, species differences, and disease severity further contributes to heterogeneous outcomes across studies [91].

In contrast, probiotic interventions exert their effects indirectly through modulation of the gut microbiota–host axis and generally display greater variability than polyphenols [92,93]. Their metabolic benefits are mediated through restoration of microbial diversity, enhancement of gut barrier integrity, suppression of endotoxemia, and increased production of beneficial metabolites such as SCFAs, which regulate glucose metabolism through GPR41/GPR43 signaling and incretin secretion [92,93]. Certain probiotic strains also reduce systemic inflammation by decreasing lipopolysaccharide (LPS)-induced immune activation and improving intestinal permeability, thereby attenuating one of the key drivers of insulin resistance [92].

The efficacy of probiotic interventions depends strongly on strain specificity, with different Lactobacillus and Bifidobacterium species producing distinct metabolic responses, while multi-strain formulations may generate synergistic or antagonistic interactions [92,93]. Unlike polyphenols, whose molecular targets and signaling pathways are relatively well characterized, probiotic outcomes are heavily influenced by baseline microbiota composition, colonization efficiency, dietary background, dosage, supplementation duration, host genetics, and metabolic status [92,93]. Consequently, probiotic interventions often exhibit greater inter-individual variability but may exert broader systems-level effects encompassing glucose metabolism, immune regulation, intestinal function, and energy homeostasis.

Overall, polyphenols and probiotics improve insulin sensitivity through complementary mechanisms. Polyphenols directly modulate metabolic, inflammatory, and oxidative stress pathways, producing relatively predictable cellular effects, whereas probiotics act indirectly through gut microbiota remodeling and host–microbial signaling, resulting in broader but more variable metabolic benefits [92,93]. Together, these mechanisms suggest potential synergistic effects on glycemic control by simultaneously targeting host metabolic pathways and microbiome-mediated regulation [92,93].

##### Clinical Evidence

Clinical trials and meta-analyses generally support modest but clinically meaningful improvements in glycemic control following specific nutritional interventions, particularly curcumin supplementation and increased dietary fiber intake. These interventions have been associated with reductions in fasting glucose, HbA1c, insulin resistance indices (e.g., homeostatic model assessment-insulin resistance [HOMA-IR]), and, in some studies, modest improvements in lipid profiles and inflammatory biomarkers [94,95]. Curcumin appears particularly effective in individuals with prediabetes, metabolic syndrome, or type 2 diabetes, where its anti-inflammatory and antioxidant properties may lead to improvements in insulin signaling pathways. Dietary fibers, especially soluble and fermentable fibers such as β-glucans, psyllium, and inulin, improve glycemic regulation through delayed carbohydrate absorption, enhanced satiety, and increased production of microbiota-derived SCFAs, which influence incretin secretion, insulin sensitivity, and hepatic metabolism [94,95].

However, the magnitude of healthy effects varies considerably according to intervention dose, treatment duration, baseline metabolic status, dietary adherence, and formulation characteristics. For example, bioavailability-enhanced curcumin preparations generally demonstrate greater efficacy than conventional formulations, while different fiber types vary in fermentability and metabolic activity [94,95]. Consequently, although improvements in glycemic markers are consistently observed, effect sizes are typically smaller than those achieved with standard pharmacological therapies and are often greatest when nutritional interventions are combined with broader dietary and lifestyle modifications.

In contrast, probiotic interventions demonstrate substantially more heterogeneous outcomes, with glycemic improvements ranging from significant reductions in fasting glucose and HbA1c to negligible or absent effects across studies [92,93,94,95]. This variability is considerably greater than that observed with fiber-based interventions and reflects marked inter-individual differences in gut microbiota composition, metabolic phenotype, dietary background, medication use, and host genetics. Furthermore, efficacy is strongly influenced by strain specificity, with different Lactobacillus and Bifidobacterium species producing distinct metabolic effects, while multi-strain formulations may generate either synergistic or antagonistic interactions [92,93,94,95]. Limited standardization of dosage, treatment duration, viability, and formulation further complicates comparisons among clinical trials and contributes to inconsistent findings.

Emerging evidence suggests that synbiotic approaches combining probiotics with prebiotic fibers may provide greater and more reproducible metabolic health effects than either intervention alone by simultaneously enhancing microbial colonization and substrate availability for SCFA production. Nevertheless, long-term data remain limited, and the durability of these effects beyond the intervention period is not fully established [92,93,94,95].

Conclusively, nutritional and microbiome-targeted interventions can improve metabolic regulation, although their efficacy varies considerably among individuals. Fiber-based interventions and curcumin generally show more consistent clinical advantages than probiotics, while microbiota-targeted approaches may provide broader systems-level effects in selected populations. Current evidence therefore supports their use primarily as adjuncts to established dietary, lifestyle, and pharmacological therapies for type 2 diabetes rather than as stand-alone treatments [92,93,94,95].

#### 5.2.2. Obesity

Obesity is a multifactorial metabolic disorder in which natural compounds generally exert modest but biologically meaningful effects through distinct mechanisms affecting appetite regulation, energy expenditure, adipose tissue biology, inflammation, and gut microbiota composition. Although both microbiota-derived metabolites and dietary bioactive compounds influence energy homeostasis, they differ considerably in their primary targets and metabolic specificity.

##### Mechanistic Comparison

SCFAs represent the most direct microbiota-mediated regulators of body weight because they function as signaling molecules that stimulate enteroendocrine L-cells to release GLP-1 and PYY, thereby enhancing satiety, delaying gastric emptying, and improving postprandial glycemic control [96]. In addition to appetite regulation, SCFAs influence adipose tissue metabolism, hepatic lipogenesis, and energy expenditure through G-protein-coupled receptor signaling and HDAC inhibition, linking dietary fiber fermentation to systemic energy balance [93,97]. However, their efficacy depends strongly on dietary fiber intake, gut microbiota composition, and microbial fermentative capacity, resulting in considerable inter-individual variability [93,97].

In contrast, polyphenols primarily target cellular and metabolic pathways involved in adiposity rather than appetite control. Compounds such as resveratrol, quercetin, and EGCG suppress adipogenesis through inhibition of PPARγ and C/EBPα signaling while enhancing mitochondrial biogenesis and oxidative metabolism via AMPK–silent mating type information regulation 2 homolog 1 (SIRT1)–peroxisome proliferator-activated receptor gamma coactivator 1-alpha (PGC-1α) activation [98]. Compared with SCFAs, polyphenols exert less direct effects on satiety but stronger influences on adipocyte differentiation, lipid oxidation, and thermogenesis. They also promote browning of white adipose tissue and increase energy expenditure; however, these benefits are frequently limited by low bioavailability, extensive metabolism, and dependence on microbial biotransformation into active metabolites [98].

Omega-3 fatty acids occupy an intermediate mechanistic position, acting primarily through immunometabolic rather than appetite- or adipogenesis-related pathways. Their effects are mediated by specialized pro-resolving mediators that reduce adipose tissue inflammation, improve insulin sensitivity, and promote anti-inflammatory macrophage polarization [99]. Unlike SCFAs, which directly regulate energy intake, or polyphenols, which influence adipocyte biology and mitochondrial function, omega-3 fatty acids mainly improve the inflammatory environment associated with obesity. Consequently, they often produce meaningful metabolic improvements despite relatively modest and inconsistent effects on body weight reduction itself [99].

Overall, SCFAs primarily regulate appetite and energy balance, polyphenols mainly influence adipocyte metabolism and thermogenesis, and omega-3 fatty acids exert their greatest effects through reduction of obesity-associated inflammation [96,97,98,99]. Together, these interrelated mechanisms suggest that natural compounds are more effective at improving obesity-related metabolic dysfunction than promoting substantial weight loss, supporting their use as adjuncts to long-term dietary and lifestyle interventions [96,97,98,99].

##### Preclinical Evidence

Preclinical studies of natural products in diet-induced obesity models consistently demonstrate reductions in adiposity, improved lipid profiles, and enhanced metabolic parameters such as insulin sensitivity, glucose tolerance, and inflammatory status; however, both the magnitude and mechanisms of these effects vary substantially among compound classes [100,101]. Polyphenols, fiber-derived metabolites, omega-3 fatty acids, and other plant secondary metabolites frequently produce marked improvements in body weight regulation, adipose tissue remodeling, and energy expenditure under tightly controlled experimental conditions, although these beneficial effects are often greater than those subsequently observed in human studies [100,101].

Mechanistically, different classes of natural compounds target distinct aspects of obesity pathophysiology. Polyphenols such as resveratrol, EGCG, and quercetin primarily suppress adipogenesis through downregulation of PPARγ and C/EBPα signaling while stimulating fatty acid oxidation and mitochondrial biogenesis via AMPK–SIRT1–PGC-1α pathways [100]. These compounds are also among the most potent inducers of white adipose tissue browning and thermogenesis through upregulation of uncoupling protein-1 (UCP1), thereby increasing energy expenditure. In contrast, fiber-derived metabolites, particularly SCFAs, exert less direct effects on adipocyte differentiation but more pronounced influences on satiety signaling, gut hormone secretion (GLP-1 and PYY), hepatic lipid metabolism, and host–microbiome communication through G-protein-coupled receptor activation and epigenetic regulation [101]. Omega-3 fatty acids occupy an intermediate position, acting primarily through anti-inflammatory and immunometabolic pathways that improve adipose tissue function and insulin sensitivity rather than directly promoting weight loss. Collectively, these mechanisms indicate that polyphenols predominantly affect energy expenditure and adipocyte biology, SCFAs regulate energy intake and metabolic signaling, and omega-3 fatty acids mainly attenuate obesity-associated inflammation [100,101].

The observed anti-obesity effects are highly dependent on experimental design variables, including macronutrient composition of the diet (e.g., high-fat versus high-sucrose formulations), duration and timing of intervention, and the specific strain and metabolic phenotype of the animal model used [100,101]. Genetic background strongly influences susceptibility to obesity, inflammation, and insulin resistance, resulting in considerable variability in responsiveness. Furthermore, preventive interventions initiated before substantial weight gain often produce larger effects than treatments administered after obesity is established, suggesting that many natural compounds may be more effective in prevention than reversal of metabolic dysfunction [100].

Baseline gut microbiota structure also plays a critical role in determining treatment efficacy. Polyphenols and dietary fibers exhibit bidirectional interactions with the microbiome, whereby microbial metabolism generates bioactive metabolites while simultaneously reshaping microbial community composition [101]. Consequently, microbiota-dependent interventions often display greater variability than compounds acting directly on host metabolic pathways. Differences in feeding conditions (ad libitum versus caloric restriction), housing environment, physical activity levels, and stress exposure further influence outcomes and may amplify treatment effects compared with those achievable in free-living human populations [100,101].

An important limitation is that many preclinical studies employ doses substantially higher than those achievable through habitual dietary intake, raising concerns regarding physiological relevance and translational feasibility. Moreover, rodents differ from humans in adipose tissue distribution, energy expenditure, feeding behavior, and gut microbiota composition, factors that may alter both efficacy and mechanism of action [100,101]. The relatively short duration of most animal studies further limits their ability to model the chronic, relapsing nature of human obesity. Consequently, while preclinical models provide valuable mechanistic insights and identify promising therapeutic targets, their predictive value for long-term clinical efficacy remains limited, emphasizing the need for carefully designed translational studies that better account for the complexity of human obesity and host–microbiome interactions [100,101].

##### Clinical Evidence

Human intervention studies and meta-analyses indicate that fiber- and polyphenol-rich dietary patterns are associated with small but statistically significant improvements in body composition, including modest reductions in body weight, body mass index (BMI), and waist circumference, alongside improvements in insulin sensitivity, fasting glucose, and lipid profiles [102,103]. Notably, whole-diet interventions generally produce more consistent and durable benefits than isolated nutraceutical supplementation, suggesting that synergistic interactions among multiple dietary components are more effective than single-compound approaches [102,103].

These benefits arise through complementary but distinct mechanisms. Dietary fibers primarily influence appetite regulation, satiety, gastric emptying, and gut microbiota-mediated production of SCFAs, thereby affecting energy intake and metabolic signaling [102,103]. In contrast, polyphenols exert stronger effects on adipocyte metabolism, mitochondrial function, lipid oxidation, inflammation, and oxidative stress, promoting metabolic flexibility rather than directly suppressing appetite. Consequently, fibers appear more effective for regulating energy consumption, whereas polyphenols predominantly improve the metabolic consequences of obesity [102,103].

Despite these favorable effects, the magnitude of weight loss is generally modest and substantially smaller than that achieved with intensive lifestyle interventions, anti-obesity pharmacotherapies, or bariatric surgery. In many studies, improvements in metabolic biomarkers exceed changes in body weight, indicating that these dietary interventions may be more effective at improving metabolic health than inducing substantial fat loss [102,103]. This distinction is particularly important because reductions in insulin resistance, inflammation, and cardiometabolic risk may occur even in the absence of clinically significant weight reduction.

Considerable heterogeneity exists across studies and reflects differences in intervention type, duration, baseline dietary habits, energy intake, physical activity, obesity severity, and metabolic status [102,103]. Individual variation in gut microbiota composition further influences responsiveness, particularly for fiber- and polyphenol-based interventions that depend on microbial metabolism for generation of bioactive metabolites. As a result, some individuals exhibit meaningful improvements in body composition and metabolic markers, whereas others experience minimal benefit under similar interventions [102,103].

Adherence remains a critical determinant of efficacy. Dietary substitution effects, compensatory increases in energy intake, and declining compliance over time frequently attenuate treatment responses. Moreover, the benefits of fiber- and polyphenol-rich diets often diminish following cessation of structured interventions, reflecting physiological adaptations that defend body weight, including changes in appetite-regulating hormones and energy expenditure [102,103]. Thus, long-term success appears to depend more on sustained behavioral adherence than on the intrinsic potency of individual bioactive compounds.

Finally, fiber-rich interventions primarily enhance satiety and reduce energy intake, whereas polyphenol-rich interventions mainly improve metabolic and inflammatory parameters. Whole-diet dietary patterns provide the most consistent favorable effects by targeting diverse pathways simultaneously [102,103]. Thus, their greatest value lies in improving metabolic health and reducing cardiometabolic risk rather than producing substantial long-term weight loss, supporting their role as adjuncts to comprehensive lifestyle-based obesity management [102,103].

### 5.3. Neurodegenerative Diseases

Neurodegenerative diseases represent challenging targets for nutritional and microbiome-based interventions because of their complex, multifactorial pathogenesis involving protein aggregation, neuroinflammation, mitochondrial dysfunction, and metabolic dysregulation [104]. Despite strong mechanistic and preclinical evidence, clinical translation has been limited, largely owing to restricted blood–brain barrier permeability, prolonged preclinical phases, and substantial neuronal loss occurring before diagnosis, which reduces the efficacy of interventions initiated after symptom onset [105].

#### 5.3.1. Alzheimer’s Disease

Alzheimer’s disease (AD) is characterized by a progressive and multifactorial neurodegenerative cascade involving extracellular amyloid-β (Aβ) accumulation, intracellular tau hyperphosphorylation and aggregation, synaptic dysfunction, neurovascular impairment, and sustained neuroinflammatory activation. These processes are tightly interconnected and reflect progressive disruption of neuronal–glial–vascular unit integrity, ultimately resulting in irreversible cognitive decline [106].

##### Mechanistic Considerations

Omega-3 fatty acids, polyphenols, SCFAs, and microbial metabolites influence neurodegenerative disease through distinct but interconnected mechanisms. Omega-3 fatty acids primarily act at the membrane and lipid-signaling level, whereas polyphenols mainly target oxidative stress and inflammatory signaling, and microbiota-derived metabolites regulate neuroimmune and epigenetic pathways [107,108,109,110,111,112].

Omega-3 fatty acids exert neuroprotective effects through incorporation of EPA and DHA into neuronal membranes, improving membrane fluidity, synaptic signaling, and neuronal plasticity [107]. They also serve as precursors for SPMs, which promote resolution of neuroinflammation by shifting microglia toward pro-resolving phenotypes and enhancing clearance of inflammatory debris [108]. Unlike conventional anti-inflammatory agents that primarily inhibit inflammatory initiation, omega-3-derived mediators actively facilitate termination and resolution of inflammatory responses [108].

In contrast, polyphenols such as curcumin, resveratrol, and flavonoids function primarily as modulators of intracellular signaling and redox-sensitive transcriptional networks. Through inhibition of NF-κB and activation of Nrf2 pathways, they reduce oxidative stress, suppress neuroinflammatory signaling, modulate amyloid aggregation, and influence tau phosphorylation [109,110]. Compared with omega-3 fatty acids, polyphenols exert more extensive effects on cellular stress-response pathways but generally face greater translational limitations due to poor bioavailability, rapid metabolism, and limited blood–brain barrier penetration, resulting in variable CNS exposure [110].

SCFAs, including acetate, propionate, and butyrate, represent a distinct class of microbiota-derived neuroactive metabolites. Unlike omega-3 fatty acids and polyphenols, which act primarily through direct effects on neuronal and glial cells, SCFAs regulate neurobiology indirectly through epigenetic and immunometabolic mechanisms. By inhibiting HDACs and activating free fatty acid receptor 2 (FFAR2)/FFAR3 receptors, SCFAs influence gene expression, neuroinflammation, vascular homeostasis, and microglial function [38,111]. However, their efficacy is highly dependent on dietary fiber intake, gut microbial composition, and host metabolic capacity, making their effects more context-dependent than those of directly administered bioactive compounds [38,111].

Microbial tryptophan metabolites constitute another mechanistically distinct pathway linking the gut microbiota to brain health. Through aryl hydrocarbon receptor (AhR) signaling and kynurenine pathway metabolism, these metabolites regulate microglial activation, neurotransmitter balance, and neuroimmune homeostasis [112]. In contrast to the generally neuroprotective effects of SCFAs, dysregulation of tryptophan metabolism can shift production toward neurotoxic intermediates such as quinolinic acid, thereby promoting excitotoxicity, oxidative stress, and synaptic dysfunction [112].

Ultimately, omega-3 fatty acids primarily promote membrane stability and inflammatory resolution, polyphenols regulate oxidative stress and protein aggregation, SCFAs modulate epigenetic and neuroimmune pathways, and microbial tryptophan metabolites influence neurotransmission and microglial activity [107,108,109,110,111,112]. These interrelated actions suggest that combined dietary and microbiome-targeted interventions may provide broader neuroprotective benefits than single-pathway approaches alone.

##### Preclinical–Clinical Gap

Preclinical models of AD consistently demonstrate that omega-3 fatty acids, flavonoids, and other polyphenols exert neuroprotective effects through interrelated but mechanistically distinct pathways [113]. Omega-3 fatty acids, particularly DHA and EPA, primarily target membrane biology, amyloid precursor protein processing, and neuroinflammation. In transgenic AD models, they reduce amyloid burden by promoting non-amyloidogenic α-secretase activity while suppressing β- and γ-secretase pathways, alongside reducing microglial activation and pro-inflammatory cytokine production and enhancing amyloid clearance [114,115]. They also improve mitochondrial function, preserve neuronal membrane integrity, and support synaptic activity [116].

In contrast, flavonoids and polyphenols such as quercetin, EGCG, and curcumin act predominantly through intracellular signaling pathways. These compounds inhibit Aβ aggregation, promote autophagic and lysosomal clearance, and reduce tau hyperphosphorylation through modulation of glycogen synthase kinase-3 beta (GSK-3β) and MAPK signaling [117]. Compared with omega-3 fatty acids, they exert stronger antioxidant effects via activation of the Nrf2–ARE pathway and enhancement of endogenous antioxidant defenses, while also suppressing NF-κB-mediated neuroinflammation in microglia and astrocytes [116,117,118].

Both compound classes improve cognitive performance in behavioral tests such as the Morris water maze, novel object recognition, and Y-maze, although these favorable effects appear to arise through different mechanisms. Omega-3 fatty acids primarily preserve neuronal membrane function and synaptic connectivity, whereas polyphenols more strongly influence cellular stress responses, protein aggregation, and neurotrophic signaling [119,120]. Nevertheless, both enhance synaptic plasticity, increase brain-derived neurotrophic factor (BDNF) expression, support long-term potentiation, and preserve dendritic spine density in hippocampal regions [119,120].

Despite robust preclinical efficacy, translational success remains limited and differs by compound class. Polyphenols are particularly constrained by poor bioavailability, rapid metabolism, and limited brain penetration, whereas omega-3 fatty acids are more readily incorporated into neural tissues but exhibit variability in conversion to specialized pro-resolving mediators and responsiveness across disease stages [121,122]. Additional challenges include differences in blood–brain barrier permeability, timing of intervention, and the inability of animal models to fully replicate the complexity and prolonged progression of human AD [121,122].

Collectively, these findings indicate that omega-3 fatty acids primarily target membrane stability, inflammatory resolution, and mitochondrial function, whereas polyphenols more strongly regulate oxidative stress, protein aggregation, and intracellular signaling pathways [114,115,116,117,118]. Their convergent effects on neuroinflammation, synaptic integrity, and neuronal survival suggest that combination or multi-target approaches may provide greater therapeutic potential than single-compound interventions in AD [113,114,115,116,117,118,119,120,121,122].

#### 5.3.2. Parkinson’s Disease

Parkinson’s disease (PD) is a progressive neurodegenerative disorder characterized by selective degeneration of dopaminergic neurons in the substantia nigra pars compacta, intracellular aggregation of misfolded α-synuclein forming Lewy bodies, mitochondrial dysfunction, impaired proteostasis (including autophagy–lysosomal pathway deficits), and chronic neuroinflammatory activation [123,124]. Although these pathological features are well established, emerging evidence indicates that they represent convergent downstream manifestations of broader upstream disturbances, including metabolic dysfunction, immune dysregulation, and gut–brain axis signaling [123,124].

##### Mechanistic Uncertainty

PD is characterized by interconnected pathological processes involving α-synuclein aggregation, mitochondrial dysfunction, impaired autophagy, lysosomal dysregulation, oxidative stress, and chronic neuroinflammation, all of which contribute to progressive degeneration of dopaminergic neurons in the substantia nigra [125]. Dietary bioactive compounds such as omega-3 fatty acids, flavonoids, SCFAs, and microbial tryptophan metabolites appear to act predominantly on upstream regulatory networks governing inflammation, oxidative stress, mitochondrial function, and immune–metabolic homeostasis rather than directly reversing core neurodegenerative features such as dopaminergic neuronal loss or α-synuclein aggregation [126,127,128,129]. Consequently, these compounds are generally viewed as neuroprotective or disease-modifying adjuncts rather than curative interventions.

SCFAs may modulate microglial activation states and intestinal barrier integrity, thereby attenuating peripheral inflammatory signaling that contributes to CNS pathology through gut–brain axis pathways [128]. By activating FFAR2/FFAR3 signaling and inhibiting HDACs, SCFAs also influence epigenetic programs involved in immune regulation, neuronal survival, and neuroinflammation [128]. Their effects are particularly relevant given growing evidence that gastrointestinal dysfunction and microbiome alterations frequently precede motor symptoms in PD, suggesting a potential role in early disease modulation.

Similarly, polyphenols and flavonoids may enhance mitochondrial resilience and reduce oxidative stress through activation of Nrf2-dependent antioxidant pathways and inhibition of NF-κB-mediated inflammatory signaling [127]. Compounds such as curcumin, quercetin, and EGCG additionally promote autophagic clearance mechanisms, reduce reactive oxygen species generation, and may interfere with α-synuclein oligomerization and fibril formation in experimental models [127]. However, these effects are often limited by poor bioavailability, rapid metabolism, and restricted blood–brain barrier penetration, reducing their translational potential.

Omega-3 fatty acids contribute through distinct but complementary mechanisms. Incorporation of DHA and EPA into neuronal membranes improves membrane fluidity, synaptic signaling, and mitochondrial function while generating specialized pro-resolving mediators that actively suppress neuroinflammation and promote resolution of inflammatory responses [126]. Compared with polyphenols, omega-3 fatty acids may exert stronger effects on membrane stability and inflammatory resolution, whereas polyphenols more strongly influence oxidative stress and intracellular signaling pathways [126,127].

Microbial tryptophan metabolites further contribute to neuroimmune regulation through AhR signaling and kynurenine pathway metabolism [129]. Balanced tryptophan metabolism may support microglial homeostasis and neuroprotection, whereas dysregulation can promote accumulation of neurotoxic intermediates, including quinolinic acid, thereby enhancing excitotoxicity, oxidative stress, and neuronal injury [129].

Despite these promising mechanistic effects, evidence indicates that most dietary bioactive compounds primarily slow or modulate pathogenic processes rather than reverse established neurodegeneration. Their efficacy is likely greatest during prodromal or early disease stages, when neuroinflammatory, metabolic, and gut microbiota abnormalities may still be modifiable. Consequently, current evidence supports their potential role as adjunctive strategies integrated with conventional pharmacological therapies and lifestyle interventions rather than as standalone treatments for established PD [126,127,128,129].

##### Evidence Gap

Preclinical studies consistently demonstrate neuroprotective effects of dietary interventions in toxin-induced and genetic models of PD, including preservation of dopaminergic neurons, improved motor performance, and attenuation of oxidative stress and neuroinflammatory markers [130]. However, different classes of bioactive compounds target distinct aspects of PD pathophysiology. Omega-3 fatty acids primarily support neuronal membrane integrity, mitochondrial function, and resolution of neuroinflammation, whereas polyphenols and flavonoids exert stronger effects on oxidative stress, autophagy, and intracellular signaling pathways involved in neuronal survival [126,127,130]. Microbiota-targeted interventions, in contrast, act more indirectly through modulation of gut permeability, systemic inflammation, SCFA production, and gut–brain axis communication [128,130].

In several experimental models, polyphenols such as curcumin, resveratrol, quercetin, and EGCG reduce α-synuclein aggregation, promote autophagic and lysosomal clearance pathways, and preserve nigrostriatal dopaminergic signaling [126,127,130]. Compared with omega-3 fatty acids, these compounds generally exhibit stronger antioxidant and protein homeostasis effects through activation of Nrf2-dependent pathways and inhibition of NF-κB-mediated neuroinflammation. Omega-3 fatty acids, however, appear more effective at maintaining synaptic function and membrane stability through incorporation of DHA and EPA into neuronal phospholipid membranes and generation of specialized pro-resolving mediators [126,127]. Emerging evidence further suggests that gut microbiota modulation may influence PD progression by altering intestinal permeability, microbial metabolite production, immune activation, and neuroinflammatory signaling, providing a mechanistically distinct but synergetic route of neuroprotection [128,130].

In contrast, human evidence remains predominantly observational, derived largely from dietary pattern analyses and epidemiological cohort studies rather than randomized controlled trials [131,132]. Several prospective studies report that Mediterranean-style dietary patterns and higher intake of polyphenol-rich foods, omega-3 fatty acids, and dietary fiber are associated with lower PD risk, delayed disease onset, or slower symptom progression [131,132]. Notably, dietary patterns generally show more consistent associations than individual supplements, suggesting that synergistic interactions among multiple dietary components may be more important than isolated bioactive compounds. Nevertheless, compared with the robust effects observed in animal models, benefits in human populations are considerably smaller and more variable [131,132].

This discrepancy likely reflects differences in exposure duration, disease stage at intervention, genetic susceptibility, medication use, lifestyle factors, and marked inter-individual variability in gut microbiota composition and metabolic responsiveness [131,132]. Furthermore, animal studies typically evaluate preventive interventions before extensive neuronal degeneration has occurred, whereas human interventions are usually initiated after clinical diagnosis, when substantial dopaminergic neuronal loss may already be irreversible [130,131,132].

Clinical intervention studies have focused primarily on symptomatic outcomes, quality of life measures, and biomarker changes rather than definitive disease-modifying endpoints. Some trials report modest improvements in motor symptoms, gastrointestinal function, inflammatory markers, and oxidative stress following supplementation with omega-3 fatty acids, probiotics, or selected polyphenols, but results remain inconsistent and are often limited by small sample sizes, short follow-up periods, heterogeneous formulations, and inadequate characterization of responders versus non-responders [131,132]. Compared with polyphenols and probiotics, omega-3 fatty acids generally show more consistent biological effects, although evidence for clinically meaningful disease modification remains insufficient.

This persistent translational gap highlights a fundamental distinction between experimental neuroprotection and clinical disease modification. While preclinical studies demonstrate that omega-3 fatty acids, polyphenols, and microbiota-targeted interventions can attenuate multiple pathogenic processes—including neuroinflammation, oxidative stress, mitochondrial dysfunction, α-synuclein pathology, and gut dysbiosis—their ability to reverse established neurodegeneration appears limited [126,127,128,129,130,131,132].

Consequently, future progress may depend on earlier intervention during prodromal disease stages, biomarker-guided patient stratification, microbiome and metabolomic profiling, and multi-target therapeutic strategies that simultaneously address neuroinflammatory, metabolic, microbial, and mitochondrial dysfunction. Such integrated approaches may better reflect the multifactorial nature of PD and offer greater potential for clinically meaningful disease modification than single-pathway interventions alone [126,127,128,129,130,131,132].

### 5.4. Cancer

Cancer is the most mechanistically heterogeneous and adaptive disease category, characterized by genetic, epigenetic, metabolic, and microenvironmental reprogramming that drives clonal evolution, therapeutic resistance, and tumor–stroma co-evolution under continuous selective pressure [133,134]. Unlike neurodegenerative diseases, which are dominated by cellular loss, cancer progression reflects dynamic cellular expansion and adaptation to metabolic, immune, and environmental constraints [133,134].

Within this context, natural compounds function as mechanistically distinct modulators rather than a uniform therapeutic class [135,136]. Omega-3 fatty acids, polyphenols and flavonoids (e.g., curcumin, resveratrol, EGCG), alkaloids such as berberine, and microbiome-derived metabolites including SCFAs and tryptophan derivatives primarily regulate tumor microenvironmental, metabolic, immune, and signaling networks rather than acting as direct cytotoxic agents [135,136].

Mechanistically, polyphenols function as broad signaling modulators with high target promiscuity, omega-3 fatty acids remodel membrane composition and lipid mediator signaling, berberine acts as a multi-target metabolic stressor, and SCFAs and microbial metabolites serve as endogenous immunometabolic and epigenetic regulators integrated into host physiology [137,138,139,140].

Consequently, microbiota-derived metabolites tend to exert more stable systems-level regulation but lower tissue specificity, whereas phytochemicals display greater molecular reactivity but more limited persistence and bioavailability [137,138,139,140].

#### 5.4.1. Anticancer Mechanisms and Mechanistic Limitations

A wide range of dietary bioactive compounds—including curcumin, resveratrol, EGCG, sulforaphane, quercetin, berberine, and omega-3 fatty acids—target major oncogenic pathways such as PI3K/Akt/mammalian target of rapamycin (mTOR), NF-κB, Wnt/β-catenin, MAPK, STAT3, and HDAC-dependent transcriptional regulation [135]. However, because these pathways form highly interconnected and redundant signaling networks, inhibition of a single molecular target is frequently compensated by alternative pathways, limiting the effectiveness of isolated interventions.

Although many natural compounds appear to act on similar signaling pathways, their mechanistic differences become more apparent at the systems level rather than at the level of individual molecular targets [141,142,143]. Polyphenols and flavonoids, including curcumin, EGCG, resveratrol, and quercetin, primarily modulate transcriptional regulators such as NF-κB, Nrf2, and STAT3, producing broad effects on inflammation, oxidative stress, proliferation, and apoptosis. However, their biological activity is often constrained by poor bioavailability, rapid metabolism, and transient tissue exposure, resulting in short-lived signaling perturbations rather than sustained pathway inhibition [141,142,143].

Sulforaphane exhibits a comparatively narrower but more consistent mechanism of action, predominantly activating Nrf2-dependent cytoprotective and detoxification pathways through electrophilic stress signaling. Compared with polyphenols, sulforaphane displays greater mechanistic specificity but influences a smaller range of cellular processes [141,142,143]. Berberine occupies an intermediate position, acting as a multi-target metabolic regulator through AMPK activation, mitochondrial stress induction, and modulation of cellular energy homeostasis. This makes berberine more pharmacologically “drug-like” than many polyphenols, although its clinical utility remains constrained by pharmacokinetic limitations and potential toxicity at higher doses [141,142,143].

Omega-3 fatty acids differ fundamentally from these compounds because they do not primarily target intracellular oncogenic signaling nodes. Instead, they integrate into cellular membranes, altering membrane architecture, lipid raft organization, and eicosanoid signaling, thereby shifting the balance from pro-inflammatory to pro-resolving lipid mediators [141,142,143]. Their effects are therefore more pronounced at the level of the tumor microenvironment and inflammatory regulation than direct suppression of oncogenic pathways.

Microbiota-derived SCFAs, including butyrate, propionate, and acetate, represent the most physiologically integrated class of bioactive compounds. Through HDAC inhibition and FFAR-mediated signaling, they regulate epigenetic programs, immune responses, and cellular metabolism while remaining tightly coupled to host–microbiome interactions [141,142,143]. Compared with exogenous phytochemicals, SCFAs generally exert more stable systems-level regulatory effects but less spatial and molecular specificity.

Although berberine, omega-3 fatty acids, and microbiota-derived SCFAs all exhibit anti-inflammatory and anticancer properties, their mechanisms of action differ substantially. Berberine primarily acts through AMPK activation and regulation of cellular energy metabolism, omega-3 fatty acids modulate membrane signaling and promote the formation of specialized pro-resolving mediators, whereas SCFAs influence host physiology through GPCR-mediated signaling and epigenetic regulation via histone deacetylase inhibition. These mechanistic distinctions likely contribute to their complementary roles in metabolic regulation, inflammation control, and tumor biology.

Despite these mechanistic differences, all classes ultimately converge on a limited set of downstream biological processes, including modulation of reactive oxygen species, autophagy, endoplasmic reticulum stress responses, apoptosis, and epigenetic remodeling [144]. Importantly, although many natural compounds converge on common downstream processes, including oxidative stress regulation, autophagy, and epigenetic remodeling, their biological effects are not determined solely by their primary molecular targets. Cellular phenotype, tissue microenvironment, inflammatory status, metabolic conditions, disease stage, and host–microbiome interactions may substantially influence pathway activation and downstream responses.

Consequently, the biological specificity and therapeutic efficacy of natural compounds are often highly context-dependent, which may contribute to the heterogeneity of findings observed across experimental and clinical studies. Moreover, the limited stand-alone efficacy likely reflects the biological complexity and heterogeneity of cancer, as well as the generally modest magnitude of effect achieved by individual natural compounds compared with established anticancer therapies.

#### 5.4.2. System-Level Constraints in Cancer

The anticancer potential of natural products is limited by key properties of tumor ecosystems [143,144]. Signaling redundancy enables tumor cells to bypass inhibition of pathways such as PI3K/Akt, NF-κB, STAT3, and MAPK through activation of alternative survival networks, reducing the effectiveness of single-target interventions [145]. Intratumoral heterogeneity further complicates responses, as different tumor subclones and stromal components may preferentially respond to omega-3 fatty acids, SCFAs, berberine, or polyphenols, resulting in variable therapeutic sensitivity [146].

The tumor microenvironment (TME) presents an additional barrier by promoting metabolic sequestration, hypoxia-driven reprogramming, extracellular matrix constraints, and immunosuppressive signaling that collectively reduce responsiveness to dietary bioactive compounds [147]. Pharmacokinetic limitations further restrict efficacy, particularly for polyphenols and flavonoids, which often exhibit poor bioavailability, rapid metabolism, and limited tumor penetration, causing strong in vitro effects to translate poorly in vivo [143,144].

In contrast, SCFAs and tryptophan metabolites are integrated components of host–microbiome metabolism and may exert more stable systemic effects through epigenetic, metabolic, and immune regulation, whereas phytochemicals and omega-3 fatty acids function as exogenous agents that must overcome metabolic degradation and tissue distribution barriers [148].

Ultimately, the evolutionary adaptability of cancer and continuous emergence of resistant subclones limit the effectiveness of natural compounds as stand-alone therapies. Their greatest potential likely lies in combination approaches that complement conventional treatments through simultaneous modulation of inflammation, metabolism, immunity, and microbiota–host interactions [143,144,145,146,147,148].

#### 5.4.3. Relative Efficacy Hierarchy and Pharmacological Asymmetry

A key comparative insight is that natural products do not form a uniform efficacy continuum but rather a hierarchy defined by physiological integration, target specificity, potency, and pharmacokinetic behavior. SCFAs and other microbial metabolites exhibit the greatest physiological integration because they are embedded within host–microbiome metabolic networks, providing relatively stable immunometabolic and epigenetic regulation. However, their efficacy is highly dependent on microbiome composition, dietary substrate availability, and host metabolism, resulting in limited controllability and minimal tumor specificity [149].

Omega-3 fatty acids occupy an intermediate position, combining moderate physiological integration with greater systemic stability. Through incorporation into membrane phospholipids and modulation of lipid mediator signaling, they influence inflammation and tumor microenvironment dynamics more consistently than many phytochemicals. Nevertheless, unlike targeted anticancer agents, they lack direct oncogene-specific activity and therefore exert predominantly supportive rather than tumor-selective effects [150].

Alkaloids such as berberine demonstrate comparatively greater intrinsic potency and more direct metabolic effects through AMPK activation, mitochondrial stress induction, and disruption of cellular energy homeostasis [151]. In this respect, berberine is more pharmacologically “drug-like” than most dietary bioactive compounds. However, its translational utility is constrained by toxicity concerns, dose limitations, and unfavorable pharmacokinetic characteristics that restrict sustained therapeutic exposure [151].

In contrast, polyphenols and flavonoids—including curcumin, EGCG, resveratrol, and quercetin—display the broadest target spectrum and highest molecular promiscuity, simultaneously influencing inflammatory, oxidative, epigenetic, and proliferative signaling pathways [152]. While this extensive target engagement generates impressive in vitro activity, it is often accompanied by poor oral bioavailability, rapid metabolic inactivation, and low systemic persistence. Consequently, compounds exhibiting the strongest signaling effects in cell culture frequently show the weakest in vivo durability and tissue exposure [152].

This creates a fundamental translational paradox: the most physiologically integrated compounds (SCFAs and microbial metabolites) provide stable systems-level regulation but lack spatial and oncogene-specific targeting, whereas highly reactive phytochemicals possess broader molecular activity but limited bioavailability and persistence [149,152]. Omega-3 fatty acids and berberine occupy intermediate positions between these extremes, balancing physiological integration with greater direct biological activity, yet still lacking the potency and selectivity required for stand-alone anticancer efficacy [150,151].

Pharmacokinetic constraints—including poor solubility, rapid glucuronidation and sulfation, extensive first-pass metabolism, limited tumor penetration, and heterogeneous intratumoral distribution—further exacerbate this mismatch between biological activity and clinical effectiveness [152]. As a result, achievable tissue concentrations are often substantially lower than those required to reproduce the mechanistic effects observed in experimental systems, particularly for curcumin, resveratrol, and EGCG. Consequently, the translational success of natural products depends not only on intrinsic biological activity but also on their position within the broader hierarchy of physiological integration, target specificity, and pharmacokinetic accessibility [149,150,151,152].

#### 5.4.4. Microbiome-Derived Metabolites: Upstream Regulators with Partial Tumor Embedding

A notable mechanistic divergence arises with microbiome-derived metabolites. Butyrate represents a unique example of metabolic selectivity driven by tumor metabolic reprogramming. Because many cancer cells preferentially utilize glycolysis rather than oxidative metabolism (Warburg effect), butyrate is less efficiently metabolized and accumulates intracellularly, leading to HDAC inhibition, altered gene expression, cell-cycle arrest, and apoptosis preferentially within malignant cells [153,154]. This distinguishes SCFAs from polyphenols, which generally lack intrinsic tumor-selective accumulation and depend largely on systemic exposure, tissue distribution, and concentration-dependent signaling effects [153,154]. Consequently, whereas polyphenols primarily function as exogenous signaling modulators, butyrate exploits a cancer-specific metabolic vulnerability embedded within tumor biology itself.

Additional microbiome-derived metabolites, including secondary bile acids and tryptophan derivatives, further influence carcinogenesis through regulation of AhR signaling, epithelial barrier integrity, immune surveillance, inflammatory tone, and host metabolic homeostasis [155]. Unlike omega-3 fatty acids and polyphenols, which mainly act on downstream inflammatory, oxidative, and proliferative pathways, these metabolites operate upstream by shaping the ecological and immunometabolic environment in which tumor initiation and progression occur. Their effects are therefore less dependent on direct modulation of oncogenic signaling networks and more closely linked to systemic regulation of host–microbiome interactions [155].

This distinction places microbiome-derived metabolites in a different mechanistic category from most dietary phytochemicals. Polyphenols and flavonoids primarily perturb intracellular signaling pathways, omega-3 fatty acids remodel membrane and lipid mediator biology, whereas microbial metabolites influence the broader regulatory landscape governing immune function, metabolism, epithelial homeostasis, and inflammatory responses [153,154,155]. As a result, microbiome-derived metabolites generally exhibit lower molecular specificity but greater systems-level integration and persistence. Their biological effects may therefore be more stable over time, although they remain highly dependent on microbiome composition, dietary substrate availability, and host metabolic context [155].

Collectively, microbial metabolites function as higher-order regulators within the diet–microbiome–host axis. Unlike polyphenols and omega-3 fatty acids, which primarily target downstream signaling pathways, they act upstream by shaping metabolic, immune, and inflammatory networks that influence cancer susceptibility, progression, and therapeutic responsiveness [153,154,155].

#### 5.4.5. Evidence Hierarchy and Translational Decoupling

Preclinical evidence for anticancer activity of dietary bioactive compounds is extensive and includes curcumin, resveratrol, EGCG, sulforaphane, quercetin, and omega-3 fatty acids across multiple tumor types [156]. However, a critical limitation is that these effects are predominantly observed under non-physiological exposure conditions, particularly for polyphenols.

Mechanistically, polyphenols primarily act through the induction of apoptosis, inhibition of kinase signaling pathways, and broad transcriptional reprogramming [157]. Omega-3 fatty acids mainly function by remodeling lipid mediator profiles and attenuating inflammatory signaling [26]. SCFAs exert their effects predominantly through epigenetic regulation and immune–metabolic reprogramming [32]. Alkaloids, in contrast, primarily induce metabolic stress and interfere with mitochondrial function [158]. Despite this diversity, translational failure is driven less by lack of mechanistic relevance and more by insufficient in vivo exposure and tumor delivery efficiency. This limitation is most severe for polyphenols, intermediate for omega-3 fatty acids, and least structurally defined for SCFAs due to endogenous production but high inter-individual variability.

#### 5.4.6. Clinical Interpretation

Clinically, dietary bioactive compounds remain largely confined to adjunctive and supportive roles rather than functioning as primary anticancer therapies [159]. However, important differences exist among compound classes. Omega-3 fatty acids primarily improve systemic inflammation, metabolic health, cachexia-related parameters, and treatment tolerability, whereas polyphenols mainly influence oxidative stress, inflammatory biomarkers, and selected molecular pathways associated with tumor progression [159]. SCFAs and other microbiome-derived metabolites exert broader immunometabolic effects through modulation of host–microbiome interactions, while alkaloids such as berberine display more direct antiproliferative and metabolic effects but with less consistent clinical evidence and greater pharmacokinetic limitations.

Compared with microbiota-derived metabolites, phytochemicals generally exhibit stronger mechanistic activity in experimental systems but weaker clinical persistence due to poor bioavailability and extensive metabolism. Conversely, SCFAs demonstrate greater physiological integration and more stable systemic effects but lack tumor specificity and direct cytotoxic activity. Omega-3 fatty acids occupy an intermediate position, providing relatively consistent anti-inflammatory and supportive benefits without substantial direct effects on tumor burden [159].

Critically, no class of natural products has demonstrated robust and reproducible tumor regression, progression-free survival improvement, or overall survival benefit as a stand-alone intervention across cancer types [159,160]. Reported beneficial effects are typically confined to surrogate biomarkers, treatment-related toxicities, quality-of-life measures, inflammatory parameters, or metabolic outcomes rather than definitive oncologic endpoints. Furthermore, substantial heterogeneity in formulation (free compounds versus nanoencapsulated or enhanced-delivery systems), dosage, treatment duration, patient stratification, tumor subtype, disease stage, microbiome composition, and concurrent therapies continues to obscure true effect sizes and limits cross-study comparability [161]. More to the point, low and variable bioavailability may prevent achievement of therapeutically relevant concentrations, pharmacokinetic differences can alter systemic exposure and target engagement, formulation variability may influence stability and absorption, and patient-specific characteristics such as age, genetics, disease status, microbiome composition, and concomitant therapies can modify treatment responses. Collectively, these factors contribute to heterogeneity in clinical outcomes and complicate direct comparison across studies.

Consequently, the primary clinical value of natural products lies in supplementing conventional therapies through modulation of inflammation, metabolism, immunity, microbiota composition, and treatment tolerance rather than direct antitumor effects. Their benefits are therefore supportive, context-dependent, and influenced by both tumor biology and host-specific factors [159,160,161].

#### 5.4.7. Translational Interpretation

Collectively, the above findings highlight a multi-layered disconnect between mechanistic plausibility and clinical efficacy across major classes of natural products investigated in oncology. Importantly, this translational gap is not uniform but reflects distinct limitations associated with each intervention category. Microbial-derived SCFAs exhibit the greatest physiological integration within host metabolic, immune, and epithelial networks, providing stable regulation of inflammation, barrier integrity, and immune function. However, their therapeutic utility is constrained by limited tumor specificity and strong dependence on host–microbiome interactions [162,163]. In contrast, omega-3 fatty acids display greater systemic stability and more predictable pharmacokinetics, while generating specialized pro-resolving mediators that support inflammation resolution and tissue homeostasis. Nevertheless, their effects remain largely supportive and microenvironment-focused rather than directly cytotoxic or tumor-selective [53].

Plant-derived polyphenols represent the broadest and most mechanistically diverse class, simultaneously influencing oxidative stress, inflammation, apoptosis, angiogenesis, epigenetic regulation, and oncogenic signaling pathways. Compared with SCFAs and omega-3 fatty acids, they exhibit greater molecular target diversity but substantially lower bioavailability and systemic persistence, resulting in tissue concentrations that often fail to reproduce experimental effects observed in vitro [7]. Alkaloids, particularly berberine, occupy an intermediate position by displaying greater pharmacological potency and more direct interference with cancer-related metabolic and signaling pathways. However, unlike polyphenols, whose limitations are predominantly pharmacokinetic, alkaloids are more frequently constrained by toxicity, narrow therapeutic windows, and formulation challenges that restrict clinical applicability [151].

Thus, each class exhibits a distinct balance between physiological integration and biological potency: SCFAs provide the most stable systems-level regulation but the least targeting precision; omega-3 fatty acids offer moderate integration and favorable pharmacokinetics but limited antitumor specificity; polyphenols possess the broadest mechanistic reach but poor in vivo exposure; and alkaloids display the strongest direct biological activity but the greatest safety-related constraints [53,151,162,163]. Consequently, although all four classes possess substantial mechanistic rationale for anticancer activity, none simultaneously combines high potency, tumor specificity, physiological integration, and favorable pharmacokinetics.

Finally, dietary bioactive compounds should not be viewed as a unified therapeutic category but rather as a hierarchically organized spectrum of host–microbiome–tumor modulators operating at different biological levels. Within this framework, cancer represents the disease area in which natural products display their greatest mechanistic breadth but lowest monotherapeutic efficacy, reflecting the dominance of signaling redundancy, tumor heterogeneity, microenvironmental buffering, and evolutionary adaptation. Their most realistic translational role is therefore within multi-modal combination strategies, where they function as modulators of system sensitivity, metabolic state, immune competence, and treatment responsiveness rather than as direct anticancer agents [53,151,162,163].

### 5.5. Gastrointestinal Disorders

Gastrointestinal disorders represent one of the strongest translational settings for dietary bioactive compounds because interventions act directly within the intestinal lumen, achieving high local concentrations and immediate interaction with the microbiota, epithelium, and mucosal immune system [164,165]. However, efficacy varies substantially according to mechanism of action. Polyphenols such as curcumin, resveratrol, EGCG, quercetin, and sulforaphane primarily function as host-signaling modulators, whereas berberine exerts stronger antimicrobial and microbiota-modulating effects but is limited by bioavailability and toxicity concerns. In contrast, dietary fibers and prebiotics act indirectly as microbial substrates, promoting beneficial fermentation and metabolite production rather than exerting direct pharmacological effects.

Compared with systemic diseases, gastrointestinal disorders bypass major pharmacokinetic barriers, allowing dietary compounds, probiotics, and microbial metabolites to act directly at their primary site of action. Nevertheless, rapid chemical degradation, enzymatic metabolism, and microbiome-driven biotransformation create substantial variability in local bioactive exposure [164,165].

Importantly, because the gastrointestinal tract functions as both a metabolic ecosystem and an immune organ, interventions target a dynamic host–microbiome system rather than a single molecular pathway [166]. Consequently, host-directed compounds (e.g., curcumin, resveratrol) tend to be more predictable but less potent, whereas microbiome-dependent interventions (e.g., fibers and prebiotics) often exert broader effects but show greater inter-individual variability [164,165,166].

#### 5.5.1. Mechanistic Robustness

SCFAs, including butyrate, propionate, and acetate, serve as central regulators of intestinal homeostasis by integrating metabolic, epigenetic, and immune signaling pathways. Through HDAC inhibition and activation of FFAR2/FFAR3 receptors, they regulate epithelial differentiation, tight junction assembly, immune tolerance, macrophage polarization, Treg expansion, and inflammation resolution [162,167]. Unlike polyphenols such as curcumin and resveratrol, which act as transient signaling modulators, SCFAs are quantitatively dominant microbial metabolites present at physiologically relevant concentrations, providing more stable and sustained regulatory effects [162,163].

SCFAs also influence mitochondrial metabolism, oxidative phosphorylation, and AMPK signaling, linking microbial fermentation to host energy homeostasis [163]. Butyrate is particularly distinctive because it functions as a primary energy substrate in healthy colonocytes while acting as an epigenetic regulator in inflamed or transformed epithelial cells, thereby coupling metabolic state to transcriptional responses [163]. In contrast, phytochemicals such as curcumin and resveratrol converge on similar pathways—including NF-κB, mitochondrial function, and redox regulation—but generally exhibit lower physiological integration, weaker concentration stability, and greater dependence on bioavailability [163].

Probiotics and next-generation live biotherapeutics reinforce epithelial barrier integrity through enhancement of tight junction proteins, competitive exclusion of pathogens, and production of antimicrobial compounds [168,169]. Compared with SCFAs, however, their effects are more strain-specific and less predictable, being highly dependent on baseline microbiota composition, colonization efficiency, and host immune status [168,169].

Microbial metabolism also generates bioactive indoles, secondary bile acids, and tryptophan metabolites that regulate epithelial regeneration, immune homeostasis, and barrier function through AhR signaling [170,171]. These metabolites differ from dietary phytochemicals such as EGCG, quercetin, and berberine, which may interact with AhR pathways but generally act as secondary modulators rather than primary endogenous ligands. Consequently, microbiota-derived metabolites typically exert more consistent and physiologically integrated signaling than exogenous phytochemicals, whose effects are more variable and exposure-dependent [170,171].

Overall, SCFAs and other microbial metabolites provide the most physiologically integrated and stable regulation of intestinal homeostasis, probiotics offer context-dependent ecological modulation, and phytochemicals act mainly as supplementary signaling modulators. Consequently, these compound classes differ substantially in the magnitude, predictability, and persistence of their effects on intestinal health [162,163,164,165,166,167,168,169,170,171].

#### 5.5.2. Clinical Consistency

Clinical evidence supporting dietary and microbiome-based interventions is strongest in functional and inflammatory gastrointestinal disorders, particularly irritable bowel syndrome (IBS), ulcerative colitis, and, to a lesser extent, Crohn’s disease [172,173,174]. Among available interventions, probiotics, prebiotics, SCFA-modulating dietary strategies, low-FODMAP diets, and polyphenol-rich compounds such as curcumin and green tea catechins have demonstrated beneficial effects in symptom control, barrier function, mucosal healing, and attenuation of inflammatory markers. Notably, dietary pattern–based interventions often produce more consistent clinical benefits than isolated supplements, reflecting the importance of simultaneous modulation of microbial ecology, intestinal metabolism, and immune signaling [172,173,174].

However, important differences exist among intervention classes. Probiotics and dietary fibers exhibit the greatest ecological relevance because they directly influence microbial community structure and metabolite production, yet they also show the highest inter-individual variability and lowest reproducibility. In contrast, polyphenols generally produce more predictable molecular effects through modulation of NF-κB, Nrf2, and epithelial signaling pathways but often yield smaller clinical effect sizes due to poor bioavailability and limited luminal persistence. Alkaloids such as berberine occupy an intermediate position, displaying stronger antimicrobial and microbiota-modulating activity than most polyphenols while retaining more direct host-targeted effects; however, concerns regarding safety, gastrointestinal tolerability, and absorption limit widespread clinical application [172,173,174].

Clinical outcomes remain highly strain-specific and disease-dependent, reflecting substantial heterogeneity in microbiota composition, host immune status, baseline dysbiosis severity, disease phenotype, medication use, and dietary background [172,173,174]. This variability is particularly pronounced for microbiome-dependent interventions, where treatment efficacy depends not only on the administered organism or substrate but also on the capacity of the resident microbiota to metabolize, colonize, and interact with host tissues. Consequently, two individuals receiving the same intervention may generate markedly different metabolic and immunological responses [172,173,174].

A major limitation is the lack of reproducibility across probiotic strains and formulations. Even closely related bacterial species may exhibit divergent effects on epithelial integrity, cytokine production, SCFA generation, bile acid metabolism, and immune regulation. Similarly, the efficacy of dietary fiber interventions depends on fermentability, physicochemical properties, baseline microbiota composition, microbial cross-feeding networks, and the abundance of SCFA-producing taxa. These factors contribute to highly variable responses that are often not adequately accounted for in clinical trials [164,165,172,173,174].

Additional complexity arises from host-specific factors, including bile acid metabolism, epithelial barrier status, mucosal immune tone, genetic susceptibility, and environmental exposures. Variability in these parameters can alter microbial metabolite production, inflammatory responses, and treatment responsiveness, creating multiple layers of biological stratification beyond traditional clinical phenotypes [172,173,174]. Furthermore, many studies are limited by short intervention durations, heterogeneous formulations, inconsistent endpoints, and inadequate microbiome characterization, making direct comparisons across trials difficult.

Despite these limitations, gastrointestinal disorders remain one of the most promising translational domains for natural products because interventions act directly at their primary site of action. Compared with cardiovascular, neurodegenerative, or oncological diseases, the gastrointestinal tract allows dietary compounds, microbial metabolites, probiotics, and prebiotics to interact immediately with epithelial and immune targets. Consequently, although treatment responses remain variable, the mechanistic rationale, local bioavailability, and overall clinical signal are generally stronger than in most other disease categories [164,165,172,173,174].

#### 5.5.3. Translational Interpretation

Collectively, gastrointestinal disorders represent the disease category in which dietary bioactive compounds and microbiome-derived metabolites achieve their highest mechanistic alignment with site of action [175]. However, this alignment is not uniform across natural product classes: SCFAs and microbial metabolites represent high-fidelity endogenous regulators with strong physiological integration; probiotics and fibers represent ecosystem modulators with high context sensitivity; and plant-derived phytochemicals (e.g., curcumin, resveratrol, EGCG, quercetin) represent pleiotropic signaling modifiers constrained by bioavailability and metabolic instability.

SCFAs, probiotics, and microbial metabolites act as integrated regulators of epithelial integrity, immune tolerance, microbial ecology, and metabolic signaling, making the gut a primary translational target for microbiome-centered therapies [176,177]. However, despite comparatively strong clinical responsiveness, therapeutic durability remains variable, emphasizing that long-term efficacy depends on sustained ecological remodeling of the microbiome rather than transient modulation of single microbial or metabolic pathways [178].

This positions gastrointestinal interventions as inherently hierarchical systems-level therapies, where natural products differ not only in potency but in their level of biological organization—ranging from molecular signaling perturbation (polyphenols), to metabolic ecosystem engineering (fibers, probiotics), to endogenous host–microbe regulatory control (SCFAs and microbial metabolites).

### 5.6. Infectious Diseases

Infectious diseases remain a major global health challenge and are caused by a diverse range of pathogens, including bacteria, viruses, fungi, and parasites. The pathogenesis of infectious diseases involves complex interactions between invading microorganisms and the host immune system, often resulting in inflammation, oxidative stress, tissue damage, and disruption of normal physiological functions. In recent years, increasing antimicrobial resistance has highlighted the need for complementary strategies that support host defense mechanisms and enhance the efficacy of conventional therapies [179,180].

Natural products and microbiome-targeted interventions have attracted considerable interest due to their antimicrobial, anti-inflammatory, immunomodulatory, and barrier-protective properties. Bioactive compounds such as polyphenols, flavonoids, alkaloids, and omega-3 fatty acids may interfere with pathogen virulence, modulate immune responses, and promote the resolution of inflammation, while probiotics, prebiotics, and microbial metabolites contribute to colonization resistance and the maintenance of mucosal homeostasis [179,180]. Consequently, these interventions are increasingly being investigated as adjunctive approaches for the prevention and management of infectious diseases.

#### 5.6.1. Mechanistic Distinction

In contrast to chronic disease contexts, infectious disease modulation is governed predominantly by host–microbiome–pathogen interactions, in which immune regulation, epithelial barrier integrity, and ecological competition collectively determine disease trajectory. Within this framework, dietary bioactive compounds and natural compounds function less as classical antimicrobials and more as context-dependent modulators of infection ecology, shaping colonization resistance, immune calibration, and pathogen fitness within the host niche [181].

Importantly, these interventions act at diverse hierarchical levels. At the epithelial interface, they reinforce barrier integrity through modulation of mucin production, tight junction assembly, and antimicrobial peptide expression (e.g., defensins, cathelicidins). At the immune level, they tune pattern recognition receptor signaling, including Toll-like receptor (TLR) and nucleotide-binding oligomerization domain (NOD)-like pathways, thereby influencing both tolerance and inflammatory activation thresholds. At the ecological level, microbiota-derived metabolites—particularly SCFAs and indole derivatives—further reinforce colonization resistance through pH modulation, epithelial fortification, and immune conditioning [170,171,176].

A critical distinction among natural product classes is their level of biological leverage: some act primarily at the microbial virulence interface, others at host inflammatory resolution pathways, and others at ecosystem-level metabolic structuring [178,179,180].

#### 5.6.2. Polyphenols and Flavonoids: Virulence Attenuation with Limited Systemic Antimicrobial Impact

Polyphenols represent the most extensively studied class of plant-derived anti-infective agents; however, their activity is predominantly anti-virulence rather than bactericidal in vivo. Compounds such as curcumin, resveratrol, EGCG, quercetin, berberine, and allicin act primarily by disrupting quorum sensing, inhibiting adhesion factors, and impairing biofilm maturation. Among these, EGCG and berberine demonstrate relatively broader antimicrobial spectra, whereas curcumin and resveratrol are more accurately characterized as host-response modulators with indirect antimicrobial effects due to limited bioavailability [182,183].

From a comparative perspective, flavonoids and tannins often exert enzymatic inhibition and membrane perturbation effects, but these interactions are typically reversible and concentration-dependent, limiting their translational antimicrobial potency [184,185]. In contrast, alkaloids such as berberine exhibit stronger multi-target activity, including interference with nucleic acid synthesis and efflux pump modulation; however, their clinical efficacy remains constrained by pharmacokinetic limitations and systemic toxicity thresholds [186].

Overall, polyphenols occupy an intermediate position: high mechanistic diversity but low in vivo antimicrobial reliability, making them more relevant as adjunctive anti-virulence agents than direct anti-infectives.

#### 5.6.3. Omega-3 Fatty Acids and Specialized Pro-Resolving Mediators (SPMs): Immune Resolution Rather than Antimicrobial Action

Omega-3–derived SPMs, including resolvins, protectins, and maresins, fundamentally differ from polyphenols in that they do not target pathogens or virulence factors. Instead, they regulate the temporal dynamics of inflammation, actively promoting resolution rather than suppression [42].

Their mechanisms include enhancement of efferocytosis, downregulation of neutrophil recruitment, and restoration of endothelial and epithelial homeostasis. This positions SPMs as disease-phase regulators rather than antimicrobial agents. Compared to polyphenols, which act at the host–pathogen interface, SPMs operate primarily at the host inflammatory resolution axis, making them more relevant for preventing tissue damage than controlling microbial burden [187].

Consequently, omega-3-derived mediators represent a functionally orthogonal strategy: they do not reduce pathogen load directly but instead mitigate collateral host injury and accelerate return to homeostasis.

#### 5.6.4. Microbiome-Targeted Interventions: Ecosystem-Level Competition and Metabolic Reinforcement

Probiotics and next-generation live biotherapeutics occupy a distinct mechanistic niche characterized by ecological rather than molecular targeting. Species such as Lactobacillus and Bifidobacterium exert effects through competitive exclusion, nutrient niche occupation, bacteriocin production, and modulation of luminal physicochemical conditions (e.g., pH reduction via lactic acid production) [174,176,188].

Importantly, their efficacy is highly strain-specific and context-dependent, with functional outcomes varying according to baseline microbiome composition and host immune tone. This distinguishes them from polyphenols and omega-3 fatty acids, which are chemically defined but biologically context-dependent, whereas probiotics are biologically active entities whose effects are intrinsically ecosystem-dependent [174,176,188].

Prebiotic fibers (inulin, resistant starch, fructo-oligosaccharides) further extend this ecological modulation by selectively enhancing SCFAs-producing taxa. However, this effect is indirect, contingent on microbial functional capacity, and therefore highly variable across individuals. In this sense, fiber-based interventions represent the most ecosystem-sensitive and least predictable of the natural product classes discussed.

#### 5.6.5. Clinical Limitations

Clinical evidence supports predominantly preventive and adjunctive roles for natural products and microbiome-targeted interventions, particularly in antibiotic-associated diarrhea, recurrent Clostridioides difficile infection, and selected upper respiratory tract infections. However, comparative efficacy varies substantially across compound classes [173,176].

Probiotics and synbiotics demonstrate the most consistent clinical signal, but effects are generally modest and highly strain-specific [164]. Polyphenols and flavonoids show stronger efficacy signals in preclinical models than in human infection outcomes, reflecting a large translational gap driven by pharmacokinetic constraints and insufficient in vivo exposure [189]. Alkaloids such as berberine exhibit comparatively stronger antimicrobial potential but are limited by safety margins and systemic distribution challenges [190].

Omega-3 fatty acids and SPMs precursors show the least direct anti-infective effects but the most consistent benefits in terms of inflammation resolution and tissue protection, underscoring their role as supportive rather than antimicrobial agents [191].

Across all classes, a central limitation is the disconnect between potent in vitro activity and weak in vivo efficacy, largely driven by metabolism, poor bioavailability, and insufficient site-specific accumulation. Even highly active compounds such as curcumin, EGCG, and berberine require advanced formulation strategies (nanoencapsulation, phospholipid complexes, or liposomal delivery systems) to approach biologically relevant concentrations in clinical settings.

#### 5.6.6. Integrated Interpretation

Recent evidence supports a hierarchical functional stratification of natural products in infectious disease, reflecting distinct layers of host-centered therapeutic action. Polyphenols and flavonoids primarily act as anti-virulence and signaling modulators, exerting broad mechanistic effects on quorum sensing, biofilm formation, and host–pathogen interactions but demonstrating limited clinical potency due to pharmacokinetic constraints [157,189]. Alkaloids such as berberine occupy an intermediate position as multiple targeting antimicrobial-adjacent agents, exhibiting strong in vitro antibacterial activity but constrained by poor bioavailability and toxicity-related dosing limitations [190]. In contrast, omega-3–derived specialized pro-resolving mediators function as non-antimicrobial inflammation-resolution regulators, enhancing efferocytosis and restoring tissue homeostasis rather than directly affecting pathogen burden [191]. Probiotics and prebiotics act as ecological competitors and metabolic modulators, with strain-specific and context-dependent efficacy driven by host microbiome composition [175,176]. Finally, SCFAs and other microbial metabolites function as endogenous host–microbe signaling integrators, linking microbial metabolism to epithelial barrier function and immune calibration [177,181].

This stratification highlights that infectious disease modulation by natural compounds is inherently non-uniform and mechanistically layered, with therapeutic outcomes emerging from the interplay between microbial ecology, host immunity, and compound-specific pharmacological constraints rather than direct pathogen eradication.

### 5.7. Conclusion Remarks

Overall, dietary bioactive compounds demonstrate uneven translational efficacy across disease domains. Cardiometabolic and gastrointestinal disorders show the strongest alignment between mechanistic understanding and clinical outcomes, largely due to accessible target tissues and measurable intermediate biomarkers. In contrast, neurodegenerative diseases and cancer exhibit substantial preclinical promise but limited clinical translation, primarily due to pharmacokinetic barriers, disease complexity, and late-stage intervention timing.

Collectively, these findings support a refined paradigm of systems nutrition pharmacology, in which dietary bioactive compounds function as low-potency, high-network-coverage modulators rather than classical pharmacological agents. Their greatest therapeutic potential likely lies not in monotherapy, but in context-dependent, multi-component dietary strategies integrated with conventional medical treatments.

## 6. Critical Interpretation of Clinical Evidence and Efficacy Across Human Disease Domains

### 6.1. Critical Summary of Human Clinical Evidence

Human clinical evidence for natural products and microbiome-targeted interventions is derived primarily from RCTs, systematic reviews, and meta-analyses evaluating intermediate cardiometabolic endpoints, inflammatory biomarkers, and, in selected cases, hard clinical outcomes. Across disease domains, the overall evidence base demonstrates a consistent pattern: biologically plausible and mechanistically supported effects are most robust at the level of surrogate markers, while translation into clinically meaningful reductions in morbidity and mortality is more limited and intervention-specific [192,193]. In Table 3, the main human clinical outcomes of natural compounds and the key limitations in each disease category are summarized.

In cardiovascular disease, the most extensively studied interventions include omega-3 fatty acids, red yeast rice, plant sterols, dietary fiber, cocoa flavanols, nitrate-rich vegetables, and dietary patterns such as the Mediterranean diet. Meta-analyses consistently show that omega-3 fatty acids reduce triglycerides and may modestly reduce major adverse cardiovascular events (MACE) in high-risk populations, particularly with purified EPA formulations, whereas mixed EPA/DHA preparations yield more heterogeneous outcomes [194]. Red yeast rice preparations demonstrate LDL-C reductions comparable to low-intensity statin therapy and may improve other lipid parameters; however, variability in monacolin K content, product quality, and regulatory standardization limits reproducibility and clinical implementation [73,195]. Plant sterols and soluble fibers such as β-glucans and psyllium consistently reduce LDL-C in a dose-dependent manner through inhibition of cholesterol absorption and enhanced bile acid excretion, while cocoa flavanols improve endothelial function and vascular reactivity, and dietary nitrates lower blood pressure through nitric oxide-mediated mechanisms [67,196]. Additional evidence supports modest cardiometabolic favorable effects of garlic, CoQ10, and selected polyphenols, although effects are generally smaller and less consistent than those observed with established pharmacological therapies [197,198,199]. Among dietary interventions, the Mediterranean diet remains the most consistently supported strategy for reducing cardiovascular events and cardiovascular mortality, as demonstrated in large-scale RCTs such as PREDIMED and corroborated by numerous prospective cohort studies and meta-analyses [200].

In metabolic disorders, including T2DM and obesity, clinical trials and meta-analyses indicate modest but statistically significant improvements in glycemic control, insulin sensitivity, lipid metabolism, and body composition following supplementation with curcumin, dietary fiber, and selected polyphenols. Berberine demonstrates broad metabolic effects, including reductions in fasting glucose, HbA1c, LDL-C, triglycerides, and insulin resistance indices, with efficacy in some studies approaching that of first-line metabolic therapies; however, substantial heterogeneity in study design, dosage, formulation, and treatment duration limits direct comparability across trials [201]. Fiber-based interventions improve satiety, postprandial glycemic responses, and gut microbiota composition, whereas polyphenols may exert metabolic effects through AMPK activation, modulation of oxidative stress, and attenuation of chronic low-grade inflammation. In obesity, fiber- and polyphenol-rich interventions yield small reductions in body weight, BMI, body fat, and waist circumference, but sustained long-term efficacy remains limited, with benefits frequently attenuating after intervention cessation and in the absence of broader lifestyle modification [202,203]. Probiotic interventions show substantial variability in both diabetes and obesity outcomes, reflecting strain-specific effects, formulation differences, treatment duration, and marked interindividual variation in baseline microbiome composition and host metabolic phenotype [204,205]. Consequently, although microbiome-targeted therapies remain promising, their clinical application currently lacks the consistency required for universal recommendations.

In neurodegenerative diseases, particularly AD and PD, clinical evidence remains considerably weaker than the extensive mechanistic and preclinical literature. Omega-3 fatty acids, flavonoids, curcumin, resveratrol, and other polyphenols have demonstrated favorable effects on inflammatory, oxidative stress, and neurotrophic biomarkers, and some studies report modest improvements in cognitive performance or disease-related symptoms [194,199,206,207]. However, RCTs have not consistently demonstrated clinically meaningful effects on cognitive decline, disease progression, or long-term functional outcomes. This translational gap likely reflects limited bioavailability, poor blood–brain barrier penetration, late intervention after substantial neurodegeneration has occurred, and the prolonged latency of neurodegenerative diseases. Thus, current evidence supports a potential preventive or adjunctive role for selected natural compounds, but not disease-modifying efficacy.

In oncology, evidence for natural products remains predominantly adjunctive rather than curative. Omega-3 fatty acids, polyphenols, dietary fiber, probiotics, and microbiome-derived metabolites such as SCFAs primarily improve systemic inflammation, metabolic status, treatment tolerance, gastrointestinal symptoms, and quality-of-life outcomes rather than directly reducing tumor burden or improving overall survival [208,209,210,211]. Some interventions have demonstrated benefits in mitigating cancer-related cachexia, chemotherapy-induced toxicity, and treatment-associated inflammation. Nevertheless, no nutraceutical class has consistently demonstrated reproducible tumor regression, progression-free survival benefit, or overall survival improvement as monotherapy in high-quality randomized clinical trials [208,209,210,211]. Their therapeutic role therefore appears largely supportive, context-dependent, and most effective when integrated with conventional oncologic therapies.

In gastrointestinal disorders, clinical evidence is comparatively stronger because bioactive compounds and microbiome-targeted interventions act directly within the intestinal lumen and exhibit relatively high local bioavailability. Probiotics, dietary fiber, and selected polyphenols such as curcumin demonstrate consistent benefits in functional gastrointestinal disorders, particularly IBS, as well as inflammatory bowel diseases including ulcerative colitis [212,213,214,215,216]. Reported benefits include improvements in symptom severity, intestinal barrier integrity, inflammatory biomarkers, and maintenance of remission in selected patient populations. However, clinical responses remain highly strain-specific and depend on formulation characteristics, microbiome composition, disease subtype, and baseline disease activity [212,213,214,215,216]. Consequently, although efficacy is generally more robust than in several other disease domains, substantial heterogeneity remains across studies.

In infectious diseases, probiotics and synbiotics provide the most consistent, albeit modest, clinical benefits, particularly in the prevention of antibiotic-associated diarrhea, reduction of gastrointestinal infections, and prevention of recurrent Clostridioides difficile infection [217,218,219,220]. Certain probiotic strains also demonstrate beneficial effects on respiratory tract infections, although results remain variable across populations and clinical settings [221,222]. Polyphenols and flavonoids exhibit broad-spectrum antimicrobial, anti-virulence, and immunomodulatory properties in experimental models, but these effects have translated less successfully into human clinical trials because of bioavailability and pharmacokinetic limitations [182,223,224]. Alkaloids such as berberine occupy an intermediate position, demonstrating antimicrobial, anti-inflammatory, and microbiome-modulating activities, yet clinical implementation remains constrained by formulation challenges, safety considerations, and limited high-quality trial data [190,225,226]. Omega-3 fatty acids and SPMs primarily support host defense through enhancement of inflammation resolution and tissue recovery rather than direct antimicrobial activity [53,227]. Overall, current evidence suggests that natural products and microbiome-targeted interventions are most effective as adjunctive strategies that support host resilience, immune regulation, and recovery rather than as stand-alone antimicrobial therapies.

### 6.2. Evaluating the Strength of Clinical Evidence Across Disease Areas

Across disease domains, the strength of clinical evidence follows a clear hierarchical pattern. The strongest and most clinically validated evidence is observed in CVD prevention, particularly for Mediterranean dietary patterns, high-dose EPA-based omega-3 formulations, and LDL-lowering interventions such as plant sterols and red yeast rice. These interventions demonstrate consistent effects on validated surrogate biomarkers and, in select cases, reductions in hard cardiovascular outcomes.

A moderate level of evidence is observed in metabolic disorders and gastrointestinal diseases. In these categories, interventions such as dietary fiber, polyphenols, probiotics, and berberine consistently improve intermediate metabolic endpoints (e.g., HbA1c, fasting glucose, body weight, inflammatory markers, and gastrointestinal symptom scores). However, the magnitude of effect is generally modest, and long-term durability is uncertain.

A weaker and largely inconsistent evidence base is observed in neurodegenerative diseases, oncology, and infectious diseases. In these domains, most natural products demonstrate strong mechanistic plausibility and robust preclinical effects but limited or inconsistent translation into clinically meaningful outcomes. The exception is gastrointestinal disease, where local exposure conditions partially overcome systemic pharmacokinetic constraints. Overall, the evidence hierarchy can be conceptualized as: CVDs (strongest) > metabolic/gastrointestinal (moderate) > neurodegenerative/cancer/infectious (weak to emerging).

More to the point, the relative clinical effectiveness of natural products varies substantially across disease categories. Cardiovascular and metabolic disorders currently demonstrate the most consistent clinical evidence, particularly for interventions such as omega-3 fatty acids, plant sterols, dietary fiber, selected probiotics, and certain polyphenols, which target well-defined risk factors including dyslipidemia, insulin resistance, inflammation, and metabolic dysfunction. In contrast, evidence in neurodegenerative diseases remains more limited because of the multifactorial nature of these disorders, challenges in achieving adequate central nervous system bioavailability, and the long disease course required to detect clinically meaningful outcomes. Similarly, in oncology, although numerous natural compounds exhibit promising mechanistic and preclinical anticancer activities, clinical benefits have generally been confined to supportive care, symptom management, or modulation of treatment-related toxicities rather than direct effects on tumor progression or survival. These differences likely reflect variations in disease complexity, target accessibility, intervention timing, pharmacokinetic limitations, and the availability of validated clinical endpoints.

### 6.3. Variability in Clinical Outcomes (Dose, Formulation, and Population Differences)

A defining characteristic across all disease areas is the high heterogeneity in clinical outcomes, driven by multiple interacting factors. First, dose dependency is a major determinant of efficacy. Omega-3 fatty acids, for example, show limited or inconsistent effects at low doses, whereas high-dose purified EPA demonstrates clearer cardiovascular benefit. Similarly, dietary nitrate and cocoa flavanol interventions exhibit dose-dependent improvements in blood pressure and endothelial function, respectively.

Second, formulation and standardization issues substantially influence reproducibility. Red yeast rice preparations vary widely in monacolin K content, leading to inconsistent lipid-lowering effects across studies. Polyphenols such as curcumin, resveratrol, and EGCG exhibit poor bioavailability and rapid metabolic degradation, resulting in discrepancies between in vitro efficacy and in vivo clinical outcomes. Advanced delivery systems (e.g., nanoencapsulation, lipid carriers) partially mitigate these limitations but are not consistently applied across trials.

Third, population heterogeneity significantly modulates response. Baseline metabolic status, disease severity, genetic predisposition, and gut microbiome composition strongly influence responsiveness to fiber, probiotics, and polyphenol-based interventions. For instance, probiotic efficacy in metabolic and gastrointestinal disorders is highly strain-specific and host-dependent, while omega-3 responsiveness varies with baseline triglyceride levels and inflammatory status.

Fourth, study design variability (trial duration, sample size, endpoint selection, and adherence monitoring) contributes to inconsistency across meta-analyses. Short-term trials may capture biomarker changes without reflecting long-term clinical outcomes, while longer studies often show attenuation of initial effects due to adherence decline and compensatory dietary behaviors.

### 6.4. Comparison with Conventional Clinical Therapies

When compared with conventional pharmacological and lifestyle-based therapies, natural products and microbiome-targeted interventions generally demonstrate complementary rather than equivalent therapeutic efficacy.

In cardiovascular disease, statins and antihypertensive drugs remain substantially more effective in reducing LDL-C, blood pressure, and cardiovascular events. However, nutraceuticals such as plant sterols, dietary fiber, and omega-3 fatty acids provide adjunctive benefits, particularly in individuals with residual risk or statin intolerance. The Mediterranean diet represents one of the few dietary interventions with evidence approaching pharmacological impact on cardiovascular outcomes, although its effect size remains lower than that of combined drug therapy.

In metabolic diseases, pharmacological agents such as metformin, GLP-1 receptor agonists, and sodium-glucose cotransporter 2 (SGLT2) inhibitors demonstrate superior glycemic control and weight reduction compared to nutraceuticals. However, fiber, polyphenols, and berberine may provide supportive metabolic regulation, particularly through gut microbiota modulation and inflammation reduction.

In neurodegenerative diseases and oncology, conventional therapies remain dominant, with disease-modifying drugs and chemotherapeutic regimens showing significantly greater clinical efficacy than nutraceutical interventions. Natural products in these contexts are best interpreted as adjunctive agents that modulate inflammation, oxidative stress, and treatment tolerance, rather than primary disease-modifying therapies.

In gastrointestinal disorders, the gap between conventional and natural interventions is narrower. Probiotics, dietary modifications, and fiber-based therapies can achieve clinically meaningful symptom improvement in functional disorders such as IBS and may serve as first-line or co-primary interventions alongside pharmacological treatments.

In infectious diseases, antibiotics remain the gold standard for pathogen eradication, whereas probiotics, prebiotics, and certain phytochemicals function primarily as supportive agents that modulate host immunity, microbiota resilience, and inflammation resolution, rather than direct antimicrobial substitutes.

Consequently, the clinical evidence supports a unified interpretation: natural products and microbiome-targeted interventions occupy a graded therapeutic continuum, ranging from strong evidence-based adjuncts in cardiovascular prevention to mechanistically promising but clinically limited agents in neurodegenerative disease, oncology, and infectious disease. Their principal clinical value lies not in replacement of conventional therapies, but in multi-target, systems-level modulation of metabolic, inflammatory, and microbiome-driven pathways that complement established medical treatment strategies.

Overall, despite encouraging mechanistic and clinical findings, natural products and microbiome-targeted interventions generally function as adjunctive rather than stand-alone therapies. Compared with conventional pharmacological agents, their effects are often characterized by lower target specificity, smaller effect sizes, variable bioavailability, substantial interindividual variability, and dependence on dietary, metabolic, and microbiome-related factors. Consequently, while these interventions may complement standard treatments by improving metabolic, inflammatory, or quality-of-life outcomes, they have rarely demonstrated efficacy comparable to established pharmacotherapies for major diseases.

## 7. Limitations and Challenges

**Figure 4:**
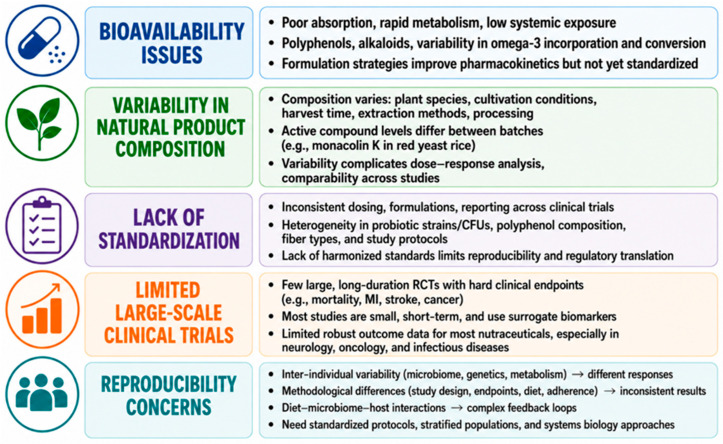
Principal limitations and translational challenges affecting the clinical application of natural products across major disease categories.

Despite the expanding body of clinical and preclinical evidence supporting the potential role of natural products and microbiome-targeted interventions across cardiovascular, metabolic, neurodegenerative, oncological, gastrointestinal, and infectious diseases, several fundamental limitations constrain their translational impact. These challenges collectively explain the persistent gap between strong mechanistic plausibility and inconsistent clinical efficacy observed across multiple disease domains. The most important limitations and challenges of natural products across cardiovascular, metabolic, neurodegenerative, oncological, gastrointestinal, and infectious diseases are depicted in Figure 4.

### 7.1. Bioavailability Issues

A primary limitation across nearly all classes of natural bioactive compounds is poor systemic bioavailability, which significantly restricts their clinical effectiveness despite strong in vitro and preclinical activity. Polyphenols such as curcumin, resveratrol, EGCG, quercetin, and related flavonoids exhibit rapid metabolism via glucuronidation, sulfation, and first-pass hepatic clearance, resulting in extremely low circulating concentrations after oral administration. Consequently, their observed biological activity in vivo is often decoupled from the concentrations required to reproduce mechanistic effects identified in experimental systems [157,228].

Similarly, alkaloids such as berberine demonstrate poor oral absorption and limited tissue distribution, despite showing broad metabolic and antimicrobial effects in experimental models [229]. Even nutritionally relevant compounds such as omega-3 fatty acids are subject to variability in incorporation efficiency into cellular membranes and downstream conversion into bioactive lipid mediators, including SPMs, which is influenced by enzymatic capacity and host metabolic status [230].

Emerging formulation strategies—including nanoencapsulation, phospholipid complexes, emulsions, and liposomal delivery systems—have improved pharmacokinetic profiles for selected compounds; however, these approaches are not yet standardized across clinical research, contributing to heterogeneity in observed outcomes.

### 7.2. Variability in Natural Products Composition

A second major challenge is the intrinsic variability in chemical composition of natural products, which arises from differences in plant species, cultivation conditions, harvesting time, extraction methods, and post-processing procedures. Unlike synthetic pharmaceuticals, which are structurally defined and chemically uniform, many nutraceuticals and herbal products contain complex mixtures of bioactive and inactive constituents whose concentrations can vary substantially between batches.

A clear example is red yeast rice, in which monacolin K content—the compound responsible for HMG-CoA reductase inhibition—can vary widely across commercial preparations, leading to inconsistent lipid-lowering efficacy [231]. Similarly, botanical extracts such as garlic, Ginkgo biloba, and polyphenol-rich formulations (e.g., green tea extracts, curcumin preparations) differ significantly in active compound concentration, stability, and isomer composition [232,233,234]. This variability complicates dose–response interpretation and limits the ability to establish reliable pharmacological equivalence across studies, thereby reducing the interpretability of meta-analytic findings.

### 7.3. Lack of Standardization

Closely related to compositional variability is the lack of standardized dosing, formulation, and reporting frameworks across clinical trials. Many studies fail to clearly define active compound concentration, bioequivalent dosing, or bioavailability-adjusted exposure, making cross-study comparison difficult.

In probiotic research, for example, heterogeneity in bacterial strains, colony-forming unit (CFU) counts, delivery matrices, and treatment durations leads to highly inconsistent outcomes even within the same clinical indication. Strain-specific effects further complicate interpretation, as closely related microbial species can exhibit markedly different immunomodulatory and metabolic profiles [235,236].

In polyphenol and fiber interventions, variability in chemical composition (e.g., fermentability of fibers, polyphenol glycosylation patterns) and inconsistent dietary background conditions introduce additional confounding factors [84,237]. Across all categories, the absence of harmonized reporting standards limits reproducibility and impedes regulatory translation.

### 7.4. Limited Large-Scale Clinical Trials

Although the number of RCTs evaluating natural products has increased substantially in recent years, there remains a relative scarcity of large-scale, long-duration clinical trials powered for hard clinical endpoints such as mortality, myocardial infarction, stroke, cancer progression, or neurodegeneration. Most existing studies are characterized by small sample sizes, short intervention durations, and surrogate endpoints such as lipid levels, inflammatory markers (e.g., CRP), glycemic indices, or endothelial function (e.g., flow-mediated dilation). While these biomarkers provide mechanistic insight, they do not always translate into clinically meaningful outcomes [193,238,239].

The strongest evidence for hard endpoints remains limited to a small subset of interventions, notably Mediterranean dietary patterns and high-dose EPA formulations in cardiovascular disease. In contrast, most nutraceuticals—including polyphenols, probiotics, curcumin, and resveratrol—lack robust long-term outcome data, particularly in neurodegenerative diseases, oncology, and infectious diseases. Hence, the absence of large, multicenter, standardized trials limits confidence in generalizability and restricts integration into evidence-based clinical guidelines.

### 7.5. Reproducibility Concerns

A pervasive challenge across the entire field is low reproducibility of clinical findings, driven by a combination of biological, methodological, and ecological factors. Biologically, inter-individual variability in gut microbiome composition, genetic background, metabolic state, and immune responsiveness leads to highly divergent responses to identical interventions. This is particularly evident in probiotic, fiber, and polyphenol studies, where baseline microbial ecology strongly determines metabolic outcomes such as SCFAs production or bile acid transformation [237,240].

Methodologically, variability in study design—including differences in endpoints, statistical thresholds, dietary control, and adherence monitoring—contributes to inconsistent replication of results across independent trials [241,242]. Short-term studies may capture transient biomarker improvements that are not sustained in longer follow-up periods, further reducing reproducibility. Ecologically, diet–microbiome–host interactions introduce dynamic feedback loops that are difficult to control experimentally, particularly in free-living human populations. As a result, interventions that appear effective under controlled conditions may yield attenuated or inconsistent effects in real-world settings.

Collectively, these reproducibility challenges highlight the need for more standardized protocols, stratified patient selection, microbiome-informed trial design, and systems biology approaches to better capture inter-individual variability.

### 7.6. Overall Perspective

Taken together, these limitations emphasize that the clinical translation of natural products and microbiome-based interventions is constrained not by lack of biological activity, but by pharmacokinetic instability, compositional heterogeneity, insufficient standardization, limited large-scale evidence, and high inter-individual variability. Addressing these challenges will require integration of advanced formulation technologies, standardized extraction and reporting frameworks, and precision medicine approaches that account for host–microbiome interactions.

## 8. Safety Considerations

**Figure 5:**
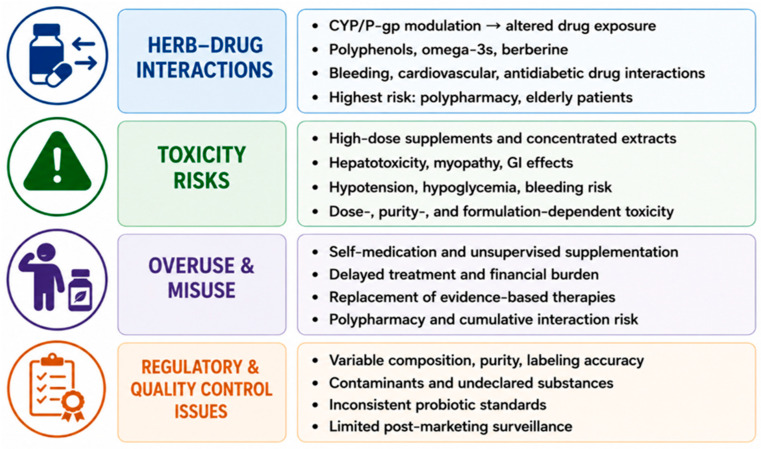
Overview of the principal safety considerations associated with natural products.

Although natural products and microbiome-targeted interventions are often perceived as inherently safe due to their dietary origin, clinical and pharmacological evidence indicates that their safety profile is complex and context-dependent. Across cardiovascular, metabolic, neurodegenerative, gastrointestinal, infectious, and oncological applications, safety concerns arise not only from intrinsic compound toxicity, but also from variability in dosing, formulation quality, and interactions with conventional pharmacotherapies. These issues are particularly relevant given the widespread use of over-the-counter nutraceuticals and the frequent lack of medical supervision in their consumption. The most important safety considerations of the natural products are summarized in Figure 5.

### 8.1. Herb–Drug Interactions

A major safety concern is the potential for clinically significant herb–drug interactions, particularly in patients receiving chronic pharmacotherapy for cardiovascular, metabolic, or oncological conditions. Many natural compounds influence key metabolic enzymes and transport systems, including cytochrome P450 isoenzymes (CYP3A4, CYP2D6), P-glycoprotein transporters, and phase II conjugation pathways, thereby altering the pharmacokinetics of co-administered drugs.

For example, polyphenols such as curcumin, quercetin, and resveratrol can modulate drug-metabolizing enzymes and may alter systemic exposure to anticoagulants, antiplatelet agents, statins, and antihypertensive medications [243]. Omega-3 fatty acids, while generally well tolerated, may potentiate bleeding risk when combined with anticoagulants or antithrombotic agents in high doses [244]. Similarly, berberine has been reported to influence CYP-mediated metabolism and P-glycoprotein activity, potentially affecting plasma levels of antidiabetic and cardiovascular drugs [245].

In oncology, the concurrent use of antioxidant supplements during chemotherapy or radiotherapy remains controversial, as theoretical concerns exist regarding interference with oxidative stress–mediated cytotoxic mechanisms, although clinical evidence remains mixed and context-dependent [246]. In all cases, the risk of interaction is amplified in polypharmacy settings, particularly in elderly populations with multimorbidity.

### 8.2. Toxicity Risks

While most naturally derived compounds exhibit favorable safety profiles at nutritional doses, toxicity risks emerge at pharmacological or supra-nutritional intake levels, particularly when concentrated extracts or high-dose supplementation is used.

Red yeast rice, for example, contains monacolin K, which is structurally identical to lovastatin and may produce statin-like adverse effects, including hepatotoxicity, myopathy, and in rare cases rhabdomyolysis, especially when combined with other lipid-lowering agents [247]. Similarly, excessive intake of green tea extracts (EGCG) has been associated with hepatotoxicity in susceptible individuals [248].

High-dose supplementation of fat-soluble vitamins and antioxidant compounds may also produce pro-oxidant effects under certain conditions, potentially disrupting endogenous redox balance [249]. Alkaloids such as berberine, while generally safe at controlled doses, may cause gastrointestinal side effects, hypotension, or hypoglycemia when used in combination with conventional therapies [245].

Even omega-3 fatty acids, widely regarded as safe, may increase bleeding risk at very high doses and can cause gastrointestinal discomfort or lipid peroxidation-related effects in susceptible individuals [250]. Importantly, toxicity risk is often dose-dependent and influenced by formulation purity, with poorly standardized products posing greater risk due to contaminant variability [251,252].

### 8.3. Overuse and Misuse of Supplements

A significant public health concern is the increasing overuse and inappropriate self-administration of dietary supplements without clinical indication or professional supervision. This trend is driven in part by the perception of natural products as inherently safe and beneficial, despite limited evidence for efficacy in many contexts [253].

Overuse may lead to unnecessary financial burden, delayed access to evidence-based medical treatments, and potential masking of underlying disease progression. In cardiovascular and metabolic conditions, reliance on nutraceuticals in place of pharmacological therapies such as statins, antihypertensives, metformin, GLP-1 receptor agonists, or SGLT2 inhibitors may result in suboptimal disease control and increased long-term risk [231].

Misuse is also evident in polypharmacy involving multiple overlapping supplements, leading to unregulated cumulative dosing and increased risk of adverse interactions. This is particularly relevant in polyphenol-rich supplements, antioxidant combinations, and multi-ingredient “cardiometabolic health” formulations, where synergistic safety profiles are poorly characterized [254].

In neurodegenerative and oncological contexts, supplementation is sometimes pursued as an alternative rather than adjunctive strategy, despite the lack of evidence for disease-modifying effects, potentially delaying clinically effective interventions [246].

### 8.4. Regulatory and Quality Control Issues

One of the most critical limitations affecting safety is the lack of uniform regulatory oversight and quality control across nutraceutical and herbal product markets. Unlike pharmaceutical agents, many natural products are classified as dietary supplements in several jurisdictions, allowing them to bypass rigorous pre-market efficacy and safety evaluation. This regulatory gap leads to substantial variability in product composition, purity, labeling accuracy, and batch-to-batch consistency. Studies have documented discrepancies between labeled and actual concentrations of active compounds, as well as contamination with heavy metals, pesticides, microbial contaminants, or undeclared pharmacologically active substances [251,252,255]. Red yeast rice products, for instance, may contain variable levels of monacolin K and, in some cases, nephrotoxic contaminants such as citrinin [256]. Similarly, herbal preparations may differ significantly depending on geographic origin, extraction method, and manufacturing standards.

The lack of harmonized global standards for probiotics further complicates safety evaluation, as strain identification, viability, and CFU counts are not consistently verified across commercial formulations. This variability undermines both efficacy and safety assessments, making risk–benefit evaluation difficult in clinical practice. Furthermore, insufficient post-marketing surveillance systems for supplements limit detection of rare or long-term adverse effects, creating gaps in pharmacovigilance compared to conventional pharmaceuticals.

### 8.5. Overall Safety Perspective

In summary, while natural products and microbiome-based interventions are generally associated with favorable safety profiles at dietary levels, their clinical use is constrained by a combination of herb–drug interactions, dose-dependent toxicity risks, widespread misuse, and inconsistent regulatory oversight. These factors highlight the necessity of integrating evidence-based guidance, standardized product regulation, and clinical supervision into the use of nutraceuticals, particularly in populations with chronic disease or concurrent pharmacotherapy. A balanced safety framework is therefore essential to ensure that the potential benefits of these interventions are realized without compromising patient safety or therapeutic efficacy.

## 9. Regulatory and Standardization Perspectives

**Figure 6:**
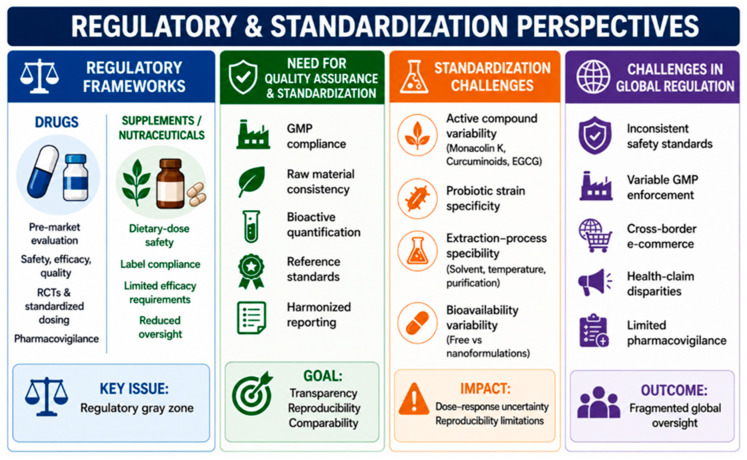
Key regulatory and standardization frameworks relevant to natural products.

Natural products have shown considerable therapeutic potential, but their clinical translation remains limited by regulatory and standardization challenges. Unlike pharmaceuticals, nutraceuticals, botanical extracts, and probiotics are often regulated under less stringent frameworks, resulting in variability in product quality, composition, efficacy claims, and safety monitoring. In addition, differences in raw materials, manufacturing processes, formulation, and bioavailability can significantly affect clinical outcomes. Therefore, improved regulatory harmonization, quality assurance, and product standardization are essential for enhancing the reliability, reproducibility, and clinical integration of natural products. The most important regulatory and standardization perspectives of natural products are depicted in Figure 6.

### 9.1. Current Regulatory Frameworks (Supplements vs. Drugs)

The regulatory classification of natural products varies significantly across jurisdictions and represents a fundamental determinant of their development, marketing, and clinical integration. In most regions, including the United States and the European Union, a clear distinction exists between pharmaceutical drugs, which require rigorous pre-market evaluation of safety, efficacy, and manufacturing consistency, and dietary supplements or nutraceuticals, which are regulated under less stringent frameworks.

Under supplement-based regulatory systems, products such as polyphenol extracts, omega-3 formulations, probiotics, herbal preparations (e.g., curcumin, berberine, garlic extracts), and fiber-based supplements are generally permitted for market release without requiring demonstration of clinical efficacy for disease treatment or prevention. Instead, regulatory oversight is typically limited to safety at recommended dietary doses and compliance with labeling standards. This contrasts sharply with drug approval pathways, which require large-scale randomized controlled trials, standardized dosing, and post-marketing surveillance.

As a result, many interventions discussed in this review—including red yeast rice, plant sterols, probiotics, and botanical extracts—exist in a regulatory “gray zone,” where products may exert pharmacologically active effects but are not held to the same evidentiary standards as conventional therapeutics. This regulatory asymmetry contributes directly to variability in clinical outcomes and complicates integration into evidence-based medical guidelines.

### 9.2. Need for Quality Assurance and Standardization

A central challenge in the clinical application of natural products is the lack of robust quality assurance and standardization mechanisms across manufacturing, formulation, and reporting practices. Unlike synthetic pharmaceuticals, which are chemically defined and manufactured under strict Good Manufacturing Practice (GMP) conditions, many natural products are derived from complex biological sources with inherent variability in composition.

### 9.3. Standardization Issues

Standardization issues in nutraceuticals and natural products arise at multiple levels, reflecting the complexity of their composition and production. A primary concern is the variability in active compound concentrations, as the levels of key bioactive constituents—such as monacolin K in red yeast rice, allicin in garlic, curcuminoids in turmeric, and EGCG in green tea extracts—can differ markedly across commercial preparations. These differences are often driven by variations in raw materials, agricultural conditions, and manufacturing practices, resulting in inconsistent biological activity and therapeutic potential [228].

In addition, probiotic products present unique challenges related to strain specificity. The clinical effects of probiotics are highly dependent on the precise microbial strain used, yet many products do not provide adequate strain-level identification or ensure viability through to the point of consumption. This lack of detailed labeling and quality assurance complicates the interpretation of clinical outcomes and limits reproducibility across studies and products [257].

Extraction and processing methods further contribute to variability. Factors such as the choice of solvent, temperature conditions, and purification techniques can significantly influence the phytochemical profile, stability, and potency of the final product. As a result, even products derived from the same natural source may exhibit substantial differences in composition and efficacy [258].

Finally, inconsistencies in bioavailability represent another critical dimension of standardization. Variations in formulation—such as the use of free compounds compared with advanced delivery systems like phospholipid complexes, emulsions, or nanoformulations—can markedly affect pharmacokinetics and systemic exposure. These differences often translate into heterogeneous clinical effects, even when nominally equivalent doses of the same compound are administered [259]. Together, these factors underscore the need for improved standardization, quality control, and transparency in the development and regulation of nutraceutical products. These inconsistencies undermine dose–response relationships, limit reproducibility, and reduce comparability across clinical trials. Consequently, there is a growing need for standardized reference preparations, validated bioactive quantification methods, and harmonized reporting guidelines to improve transparency and reproducibility in nutraceutical research.

### 9.4. Challenges in Global Regulation

Global regulation of natural products is further complicated by substantial heterogeneity across international regulatory frameworks, leading to fragmented oversight and inconsistent product quality standards. While some countries apply relatively stringent requirements for health claims and manufacturing standards, others operate under more permissive frameworks that prioritize market access over clinical validation [260,261]. This lack of harmonization results in several key challenges:

- Inconsistent safety and efficacy standards: A product approved as a dietary supplement in one jurisdiction may be classified differently—or require prescription-level regulation—in another. This creates disparities in consumer access, clinical guidance, and risk management.
- Variable enforcement of manufacturing standards: Even where Good Manufacturing Practice (GMP) guidelines exist, enforcement intensity and inspection frequency vary widely, leading to uneven product quality across global markets.
- Cross-border trade and online distribution: The expansion of e-commerce has enabled widespread international distribution of nutraceuticals, often bypassing national regulatory controls and increasing exposure to substandard or adulterated products.
- Health claims regulation disparities: The permissibility of disease-related claims differs significantly, with some regions allowing structure-function claims (e.g., “supports cardiovascular health”) while prohibiting explicit therapeutic claims, further complicating consumer interpretation and clinical integration.

Limited global pharmacovigilance integration: Adverse event reporting systems for supplements are less developed and poorly coordinated internationally, limiting the detection of rare or long-term safety signals.

### 9.5. Overall Regulatory Perspective

Overall, the regulatory landscape for natural products remains fragmented, heterogeneous, and insufficiently aligned with their pharmacological potential. The current separation between “food supplements” and “drugs” does not adequately reflect the increasing clinical relevance of bioactive compounds such as omega-3 fatty acids, berberine, plant sterols, and probiotic strains, which often exert drug-like biological effects.

To address these limitations, future progress will require greater regulatory convergence, enhanced quality control systems, mandatory standardization of active constituents, and improved global harmonization of safety and efficacy requirements. Such reforms are essential to bridge the gap between mechanistic promise and clinically reliable application and to ensure that natural product–based interventions can be integrated safely and effectively into evidence-based healthcare systems.

## 10. Future Directions and Research Priorities and Translation Perspectives of Natural Products

**Figure 7:**
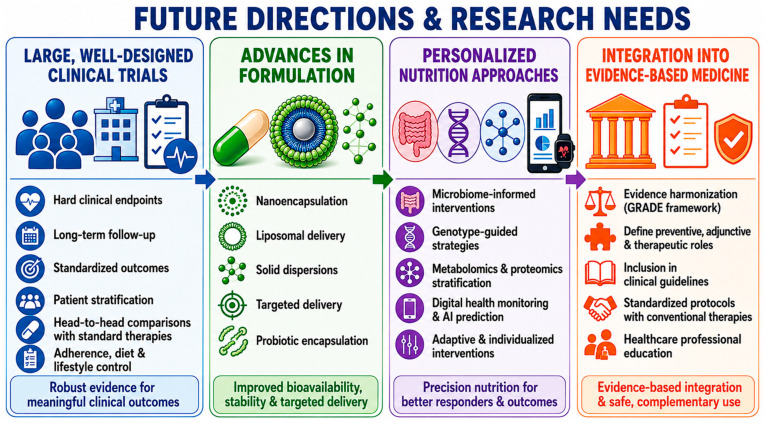
Future directions, research priorities, and translational perspectives of natural products.

Despite substantial progress in elucidating the mechanistic and clinical effects of natural products and microbiome-targeted interventions across cardiovascular, metabolic, neurodegenerative, oncological, gastrointestinal, and infectious disease domains, the field remains constrained by significant translational gaps. Addressing these limitations will require coordinated advances in clinical trial design, formulation science, precision nutrition, and integration into mainstream evidence-based medical frameworks. Future directions, research priorities, and translational perspectives of natural products are summarized in Figure 7.

### 10.1. Need for Large, Well-Designed Clinical Trials

A primary research priority is the development of large-scale, multicenter, RCTs with sufficient statistical power to evaluate hard clinical endpoints such as cardiovascular events, cancer progression, neurodegeneration, and infection-related morbidity and mortality. Current evidence is heavily dominated by small to moderate-sized trials focused on surrogate biomarkers (e.g., LDL-C, HbA1c, CRP, endothelial function, or body weight), which, while mechanistically informative, do not consistently translate into clinically meaningful outcomes. Future trials should prioritize:

- Long-term follow-up durations to assess durability of effects and disease-modifying potential;
- Standardized outcome measures across studies to improve comparability and meta-analytic synthesis;
- Stratification by baseline metabolic, inflammatory, and microbiome profiles to reduce heterogeneity;
- Head-to-head comparisons with conventional pharmacological therapies, particularly in cardiometabolic disease;
- Rigorous assessment of adherence, dietary background, and lifestyle confounders.

Such designs are particularly critical in neurodegenerative diseases and oncology, where current evidence is weakest and translational failure is most pronounced despite strong preclinical rationale.

### 10.2. Advances in Formulation

A key translational barrier across most natural products is poor bioavailability and pharmacokinetic instability, particularly for polyphenols, flavonoids, and certain alkaloids. Future progress will depend heavily on advances in formulation science and drug delivery technologies designed to enhance systemic exposure, target specificity, and metabolic stability. Promising strategies include:

- Nanoencapsulation systems (lipid nanoparticles, polymeric nanocarriers);
- Liposomal and phospholipid-based delivery systems to improve intestinal absorption;
- Solid dispersion technologies to enhance solubility of poorly water-soluble compounds (e.g., curcumin, resveratrol);
- Targeted delivery platforms designed to enhance tissue-specific accumulation, including vascular or tumor microenvironment targeting;
- Probiotic encapsulation technologies to improve strain viability and gut colonization efficiency.

These approaches may help bridge the gap between strong in vitro bioactivity and limited in vivo efficacy, particularly in systemic diseases such as cancer and neurodegeneration, where tissue accessibility remains a major limiting factor.

### 10.3. Personalized Nutrition Approaches

Emerging evidence strongly supports the concept that responses to dietary bioactive compounds are highly individualized, driven by variability in gut microbiome composition, genetic background, metabolic phenotype, immune status, and disease stage. This inter-individual heterogeneity is a major contributor to inconsistent clinical trial outcomes and represents a key opportunity for precision medicine approaches. Future research should focus on:

- Microbiome-informed dietary and nutraceutical interventions (e.g., SCFA-producing capacity, bile acid metabolism profiles);
- Genotype-guided supplementation strategies targeting lipid metabolism, inflammation, and oxidative stress pathways;
- Metabolomics- and proteomics-based stratification to identify responders and non-responders;
- Integration of digital health tools (wearables, dietary tracking, AI-based prediction models) to monitor real-time physiological responses;
- Adaptive intervention models that dynamically adjust dose, formulation, or combination therapy based on individual response profiles.

Such personalized strategies may be particularly impactful in metabolic disorders, gastrointestinal diseases, and cardiovascular risk management, where variability in response to fiber, probiotics, omega-3 fatty acids, and polyphenols is especially pronounced.

### 10.4. Integration into Evidence-Based Medicine

A central long-term objective is the systematic integration of validated natural product interventions into evidence-based clinical practice. At present, most nutraceuticals occupy an intermediate space between nutrition and pharmacology, limiting their incorporation into formal treatment guidelines despite growing evidence for selected interventions. Future integration will require:

- Harmonization of clinical trial evidence with guideline development frameworks (e.g., GRADE methodology);
- Clear differentiation between adjunctive, preventive, and therapeutic roles of natural products;
- Inclusion of high-quality nutraceutical evidence in cardiometabolic, gastrointestinal, and preventive medicine guidelines;
- Development of standardized clinical protocols for combined use with conventional pharmacotherapies;
- Education of healthcare professionals regarding evidence strength, safety profiles, and interaction risks.

Importantly, integration should not imply substitution of established therapies but rather evidence-based complementarity, particularly in cardiovascular disease prevention, metabolic risk reduction, and gastrointestinal symptom management.

### 10.5. Overall Outlook

Collectively, the future of natural products lies in transitioning from a heterogeneous and largely biomarker-driven field toward a standardized, mechanistically informed, and clinically validated discipline. Achieving this will require convergence of large-scale clinical evidence generation, advanced delivery technologies, precision nutrition frameworks, and regulatory alignment. Together, these advances will determine whether current mechanistic promise can be translated into robust, reproducible, and clinically meaningful therapeutic strategies across disease domains.

## 11. Conclusions

This review highlights that natural products and microbiome-targeted interventions exert multi-target biological effects across cardiovascular, metabolic, neurodegenerative, oncological, gastrointestinal, and infectious disease domains, primarily through modulation of lipid metabolism, inflammation, oxidative stress, endothelial function, immune signaling, and host–microbiome interactions. Across all disease categories, a consistent pattern emerges: however, strong mechanistic plausibility and favorable effects on intermediate biomarkers do not uniformly translate into robust, consistent improvements in hard clinical outcomes. Moreover, a central theme emerging from the present review is that many natural products exert pleiotropic effects on biological pathways that are common to multiple disease states, thereby supporting a mechanism-oriented rather than disease-oriented perspective on their therapeutic potential.

The most compelling clinical evidence is observed in cardiovascular disease prevention, particularly for Mediterranean dietary patterns, high-dose EPA-based omega-3 interventions, plant sterols, and dietary fiber, where improvements in lipid profiles, vascular function, and, in select cases, cardiovascular events have been demonstrated. In metabolic disorders and gastrointestinal diseases, interventions such as fiber, probiotics, polyphenols, and berberine show modest but clinically relevant improvements in glycemic control, body composition, and gastrointestinal symptoms, although effects are highly variable and context-dependent.

In contrast, neurodegenerative diseases, cancer, and infectious diseases exhibit a pronounced translational gap, where strong preclinical efficacy and mechanistic rationale are not consistently reflected in clinical outcomes. This discrepancy is largely attributable to pharmacokinetic limitations, late-stage intervention timing, disease complexity, and heterogeneity in patient populations.

A recurring theme across disease categories is the discrepancy between promising mechanistic and preclinical findings and the more modest or inconsistent effects observed in human studies. Hence, future research should prioritize adequately powered, well-standardized clinical trials designed to improve reproducibility, characterize responder subgroups, and determine the clinical significance of observed effects.

Across all domains, key limiting factors—including poor bioavailability, variability in natural product composition, lack of standardization, limited large-scale trials, and reproducibility challenges—consistently constrain the strength and reliability of clinical conclusions. These issues are further compounded by regulatory fragmentation and inconsistent quality control standards, which hinder reproducibility and clinical translation.

Overall, the clinical relevance of natural products lies in their ability to act as multi-target modulators of complex biological systems, particularly the diet–microbiome–host axis. However, their effective integration into clinical practice requires rigorous standardization, improved bioavailability strategies, and high-quality clinical evidence to ensure consistent, reproducible, and clinically meaningful outcomes.

## Figures and Tables

**Table 1:** Classification of natural products according to their chemical class and subclass, representative bioactive compounds, and principal dietary sources.

Class/Subclass	Chemical Category	Representative Compounds	Dietary Sources
**Phenolic acids**	Hydroxybenzoic acids	Gallic acid, protocatechuic acid, vanillic acid, syringic acid, salicylic acid, gentisic acid, ellagic acid	Berries (strawberry, blueberry), pomegranate, tea, red wine, walnuts
	Hydroxycinnamic acids	Caffeic acid, ferulic acid, p-coumaric acid, sinapic acid, chlorogenic acid, rosmarinic acid	Coffee, whole grains, sunflower seeds, herbs (rosemary, oregano), artichokes
**Flavonoids**	Flavonols	Quercetin, kaempferol, myricetin, isorhamnetin, rutin, fisetin, morin	Onions, apples, kale, broccoli, capers, berries
	Flavones	Luteolin, apigenin, chrysin, baicalein, wogonin	Parsley, celery, chamomile, thyme, oregano
	Flavan-3-ols	Catechin, epicatechin, EGCG, gallocatechin	Green tea, black tea, cocoa, dark chocolate, grapes
	Flavanones	Naringenin, hesperetin, eriodictyol, hesperidin	Oranges, lemons, grapefruits, mandarins
	Anthocyanins	Cyanidin, delphinidin, malvidin, pelargonidin	Blueberries, blackberries, cherries, red cabbage, purple sweet potato
	Chalcones	Phloretin, phloridzin, xanthohumol	Apples, hops, beer
	Isoflavones	Genistein, daidzein, glycitein	Soybeans, tofu, tempeh, soy milk
**Stilbenes**	Stilbene derivatives	Resveratrol, pterostilbene, piceatannol	Grapes, red wine, peanuts, blueberries
**Lignans**	Plant lignans and enterolignans	Secoisolariciresinol, matairesinol, pinoresinol	Flaxseed, sesame seeds, whole grains, berries
**Tannins**	Hydrolyzable tannins	Ellagitannins, gallotannins	Pomegranate, walnuts, raspberries
	Condensed tannins	Procyanidins	Cocoa, grapes, apples, cranberries
**Alkaloids**	Purine alkaloids	Caffeine, theobromine, theophylline	Coffee, tea, cocoa, energy drinks
	Isoquinoline alkaloids	Berberine	Barberry, goldenseal
	Indole alkaloids	Tryptamine derivatives	Fermented foods, medicinal plants
	Capsaicinoids	Capsaicin	Chili peppers, paprika
**Omega fatty acids**	Omega-3 PUFA	α-Linolenic acid, EPA, DHA	Fatty fish (salmon, mackerel, sardines), flaxseed, chia seeds, walnuts, algae oils
	Omega-6 PUFA	Linoleic acid, arachidonic acid	Corn oil, soybean oil, sunflower oil, nuts, seeds
**Carotenoids**	Carotenes	β-carotene, lycopene	Carrots, tomatoes, pumpkin, sweet potatoes
	Xanthophylls	Lutein, zeaxanthin, astaxanthin	Spinach, kale, egg yolk, corn, microalgae
**Terpenoids**	Monoterpenes	Limonene, menthol	Citrus fruits, mint, herbs
	Sesquiterpenes	Artemisinin	Herbs, spices
	Diterpenes	Carnosic acid, cafestol	Rosemary, coffee
	Triterpenes	Ursolic acid, oleanolic acid	Apples, olives, basil
**Organosulfur compounds**	Thiosulfinates	Allicin	Garlic, onions, leeks
	Isothiocyanates	Sulforaphane	Broccoli, Brussels sprouts, kale
	Glucosinolates	Glucoraphanin	Cruciferous vegetables
**Phytosterols**	Sterols	β-sitosterol, campesterol	Vegetable oils, nuts, seeds, avocados
	Stanols	Sitostanol	Fortified spreads, functional foods
**Saponins**	Triterpenoid saponins	Ginsenosides	Ginseng, legumes
	Steroidal saponins	Diosgenin	Yams, chickpeas
**Polysaccharides**	Soluble fibers	β-glucans, pectins	Oats, barley, apples, citrus fruits
	Prebiotics	Inulin, FOS	Chicory root, onions, garlic, asparagus, bananas
**Peptides and proteins**	Bioactive peptides	IPP, VPP, lunasin	Fermented dairy (yogurt, cheese), soy products
**Organic acids**	Metabolic acids	Citric acid, malic acid	Citrus fruits, apples, fermented foods
**Vitamins**	Fat-soluble	Vitamins A, D, E, K	Liver, fish oils, dairy, leafy greens
	Water-soluble	Vitamins C, B-complex	Fruits, vegetables, whole grains, meat
**Coenzymes**	Quinones and cofactors	CoQ10, lipoic acid	Organ meats, fish, whole grains
**Endogenous antioxidants**	Redox regulators	Glutathione	Avocado, spinach, asparagus
**Advanced lipid mediators**	SPMs	Resolvins, protectins	Derived from omega-3 (fish oils)
**Eicosanoids**	Lipid mediators	Prostaglandins	Endogenously formed from dietary PUFA
**Phytoestrogens**	Non-isoflavone	Coumestrol	Alfalfa sprouts, legumes
**Indoles**	Indole derivatives	Indole-3-carbinol	Broccoli, cabbage, cauliflower
**Nucleotides**	Purine derivatives	Adenosine	Meat, fish, yeast extracts
**Postbiotics**	SCFAs	Butyrate	Produced from fiber (whole grains, legumes)
	Indole metabolites	Indolepropionic acid	Gut microbiota (fiber-rich diets)
	Secondary bile acids	Deoxycholic acid	Gut metabolism

EGCG: Epigallocatechin Gallate; PUFAs: Poly-Unsaturated Fatty Acids; EPA: Eicosapentaenoic acid; DHA: Docosahexaenoic acid; FOS: Fructooligosaccharides; IPP: Isoleucine-Proline-Proline (Lactotripeptide); VPP: Valine-Proline-Proline (Lactotripeptide); CoQ10: Coenzyme Q10; SPMs: Specialized Pro-resolving Mediators; SCFAs: Short-Chain Fatty Acids.

**Table 2:** Preclinical evidence of natural compounds, their key molecular mechanisms, and biological outcomes in experimental models (↓: decrease, ↑: increase).

Disease Category	Compound/Class	Model Type	Key Mechanisms	Preclinical Outcome
Cardiovascular Diseases	Red yeast rice (monacolin K)	Animal	HMG-CoA reductase inhibition	↓ Total cholesterol, ↓ LDL-C, ↓ VLDL; improved hepatic lipid clearance; reduced aortic lipid deposition and early atherosclerotic lesion size in high-fat diet models
	Berberine Berberis (barberry plant)	In vitro + Animal	↑ LDL receptor (ERK/JNK), AMPK activation	↓ LDL-C and triglycerides; ↑ hepatic LDL uptake; improved insulin sensitivity; reduced hepatic steatosis in metabolic syndrome models
	Plant sterols/fibers	Animal	↓ cholesterol absorption, ↑ bile acid excretion	↓ intestinal cholesterol uptake; ↑ fecal sterol loss; improved plasma lipid ratios (↓ LDL/HDL); attenuation of diet-induced hypercholesterolemia
	Curcumin Turmeric, Resveratrol Grapes, EGCG Green tea	In vitro + Animal	NF-κB inhibition, NLRP3 suppression	↓ Pro-inflammatory cytokines (TNF-α, IL-1β, IL-6); reduced macrophage infiltration; decreased foam cell formation; improved fibrous cap stability in ApoE−/− mice
	Sulforaphane Broccoli sprouts, CoQ10, Cocoa flavanols	In vitro + Animal	Nrf2 activation	↑ SOD, HO-1, GPx; ↓ ROS and lipid peroxidation (MDA); reduced oxidized LDL accumulation; improved endothelial resistance to oxidative injury
	Beetroot nitrates Beetroot	Animal	NO/eNOS activation	↑ Nitric oxide bioavailability; improved vasodilation; ↓ vascular resistance; enhanced endothelial-dependent relaxation in arterial ring assays
	Garlic, omega-3 Fish oil	Animal	COX-1 inhibition	↓ Platelet aggregation; reduced thromboxane A2 synthesis; mild anti-thrombotic effect with limited clot formation delay in ex vivo assays
	Polyphenols, fiber	Animal	Gut microbiota modulation (↓ TMAO, ↑ SCFAs)	↑ SCFA production (butyrate, propionate); ↓ circulating TMAO; improved bile acid metabolism; reduced atherosclerotic plaque burden in microbiome-modulated models
Diabetes Type 2	Curcumin Turmeric, Resveratrol Grapes, Quercetin	Animal	↓ PI3K/Akt, AMPK activation, NF-κB inhibition, ↑ Nrf2	↓ Fasting glucose; ↓ HbA1c (model-dependent); ↑ GLUT4 translocation; improved insulin sensitivity index; protection of pancreatic β-cell mass
	Polyphenols	Animal	↓ gluconeogenesis, ↑ glucose uptake	↓ Hepatic glucose output; ↑ skeletal muscle glucose uptake; improved glucose tolerance curves (OGTT normalization in diabetic rodents)
	Probiotics	Animal	Gut microbiota modulation	Increased SCFA production; improved insulin signaling; highly variable effects on glucose control depending on strain and host microbiota baseline
Obesity	Polyphenols, fiber metabolites	Animal	AMPK activation, ↓ adipogenesis, ↑ thermogenesis	↓ Body weight gain; ↓ visceral fat mass; suppression of adipocyte differentiation (↓ PPARγ); increased UCP1-mediated thermogenesis in brown adipose tissue
	SCFAs	Animal	GPCR signaling, GLP-1/PYY release	↑ Satiety signaling; ↓ caloric intake; improved energy harvest efficiency; reduced adiposity accumulation over long-term feeding studies
	Omega-3 fish oil	Animal	Anti-inflammatory lipid mediators	↓ Adipose tissue inflammation (↓ macrophage infiltration); improved insulin sensitivity; modest or inconsistent reduction in total body weight
Alzheimer’s Disease	Omega-3 Fish oil	Animal	↓ Aβ production, mitochondrial support	↓ Amyloid plaque burden; improved synaptic density; enhanced spatial memory performance (Morris water maze improvement)
	Curcumin Turmeric, EGCG Green tea	Animal	NF-κB inhibition, Nrf2 activation, ↓ tau phosphorylation	↓ Amyloid aggregation; reduced tau hyperphosphorylation; improved neuronal survival in hippocampal regions
	Flavonoids	Animal	↑ BDNF, ↑ LTP	↑ Synaptic plasticity; enhanced long-term potentiation; improved learning and memory performance in behavioral assays
	SCFAs	Animal	HDAC inhibition	Epigenetic regulation of neuroinflammation; improved microglial homeostasis; neuroprotection against cognitive decline
Parkinson’s Disease	Polyphenols, omega-3, SCFAs	Animal	Nrf2 activation, mitochondrial protection	↑ Dopaminergic neuron survival in substantia nigra; ↓ motor deficits; improved locomotor activity and balance tests
Cancer	Curcumin Turmeric, EGCG Green tea	In vitro + Animal	NF-κB, STAT3, PI3K/Akt inhibition	↑ Apoptosis (caspase activation); ↓ tumor volume; ↓ angiogenesis (VEGF suppression); reduced metastasis markers
	Sulforaphane Broccoli sprouts	In vitro + Animal	Nrf2 activation	Detoxification enzyme induction; reduced carcinogen-induced DNA damage; suppression of tumor initiation and progression
	Berberine Berberis (barberry plant)	In vitro + Animal	AMPK activation	Induction of metabolic stress in tumor cells; inhibition of proliferation; cell cycle arrest (G1/S checkpoint)
	Omega-3 Fish oil	Animal	Lipid mediator shift	↓ Tumor-associated inflammation; altered tumor microenvironment favoring immune infiltration
	SCFAs (butyrate)	In vitro + Animal	HDAC inhibition	Selective apoptosis in cancer cells; epigenetic reprogramming; reduced tumor growth in colon cancer models
	Microbial metabolites	Animal	AhR signaling, immune modulation	Modulation of tumor immune microenvironment; improved anti-tumor immune surveillance
Gastrointestinal Disorders	Probiotics	In vitro + Animal	Gut barrier strengthening, immune modulation	↓ Mucosal inflammation; ↑ tight junction protein expression; reduced intestinal permeability (“leaky gut”); improved epithelial repair rate
	Prebiotic fibers	Animal	SCFA production, microbiota shifts	↑ Butyrate production; improved colonocyte energy supply; reduced DSS-induced colitis severity; restoration of microbiota diversity
	Curcumin Turmeric	Animal	NF-κB inhibition	↓ Colonic inflammation; reduced ulceration; improved histological scores of mucosal damage
	EGCG Green tea	Animal	Antioxidant, anti-inflammatory	↓ Oxidative mucosal injury; improved epithelial regeneration; reduced neutrophil infiltration
	SCFAs	Animal	GPCR signaling, epithelial metabolism	Improved epithelial barrier integrity; ↑ mucin production; reduced inflammatory cytokine expression in colon tissue
Infectious Diseases	Curcumin Turmeric	In vitro + Animal	NF-κB inhibition, antimicrobial effects	↓ Bacterial proliferation; reduced viral replication; decreased pathogen-induced inflammatory response
	EGCG Green tea	In vitro + Animal	Viral entry inhibition	↓ Viral infectivity; inhibition of viral attachment and replication; antioxidant-mediated immune support
	Allicin (garlic), Garlic	In vitro + Animal	Membrane disruption	Broad-spectrum antimicrobial activity; bacterial growth inhibition; disruption of microbial enzymes
	Probiotics	Animal	Competitive exclusion, immune modulation	↓ Pathogen colonization; enhanced mucosal immunity; increased antimicrobial peptide expression
	Polyphenols	In vitro + Animal	Protein binding, enzyme inhibition	Reduced microbial growth; inhibition of bacterial quorum sensing; antiviral binding interference
	SCFAs	Animal	Immune regulation, barrier enhancement	↓ Infection susceptibility; improved epithelial barrier defense; modulation of innate immune responses

**Table 3:** Summary of human clinical evidence for natural products across major disease categories (↓: decrease).

Disease Area	Intervention (s)	Main Clinical Outcome	Key Limitations	Representative References
**Cardiovascular Disease**	Omega-3 fatty acids (EPA/DHA)	↓ Triglycerides; modest reduction in MACE, particularly with purified EPA formulations	Mixed EPA/DHA preparations yield heterogeneous results	[194]
	Red yeast rice	↓ LDL-C comparable to low-intensity statins; improved lipid profile	Variability in monacolin K content, product quality, and regulatory standardization	[73,195]
	Plant sterols, β-glucans, psyllium	Dose-dependent ↓ LDL-C through reduced cholesterol absorption and increased bile acid excretion	Effects mainly limited to lipid endpoints	[67,196]
	Cocoa flavanols	Improved endothelial function and vascular reactivity	Variable formulation and dosage	[67,196]
	Dietary nitrates (beetroot, leafy vegetables)	↓ Blood pressure via nitric oxide-mediated mechanisms	Long-term outcome data limited	[67,196]
	Garlic, CoQ10, polyphenols	Modest improvements in cardiometabolic risk factors	Effects generally smaller and less consistent than pharmacotherapy	[197,198,199]
	Mediterranean diet	Reduced cardiovascular events and cardiovascular mortality	Adherence-dependent effects	[200]
**Metabolic Disorders (T2DM, Obesity)**	Berberine	↓ Fasting glucose, HbA1c, LDL-C, triglycerides, insulin resistance	High heterogeneity in dosage, formulation, and study design	[201]
	Dietary fiber	Improved satiety, postprandial glycemia, microbiota composition	Benefits often require sustained intake	[202,203]
	Curcumin and polyphenols	Improved glycemic control, insulin sensitivity, lipid metabolism	Variable bioavailability and clinical response	[201,202,203]
	Fiber- and polyphenol-rich interventions	Small reductions in body weight, BMI, body fat, and waist circumference	Benefits often diminish after intervention cessation	[202,203]
	Probiotics	Variable effects on glycemic control and obesity outcomes	Strain-specific effects and microbiome heterogeneity	[204,205]
**Neurodegenerative Diseases**	Omega-3 fatty acids	Improved inflammatory and oxidative stress biomarkers	No consistent effect on disease progression or cognition	[194,206]
	Flavonoids, curcumin, resveratrol, polyphenols	Improvements in neurotrophic, inflammatory, and oxidative biomarkers; occasional cognitive benefits	Limited BBB penetration, poor bioavailability, late intervention	[199,206,207]
**Cancer**	Omega-3 fatty acids	Improved cachexia, inflammation, metabolic status, treatment tolerance	No reproducible survival benefit	[208,209,210,211]
	Polyphenols, dietary fiber, probiotics, SCFAs	Improved quality of life, gastrointestinal symptoms, inflammatory status	Lack of tumor regression or survival benefit as monotherapy	[208,209,210,211]
**Gastrointestinal Disorders**	Probiotics	Improved IBS symptoms, barrier integrity, remission maintenance in IBD	Highly strain-specific responses	[212,213,214,215,216]
	Dietary fiber	Improved bowel function, symptom severity, microbiota composition	Variable responses across disease phenotypes	[212,213,214,215,216]
	Curcumin and selected polyphenols	Reduced inflammatory biomarkers and improved disease activity in IBD	Formulation-dependent efficacy	[212,213,214,215,216]
**Infectious Diseases**	Probiotics and synbiotics	Prevention of antibiotic-associated diarrhea, recurrent Clostridioides difficile infection, and gastrointestinal infections	Modest effects and strain specificity	[217,218,219,220]
	Selected probiotics	Reduced incidence/duration of respiratory tract infections	Variable across populations and settings	[221,222]
	Polyphenols and flavonoids	Antimicrobial, anti-virulence, and immunomodulatory effects	Poor bioavailability and pharmacokinetic limitations	[223,224]
	Berberine	Antimicrobial, anti-inflammatory, microbiome-modulating effects	Formulation challenges, safety concerns, limited high-quality trials	[225,226]
	Omega-3 fatty acids and SPMs	Enhanced inflammation resolution and tissue recovery	Primarily host-directed rather than antimicrobial effects	[53,227]

**Abbreviations:** MACE, major adverse cardiovascular events; LDL-C, low-density lipoprotein cholesterol; HbA1c, glycated hemoglobin; T2DM, type 2 diabetes mellitus; IBS, irritable bowel syndrome; IBD, inflammatory bowel disease; SCFAs, short-chain fatty acids; SPMs, specialized pro-resolving mediators; BBB, blood–brain barrier.

## Data Availability

No new data were created or analyzed in this study. Data sharing is not applicable to this article.

## References

[1] Atanasov A.G., Zotchev S.B., Dirsch V.M., Supuran C.T. (2021). Natural Products in Drug Discovery: Advances and Opportunities. Nat. Rev. Drug Discov..

[2] Pan S.Y., Litscher G., Gao S.H., Zhou S.F., Yu Z.L., Chen H.Q., Zhang S.F., Tang M.K., Sun J.N., Ko K.M. (2014). Historical Perspective of Traditional Indigenous Medical Practices: The Current Renaissance and Conservation of Herbal Resources. Evid.-Based Complement. Altern. Med..

[3] Newman D.J., Cragg G.M. (2020). Natural Products as Sources of New Drugs over the Nearly Four Decades from 01/1981 to 09/2019. J. Nat. Prod..

[4] Cory H., Passarelli S., Szeto J., Tamez M., Mattei J. (2018). The Role of Polyphenols in Human Health and Food Systems: A Mini-Review. Front. Nutr..

[5] Adal E., Aktar T. (2024). The Impact of Polyphenols on Nutrition and Health. Turk. J. Agric. Food Sci. Technol..

[6] Li W., Chen H., Xu B., Wang Y., Zhang C., Cao Y., Xing X. (2023). Research Progress on Classification, Sources and Functions of Dietary Polyphenols for Prevention and Treatment of Chronic Diseases. J. Future Foods.

[7] Rudrapal M., Rakshit G., Singh R.P., Garse S., Khan J., Chakraborty S. (2024). Dietary Polyphenols: Review on Chemistry/Sources, Bioavailability/Metabolism, Antioxidant Effects, and Their Role in Disease Management. Antioxidants.

[8] Saad A.M., Mohammed D.M., Alkafaas S.S., Ghosh S., Negm S.H., Salem H.M., Fahmy M.A., Semary H.E., Ibrahim E.H., AbuQamar S.F. (2025). Dietary Polyphenols and Human Health: Sources, Biological Activities, Nutritional and Immunological Aspects, and Bioavailability—A Comprehensive Review. Front. Immunol..

[9] Quesada-Vázquez S., Eseberri I., Les F., Pérez-Matute P., Herranz-López M., Atgié C., Lopez-Yus M., Aranaz P., Oteo J.A., Escoté X. (2024). Polyphenols and Metabolism: From Present Knowledge to Future Challenges. J. Physiol. Biochem..

[10] Starvaggi J., Di Chio C., De Luca F., Previti S., Zappalà M., Ettari R. (2026). Selected Nutraceuticals in Metabolic Syndrome: Molecular Mechanisms and Clinical Implications. Biomedicines.

[11] Dama A., Shpati K., Daliu P., Dumur S., Gorica E., Santini A. (2024). Targeting Metabolic Diseases: The Role of Nutraceuticals in Modulating Oxidative Stress and Inflammation. Nutrients.

[12] Sun S., Liu Z., Lin M., Gao N., Wang X. (2024). Polyphenols in Health and Food Processing: Antibacterial, Anti-Inflammatory, and Antioxidant Insights. Front. Nutr..

[13] Bas T.G. (2026). Dietary Polyphenols (Flavonoids) Derived from Plants for Use in Therapeutic Health: Antioxidant Per-formance, ROS, Molecular Mechanisms, and Bioavailability Limitations. Int. J. Mol. Sci..

[14] Cano R., Bermúdez V., Galban N., Garrido B., Santeliz R., Gotera M.P., Duran P., Boscan A., Carbonell-Zabaleta A.K., Durán-Agüero S. (2024). Dietary Polyphenols and Gut Microbiota Cross-Talk: Molecular and Therapeutic Perspectives for Cardiometabolic Disease: A Narrative Review. Int. J. Mol. Sci..

[15] Green B.N., Johnson C.D., Adams A. (2006). Writing Narrative Literature Reviews for Peer-Reviewed Journals: Secrets of the Trade. J. Chiropr. Med..

[16] Ferrari R. (2015). Writing Narrative Style Literature Reviews. Med. Writ..

[17] Hall S., Leeder E. (2024). Narrative Reanalysis: A Methodological Framework for a New Brand of Reviews. Res. Synth. Methods.

[18] Page M.J., McKenzie J.E., Bossuyt P.M., Boutron I., Hoffmann T.C., Mulrow C.D., Shamseer L., Tetzlaff J.M., Akl E.A., Brennan S.E. (2021). The PRISMA 2020 Statement: An Updated Guideline for Reporting Systematic Reviews. BMJ.

[19] Vaou N., Stavropoulou E., Voidarou C., Tsakris Z., Rozos G., Tsigalou C., Bezirtzoglou E. (2022). Interactions between Medical Plant-Derived Bioactive Compounds: Focus on Antimicrobial Combination Effects. Antibiotics.

[20] Kim J.H., Land K.M., Huang C., Zhang Y.-Y. (2023). Natural Products as Drug Candidates for Redox-Related Human Disease. Pharmaceuticals.

[21] Rodriguez-Mateos A., Le Sayec M., Cheok A. (2025). Dietary (Poly)phenols and Cardiometabolic Health: From Antioxidants to Modulators of the Gut Microbiota. Proc. Nutr. Soc..

[22] Torres-Fuentes C., Schellekens H., Cryan J.F., Espín J.C., Muguerza B. (2026). Interplay between (Poly)phenols, Gut Microbiota, and Biological Rhythms: Outlook for a New Paradigm in Brain Health. Crit. Rev. Food Sci. Nutr..

[23] Liga S., Paul C., Péter F. (2023). Flavonoids: Overview of Biosynthesis, Biological Activity, and Current Extraction Techniques. Plants.

[24] Solnier J., Chang C., Pizzorno J. (2023). Consideration for Flavonoid-Containing Dietary Supplements to Tackle Deficiency and Optimize Health. Int. J. Mol. Sci..

[25] Eggersdorfer M., Wyss A. (2018). Carotenoids in Human Nutrition and Health. Arch. Biochem. Biophys..

[26] Calder P.C. (2017). Omega-3 Fatty Acids and Inflammatory Processes: From Molecules to Man. Biochem. Soc. Trans..

[27] Shahidi F., Ambigaipalan P. (2018). Omega-3 Polyunsaturated Fatty Acids and Their Health Benefits. Annu. Rev. Food Sci. Technol..

[28] Purohit K., Reddy N., Sunna A. (2024). Exploring the Potential of Bioactive Peptides: From Natural Sources to Therapeutics. Int. J. Mol. Sci..

[29] Wijesekara T., Abeyrathne E.D.N.S., Ahn D.U. (2024). Effect of Bioactive Peptides on Gut Microbiota and Their Relations to Human Health. Foods.

[30] Yang R., Jiang J., Ouyang J., Zhao Y., Xi B. (2024). Efficacy and Safety of Probiotics in Irritable Bowel Syndrome: A Systematic Review and Meta-Analysis. Clin. Nutr. ESPEN.

[31] Ding L., Duan J., Yang T., Yuan M., Ma A.H., Qin Y. (2025). Efficacy of Fermented Foods in Irritable Bowel Syndrome: A Systematic Review and Meta-Analysis of Randomized Controlled Trials. Front. Nutr..

[32] Kopczyńska J., Kowalczyk M. (2024). The Potential of Short-Chain Fatty Acid Epigenetic Regulation in Chronic Low-Grade Inflammation and Obesity. Front. Immunol..

[33] Kalkan A.E., BinMowyna M.N., Raposo A., Ahmad M.F., Ahmed F., Otayf A.Y., Carrascosa C.J., Saraiva A., Karav S. (2025). Beyond the Gut: Unveiling Butyrate’s Global Health Impact Through Gut Health and Dysbiosis-Related Conditions: A Narrative Review. Nutrients.

[34] Ruseva N., Bakalova A., Cherneva E. (2025). Functional Groups and Structural Features of Antioxidants: A Review. J. Chem. Technol. Metall..

[35] Zhang M., Liu J., Yu Y., Liu X., Shang X., Du Z., Xu M.L., Zhang T. (2024). Recent Advances in the Inhibition of Membrane Lipid Peroxidation by Food-Borne Plant Polyphenols via the Nrf2/GPx4 Pathway. J. Agric. Food Chem..

[36] Duché G., Sanderson J.M. (2024). The Chemical Reactivity of Membrane Lipids. Chem. Rev..

[37] Ghodsi A., Hidalgo A., Libreros S. (2024). Lipid Mediators in Neutrophil Biology: Inflammation, Resolution and Beyond. Curr. Opin. Hematol..

[38] Mann E.R., Lam Y.K., Uhlig H.H. (2024). Short-Chain Fatty Acids: Linking Diet, the Microbiome and Immunity. Nat. Rev. Immunol..

[39] González-Bosch C., Boorman E., Zunszain P.A., Mann G.E. (2021). Short-Chain Fatty Acids as Modulators of Redox Signaling in Health and Disease. Redox Biol..

[40] Kanojiya D.J., Mahant M., Besh S., Parekh S., Parmar G. (2025). Flavonoids: A Class of Polyphenols with Diverse Anti-inflammatory Activities and Mechanisms. Pharmacogn. Res..

[41] Chatzipieris F.P., Petsas E., Lambrinidis G., Vassiliou S., Chasapis C.T. (2026). Recent Advances in Dual COX/LOX Inhibitor Design (2020–2024): Establishing “The Rule of Four for Inflammation”. Life.

[42] Fredman G., Serhan C.N. (2024). Specialized Pro-Resolving Mediators in Vascular Inflammation and Atherosclerotic Cardiovascular Disease. Nat. Rev. Cardiol..

[43] Zhang Q., Wang Y., Zhu J., Zou M., Zhang Y., Wu H., Jin T. (2025). Specialized Pro-Resolving Lipid Mediators: A Key Player in Resolving Inflammation in Autoimmune Diseases. Sci. Bull..

[44] Kim C.H. (2023). Complex Regulatory Effects of Gut Microbial Short-Chain Fatty Acids on Immune Tolerance and Autoimmunity. Cell. Mol. Immunol..

[45] Ghorbani Z., Shoaibinobarian N., Noormohammadi M., Taylor K., Kazemi A., Bonyad A., Khoshdooz S., Löber U., Forslund-Startceva S.K. (2025). Reinforcing Gut Integrity: A Systematic Review and Meta-Analysis of Clinical Trials Assessing Probiotics, Synbiotics, and Prebiotics on Intestinal Permeability Markers. Pharmacol. Res..

[46] Liu Y., Jiao A. (2025). Flavonoids as Immunoregulators: Molecular Mechanisms in Regulating Immune Cells and Their Therapeutic Applications in Inflammatory Diseases. Front. Immunol..

[47] Santos-Sánchez G., Santos-Hernández M., Miralles B., Recio I. (2026). Bioactive Peptides: From Preclinical to Clinical Studies. Curr. Opin. Clin. Nutr. Metab. Care.

[48] Pérez-Flores J.G., García-Curiel L., Pérez-Escalante E., Contreras-López E., Aguilar-Lira G.Y., Ángel-Jijón C., González-Olivares L.G., Baena-Santillán E.S., Ocampo-Salinas I.O., Guerrero-Solano J.A. (2025). Plant Antimicrobial Compounds and Their Mechanisms of Action on Spoilage and Pathogenic Bacteria: A Bibliometric Study and Literature Review. Appl. Sci..

[49] Ngashangva N., Huidrom S., Devi I.S. (2025). Antimicrobial Peptides: Natural Templates for Next-Generation Therapeutics against Antimicrobial Resistance. Front. Cell. Infect. Microbiol..

[50] Bakkeren E., Piskovsky V., Foster K.R. (2025). Metabolic Ecology of Microbiomes: Nutrient Competition, Host Benefits, and Community Engineering. Cell Host Microbe.

[51] Ali M.S., Lee E.-B., Hsu W.-H., Suk K., Sayem S.A.J., Ullah H.M.A., Lee S.-J., Park S.-C. (2023). Probiotics and Postbiotics as an Alternative to Antibiotics: An Emphasis on Pigs. Pathogens.

[52] Nakadate K., Ito N., Kawakami K., Yamazaki N. (2025). Anti-Inflammatory Actions of Plant-Derived Compounds and Prevention of Chronic Diseases: From Molecular Mechanisms to Applications. Int. J. Mol. Sci..

[53] Biełach-Bazyluk A., Jakubowicz-Zalewska O., Myśliwiec H., Flisiak I. (2026). Specialized Pro-Resolving Lipid Mediators and Dietary Omega-3/6 Fatty Acids in Selected Inflammatory Skin Diseases: A Systematic Review. Antioxidants.

[54] Ma Z.F., Fu C., Lee Y.Y. (2025). The Modulatory Role of Bioactive Compounds in Functional Foods on Inflammation and Metabolic Pathways in Chronic Diseases. Foods.

[55] Fatima G., Khan S., Shukla V., Awaida W., Li D., Gushchina Y.S. (2025). Nutraceutical Formulations and Natural Compounds for the Management of Chronic Diseases. Front. Nutr..

[56] Martin S.S., Aday A.W., Almarzooq Z.I., Anderson C.A.M., Arora P., Avery C.L., Baker-Smith C.M., Barone Gibbs B., Beaton A.Z., Boehme A.K. (2024). Heart Disease and Stroke Statistics—2024 Update: A Report of US and Global Data from the American Heart Association. Circulation.

[57] Tasouli-Drakou V., Ogurek I., Shaikh T., Ringor M., DiCaro M.V., Lei K. (2025). Atherosclerosis: A Comprehensive Review of Molecular Factors and Mechanisms. Int. J. Mol. Sci..

[58] Liasi E., Kantilafti M., Hadjimbei E., Chrysostomou S. (2024). Monacolin K Supplementation in Patients with Hypercholesterolemia: A Systematic Review of Clinical Trials. Semergen.

[59] Blais J.E., Huang X., Zhao J.V. (2023). Overall and Sex-Specific Effect of Berberine for the Treatment of Dyslipidemia in Adults: A Systematic Review and Meta-Analysis of Randomized Placebo-Controlled Trials. Drugs.

[60] Fontané L., Pedro-Botet J., Garcia-Ribera S., Climent E., Muns M.D., Ballesta S., Satorra P., Flores-Le Roux J.A., Benaiges D. (2023). Use of Phytosterol-Fortified Foods to Improve LDL Cholesterol Levels: A Systematic Review and Meta-Analysis. Nutr. Metab. Cardiovasc. Dis..

[61] Gál R., Halmosi R., Gallyas F., Tschida M., Mutirangura P., Tóth K., Alexy T., Czopf L. (2023). Resveratrol and beyond: The Effect of Natural Polyphenols on the Cardiovascular System: A Narrative Review. Biomedicines.

[62] Sarbadhikary P., George B.P., Abrahamse H. (2021). Inhibitory Role of Berberine, an Isoquinoline Alkaloid, on NLRP3 Inflammasome Activation for the Treatment of Inflammatory Diseases. Molecules.

[63] Wu X., Wei J., Yi Y., Gong Q., Gao J. (2022). Activation of Nrf2 Signaling: A Key Molecular Mechanism of Protection against Cardiovascular Diseases by Natural Products. Front. Pharmacol..

[64] Sun Y., Zimmermann D., De Castro C.A., Actis-Goretta L. (2019). Dose-Response Relationship Between Cocoa Flavanols and Human Endothelial Function: A Systematic Review and Meta-Analysis of Randomized Trials. Food Funct..

[65] Ajoolabady A., Pratico D., Lin L., Mantzoros C.S., Bahijri S., Tuomilehto J., Ren J. (2024). Inflammation in Atherosclerosis: Pathophysiology and Therapeutic Implications. Cell Death Dis..

[66] Nesci A., Carnuccio C., Ruggieri V., D’Alessandro A., Di Giorgio A., Santoro L., Gasbarrini A., Santoliquido A., Ponziani F.R. (2023). Gut Microbiota and Cardiovascular Disease: Evidence on the Metabolic and Inflammatory Background of a Complex Relationship. Int. J. Mol. Sci..

[67] Jacobo-Velázquez D.A. (2025). Functional Foods for Cholesterol Management: A Review of the Mechanisms, Efficacy, and a Novel Cholesterol-Lowering Capacity Index. Nutrients.

[68] Wang T., Xu H., Dong R., Wu S., Guo Y., Wang D. (2023). Effectiveness of Targeting the NLRP3 Inflammasome by Using Natural Polyphenols: A Systematic Review of Implications on Health Effects. Food Res. Int..

[69] D’Unienville N.M.A., Blake H.T., Coates A.M., Hill A.M., Nelson M.J., Buckley J.D. (2021). Effect of Food Sources of Nitrate, Polyphenols, L-Arginine and L-Citrulline on Endurance Exercise Performance: A Systematic Review and Meta-Analysis of Randomized Controlled Trials. J. Int. Soc. Sports Nutr..

[70] Nouruzi S., Vasheghani Farahani A., Rezaeizadeh H., Ghafouri P., Ghorashi S.M., Omidi N. (2022). Platelet Aggregation Inhibition: An Evidence-Based Systematic Review on the Role of Herbs for Primary Prevention Based on Randomized Controlled Trials. Iran. J. Med. Sci..

[71] Golanski J., Szymanska P., Rozalski M. (2021). Effects of Omega-3 Polyunsaturated Fatty Acids and Their Metabolites on Haemostasis—Current Perspectives in Cardiovascular Disease. Int. J. Mol. Sci..

[72] Sagmeister A., Matter C.M., Stähli B.E., Scharl M. (2024). The Gut–Heart Axis: Effects of Intestinal Microbiome Modulation on Cardiovascular Disease—Ready for Therapeutic Interventions?. Int. J. Mol. Sci..

[73] Trogkanis E., Karalexi M.A., Sergentanis T.N., Kornarou E., Vassilakou T. (2024). Safety and Efficacy of the Consumption of the Nutraceutical “Red Yeast Rice Extract” for the Reduction of Hypercholesterolemia in Humans: A Systematic Review and Meta-Analysis. Nutrients.

[74] Ras R.T., Geleijnse J.M., Trautwein E.A. (2014). LDL-Cholesterol-Lowering Effect of Plant Sterols and Stanols Across Different Dose Ranges: A Meta-Analysis of Randomized Controlled Studies. Br. J. Nutr..

[75] Gholami Z., Clark C.C.T., Paknahad Z. (2024). The Effect of Psyllium on Fasting Blood Sugar, HbA1c, HOMA-IR, and Insulin Control: A GRADE-Assessed Systematic Review and Meta-Analysis of Randomized Controlled Trials. BMC Endocr. Disord..

[76] Ju J., Li J., Lin Q., Xu H. (2018). Efficacy and Safety of Berberine for Dyslipidaemias: A Systematic Review and Me-ta-Analysis of Randomized Clinical Trials. Phytomedicine.

[77] Skulas-Ray A.C., Wilson P.W.F., Harris W.S., Brinton E.A., Kris-Etherton P.M., Richter C.K., Jacobson T.A., Engler M.B., Miller M., Robinson J.G. (2019). American Heart Association Council on Arteriosclerosis, Thrombosis and Vascular Biology; Council on Lifestyle and Cardiometabolic Health; Council on Cardiovascular Disease in the Young; Council on Cardiovascular and Stroke Nursing; Council on Clinical Cardiology. Omega-3 Fatty Acids for the Management of Hypertriglyceridemia: A Science Advisory From the American Heart Association. Circulation.

[78] Malinowski B., Fajardo Leighton R.I., Hill C.G., Szandorowski P., Wiciński M. (2020). Bioactive Compounds and Their Effect on Blood Pressure—A Review. Nutrients.

[79] Shannon O.M., Mendes I., Köchl C., Mazidi M., Ashor A., Rubele S., Minihane A.-M., Mathers J.C., Siervo M. (2020). Mediterranean Diet Increases Endothelial Function in Adults: A Systematic Review and Meta-Analysis of Randomized Controlled Trials. J. Nutr..

[80] Ruscica M., Penson P.E., Ferri N., Sirtori C.R., Pirro M., Mancini G.B.J., Sattar N., Toth P.P., Sahebkar A., Lavie C.J. (2021). Impact of Nutraceuticals on Markers of Systemic Inflammation: Potential Relevance to Cardiovascular Diseases—A Position Paper from the International Lipid Expert Panel (ILEP). Prog. Cardiovasc. Dis..

[81] Miller P.E., Van Elswyk M., Alexander D.D. (2014). Long-Chain Omega-3 Fatty Acids Eicosapentaenoic Acid and Docosahexaenoic Acid and Blood Pressure: A Meta-Analysis of Randomized Controlled Trials. Am. J. Hypertens..

[82] Alexander D.D., Miller P.E., Van Elswyk M.E., Kuratko C.N., Bylsma L.C. (2017). A Meta-Analysis of Randomized Controlled Trials and Prospective Cohort Studies of Eicosapentaenoic and Docosahexaenoic Long-Chain Omega-3 Fatty Acids and Coronary Heart Disease Risk. Mayo Clin. Proc..

[83] Wan Z.-W., Zheng L.-J., Huang Y.-F., Xie J.-W., Li J.-L., Chen Y.-R. (2025). Effects of Prebiotics and Phytochemicals on Serum Trimethylamine N-Oxide Reduction and Gut Microbiota: A Systematic Review and Meta-Analysis. J. Transl. Med..

[84] Vinelli V., Biscotti P., Martini D., Del Bo’ C., Marino M., Meroño T., Nikoloudaki O., Calabrese F.M., Turroni S., Taverniti V. (2022). Effects of Dietary Fibers on Short-Chain Fatty Acids and Gut Microbiota Composition in Healthy Adults: A Systematic Review. Nutrients.

[85] Wang T., Wang Y.-Y., Shi M.-Y., Liu L. (2023). Mechanisms of Action of Natural Products on Type 2 Diabetes. World J. Diabetes.

[86] Clemente-Suárez V.J., Martín-Rodríguez A., Beltrán-Velasco A.I., Rubio-Zarapuz A., Martínez-Guardado I., Valcárcel-Martín R., Tornero-Aguilera J.F. (2025). Functional and Therapeutic Roles of Plant-Derived Antioxidants in Type 2 Diabetes Mellitus: Mechanisms, Challenges, and Considerations for Special Populations. Antioxidants.

[87] Oteng A.-B., Liu L. (2023). GPCR-Mediated Effects of Fatty Acids and Bile Acids on Glucose Homeostasis. Front. Endocrinol..

[88] Yu W., Sun S., Yan Y., Zhou H., Liu Z., Fu Q. (2025). The Role of Short-Chain Fatty Acid in Metabolic Syndrome and Its Complications: Focusing on Immunity and Inflammation. Front. Immunol..

[89] Ebrahimi R., Mohammadpour A., Medoro A., Davinelli S., Saso L., Miroliaei M. (2025). Exploring the links between polyphenols, Nrf2, and diabetes: A review. Biomed. Pharmacother..

[90] Shimu S.J., Mahir J.U.K., Shakib F.A.F., Ridoy A.A., Samir R.A., Jahan N., Hasan M.F., Sazzad S., Akter S., Mohiuddin M.S. (2025). Metabolic Reprogramming Through Polyphenol Networks: A Systems Approach to Metabolic Inflammation and Insulin Resistance. Med. Sci..

[91] Banaszak M., Górna I., Woźniak D., Przysławski J., Drzymała-Czyż S. (2024). The Impact of Curcumin, Resveratrol, and Cinnamon on Modulating Oxidative Stress and Antioxidant Activity in Type 2 Diabetes: Moving beyond an Anti-Hyperglycaemic Evaluation. Antioxidants.

[92] Shalbaf N., Sadeghi S., Homaee S., Saberian F. (2025). Probiotics, Prebiotics, Synbiotics, and FMT for Glycemic Control: A Systematic Review of Clinical Efficacy and Mechanistic Readouts in Type 2 Diabetes and Related Dysglycemia. Metab. Open.

[93] Wang X., Chen L., Zhang C., Shi Q., Zhu L., Zhao S., Luo Z., Long Y. (2024). Effect of Probiotics at Different Intervention Time on Glycemic Control in Patients with Type 2 Diabetes Mellitus: A Systematic Review and Meta-Analysis. Front. Endocrinol..

[94] Jafari A., Abbastabar M., Alaghi A., Heshmati J., Crowe F.L., Sepidarkish M. (2024). Curcumin on Human Health: A Comprehensive Systematic Review and Meta-Analysis of 103 Randomized Controlled Trials. Phytother. Res..

[95] Xie Y., Gou L., Peng M., Zheng J., Chen L. (2021). Effects of Soluble Fiber Supplementation on Glycemic Control in Adults with Type 2 Diabetes Mellitus: A Systematic Review and Meta-Analysis of Randomized Controlled Trials. Clin. Nutr..

[96] Psichas A., Sleeth M.L., Murphy K.G., Brooks L., Bewick G.A., Hanyaloglu A.C., Ghatei M.A., Bloom S.R., Frost G. (2015). The Short Chain Fatty Acid Propionate Stimulates GLP-1 and PYY Secretion via FFA2 in Rodents. Int. J. Obes..

[97] Hejazi N., Ghalandari H., Rahmanian R., Haghpanah F., Makhtoomi M., Asadi A., Askarpour M. (2024). Effects of Pro-biotics Supplementation on Glycemic Profile in Adults with Type 2 Diabetes Mellitus: A Grade-Assessed Systematic Review and Dose-Response Meta-Analysis of Randomized Controlled Trials. Clin. Nutr. ESPEN.

[98] Lan H., Su Z., Wang S. (2024). The Anti-Obesity Effects of Polyphenols: A Comprehensive Review of Molecular Mechanisms and Signal Pathways in Regulating Adipocytes. Front. Nutr..

[99] Jerab D., Blangero F., da Costa P.C.T., de Brito Alves J.L., Kefi R., Jamoussi H., Morio B., Eljaafari A. (2025). Beneficial Effects of Omega-3 Fatty Acids on Obesity and Related Metabolic and Chronic Inflammatory Diseases. Nutrients.

[100] Van Hul M., Cani P.D. (2023). The Gut Microbiota in Obesity and Weight Management: Microbes as Friends or Foe?. Nat. Rev. Endocrinol..

[101] Dias L.K.M., de Medeiros G.C.B.S., Silva A.K.N., de Araujo Morais A.H., Silva-Maia J.K. (2024). Effect of Phenolic Compounds on Intestinal Health in Preclinical Models of Diet-Induced Obesity: A Systematic Review. Food Rev. Int..

[102] Zhang Y., Balasooriya H., Sirisena S., Ng K. (2023). The Effectiveness of Dietary Polyphenols in Obesity Management: A Systematic Review and Meta-Analysis of Human Clinical Trials. Food Chem..

[103] Deehan E.C., Mocanu V., Madsen K.L. (2024). Effects of Dietary Fibre on Metabolic Health and Obesity. Nat. Rev. Gastroenterol. Hepatol..

[104] Peggion C., Calì T., Brini M. (2024). Mitochondria Dysfunction and Neuroinflammation in Neurodegeneration: Who Comes First?. Antioxidants.

[105] De Strooper B., Karran E. (2023). The Cellular Phase of Alzheimer’s Disease. Cell.

[106] Dejanovic B., Sheng M., Hanson J.E. (2024). Targeting Synapse Function and Loss for Treatment of Neurodegenerative Diseases. Nat. Rev. Drug Discov..

[107] Díaz M., de Pablo D.P., Valdés-Baizabal C., Santos G., Marin R. (2023). Molecular and Biophysical Features of Hippocampal Lipid Raft Aging are Modified by Dietary n-3 Long-Chain Polyunsaturated Fatty Acids. Aging Cell.

[108] Beyer M.P., Videla L.A., Farías C., Valenzuela R. (2023). Potential Clinical Applications of Pro-Resolving Lipids Mediators from Docosahexaenoic Acid. Nutrients.

[109] Sidiropoulou G.A., Metaxas A., Kourti M. (2023). Natural Antioxidants that Act against Alzheimer’s Disease through Modulation of the NRF2 Pathway: A Focus on their Molecular Mechanisms of Action. Front. Endocrinol..

[110] Ross F.C., Mayer D.E., Horn J., Cryan J.F., Del Rio D., Randolph E., Gill C.I.R., Gupta A., Ross R.P., Stanton C. (2024). Potential of Dietary Polyphenols for Protection from Age-Related Decline and Neurodegeneration: A Role for Gut Microbiota?. Nutr. Neurosci..

[111] Liu M., Lu Y., Xue G., Han L., Jia H., Wang Z., Zhang J., Liu P., Yang C., Zhou Y. (2024). Role of Short-Chain Fatty Acids in Host Physiology. Anim. Model. Exp. Med..

[112] Salminen A. (2023). Activation of Aryl Hydrocarbon Receptor (AhR) in Alzheimer’s Disease: Role of Tryptophan Metabolites Generated by Gut Host–Microbiota. J. Mol. Med..

[113] Zupo R., Castellana F., Panza F., Solfrizzi V., Lozupone M., Tardugno R., Cicero N., Corbo F., Crupi P., Sardone R. (2025). Alzheimer’s Disease May Benefit from Olive Oil Polyphenols: A Systematic Review on Preclinical Evidence Supporting the Effect of Oleocanthal on Amyloid-β Load. Curr. Neuropharmacol..

[114] Mett J. (2021). The Impact of Medium Chain and Polyunsaturated ω-3-Fatty Acids on Amyloid-β Deposition, Oxidative Stress and Metabolic Dysfunction Associated with Alzheimer’s Disease. Antioxidants.

[115] Kim S.B., Ryu H.Y., Nam W., Lee S.M., Jang M.R., Kwak Y.G., Kang G.I., Song K.S., Lee J.W. (2023). The Neuroprotective Effects of Dendropanax morbifera Water Extract on Scopolamine-Induced Memory Impairment in Mice. Int. J. Mol. Sci..

[116] Calderon Martinez E.V., Saji S.Z., Ore J.V.S., Borges-Sosa O.A., Srinivas S., Mareddy N.S.R., Manzoor T., Di Vanna M., Al Shanableh Y., Taneja R. (2024). The Effects of Omega-3, DHA, EPA, Souvenaid^®^ in Alzheimer’s Disease: A Systematic Review and Meta-Analysis. Neuropsychopharmacol. Rep..

[117] Xiang L., Wang Y., Liu S., Liu B., Jin X., Cao X. (2023). Targeting Protein Aggregates with Natural Products: An Optional Strategy for Neurodegenerative Diseases. Int. J. Mol. Sci..

[118] Moosavi F., Hosseini R., Saso L., Firuzi O. (2016). Modulation of neurotrophic signaling pathways by polyphenols. Drug Des. Dev. Ther..

[119] Ziaei S., Mohammadi S., Hasani M., Morvaridi M., Belančić A., Daneshzad E., Saleh S.A.K., Adly H.M., Heshmati J. (2024). A Systematic Review and Meta-Analysis of the Omega-3 Fatty Acids Effects on Brain-Derived Neurotrophic Factor (BDNF). Nutr. Neurosci..

[120] Jie S., Fu A., Wang C., Rajabi S. (2025). A Comprehensive Review on the Impact of Polyphenol Supplementation and Exercise on Depression and Brain Function Parameters. Behav. Brain Funct..

[121] Kaluza M., Ksiazek-Winiarek D., Szpakowski P., Czpakowska J., Fijalkowska J., Glabinski A. (2025). Polyphenols in the Central Nervous System: Cellular Effects and Liposomal Delivery Approaches. Int. J. Mol. Sci..

[122] Tanaka M., Araujo A.C., Valenti V.E., Guiguer E.L., Catharin V.C.S., Gualhardi C.M., Pereira E.S.B.M., Goulart R.A., Haber R.S.A., de Carvalho A.C.A. (2026). From Polyphenols to Prodrugs: Bridging the Blood–Brain Barrier with Nanomedicine and Neurotherapeutics. Int. J. Mol. Sci..

[123] Shen L., Dettmer U. (2024). Alpha-Synuclein Effects on Mitochondrial Quality Control in Parkinson’s Disease. Biomolecules.

[124] Macedo V.F.B., Macedo V.K.B., de Miranda Coelho J.A.P., de Melo Barboza A.M. (2025). Parkinson’s Disease Physiopathology—Beyond the α-Synuclein Aggregation: A Narrative Review. Front. Aging Neurosci..

[125] Coukos R., Krainc D. (2024). Key Genes and Convergent Pathogenic Mechanisms in Parkinson Disease. Nat. Rev. Neurosci..

[126] Alves B.D.S., Schimith L.E., da Cunha A.B., Dora C.L., Hort M.A. (2024). Omega-3 Polyunsaturated Fatty Acids and Parkinson’s Disease: A Systematic Review of Animal Studies. J. Neurochem..

[127] Pechlivani L., Giannakis A., Sioka C., Alexiou G.A., Konitsiotis S., Kyritsis A.P. (2025). Therapeutic Potential of Flavonoids in Parkinson’s Disease. J. Nutr..

[128] Kalyanaraman B., Cheng G., Hardy M. (2024). Gut Microbiome, Short-Chain Fatty Acids, α-Synuclein, Neuroinflammation, and ROS/RNS: Relevance to Parkinson’s Disease and Therapeutic Implications. Redox Biol..

[129] Iwaniak P., Owe-Larsson M., Urbańska E.M. (2024). Microbiota, Tryptophan and Aryl Hydrocarbon Receptors as the Target Triad in Parkinson’s Disease—A Narrative Review. Int. J. Mol. Sci..

[130] Chen W., Fan T., Xu R. (2025). Diet for the Prevention and Treatment of Parkinson’s Disease. Front. Nutr..

[131] Akhlaghi M., Foshati S., Hashemi Moghaddam F., Sasani M.R., Kazemi A. (2025). Foods and Dietary Intakes and the Risk of Parkinson’s Disease: A Systematic Review and Dose–Response Meta-analysis of Prospective Cohort Studies. Nutr. Rev..

[132] Anwar L., Ahmad E., Imtiaz M., Ahmad M., Aziz M.F., Ibad T. (2024). The Impact of Diet on Parkinson’s Disease: A Systematic Review. Cureus.

[133] Dagogo-Jack I., Shaw A.T. (2018). Tumour Heterogeneity and Resistance to Cancer Therapies. Nat. Rev. Clin. Oncol..

[134] Hanahan D. (2022). Hallmarks of Cancer: New Dimensions. Cancer Discov..

[135] Cháirez-Ramírez M.H., de la Cruz-López K.G., García-Carrancá A. (2021). Polyphenols as Antitumor Agents Targeting Key Players in Cancer-Driving Signaling Pathways. Front. Pharmacol..

[136] Torres W., Pérez J.L., Díaz M.P., D’Marco L., Checa-Ros A., Carrasquero R., Angarita L., Gómez Y., Chacín M., Ramírez P. (2023). The Role of Specialized Pro-Resolving Lipid Mediators in Inflammation-Induced Carcinogenesis. Int. J. Mol. Sci..

[137] Ponte L.G.S., Pavan I.C.B., Mancini M.C.S., da Silva L.G.S., Morelli A.P., Severino M.B., Bezerra R.M.N., Simabuco F.M. (2021). The Hallmarks of Flavonoids in Cancer. Molecules.

[138] Luo J., Peng S., Jiang Z., Wang Q., Zhang M., Zeng Y., Yuan Y., Xia M., Hong Z., Yan Y. (2024). Roles and Therapeutic Opportunities of ω-3 Long-Chain Polyunsaturated Fatty Acids in Lung Cancer. iScience.

[139] Kalyanaraman B., Cheng G., Hardy M. (2024). The Role of Short-Chain Fatty Acids in Cancer Prevention and Cancer Treatment. Arch. Biochem. Biophys..

[140] Molfino A., Imbimbo G., Gallicchio C., Muscaritoli M. (2024). Tryptophan Metabolism and Kynurenine Metabolites in Cancer: Systemic Nutritional and Metabolic Implications. Curr. Opin. Clin. Nutr. Metab. Care.

[141] Hegde M.M., Lakshman K. (2023). Role of Polyphenols and Flavonoids as Anti-Cancer Drug Candidates: A Review. Pharmacogn. Res..

[142] Kiyasu Y., Zuo X., Liu Y., Yao J.C., Shureiqi I. (2024). EPA, DHA, and Resolvin Effects on Cancer Risk: The Underexplored Mechanisms. Prostaglandins Other Lipid Mediat..

[143] Gomes S., Rodrigues A.C., Pazienza V., Preto A. (2023). Modulation of the Tumor Microenvironment by Microbiota-Derived Short-Chain Fatty Acids: Impact in Colorectal Cancer Therapy. Int. J. Mol. Sci..

[144] An X., Yu W., Liu J., Tang D., Yang L., Chen X. (2024). Oxidative Cell Death in Cancer: Mechanisms and Therapeutic Opportunities. Cell Death Dis..

[145] Jiang Z., Gu Z., Yu X., Cheng T., Liu B. (2024). Research Progress on the Role of Bypass Activation Mechanisms in Resistance to Tyrosine Kinase Inhibitors in Non-Small Cell Lung Cancer. Front. Oncol..

[146] Fu Y.-C., Liang S.-B., Luo M., Wang X.-P. (2025). Intratumoral Heterogeneity and Drug Resistance in Cancer. Cancer Cell Int..

[147] Song P., Song F., Shao T., Wang P., Li R., Chen Z.-S., Zhang Z., Xue G. (2024). Natural Products: Promising Therapeutics for Targeting Regulatory Immune Cells in the Tumor Microenvironment. Front. Pharmacol..

[148] Xiang Y., Du A., Wang Z., Pan H., Yuan K. (2026). Short-Chain Fatty Acids in the Tumor Microenvironment: From Molecular Mechanisms to Cancer Therapy. Theranostics.

[149] Zhao Y., Chen J., Qin Y., Yuan J., Yu Z., Ma R., Liu F., Zhao J. (2025). Linking Short-Chain Fatty Acids to Systemic Homeostasis: Mechanisms, Therapeutic Potential, and Future Directions. J. Nutr. Metab..

[150] Maldonado-Salinas R., Caballero-Salazar S., Castillejos-López M., Aquino-Gálvez A., Velasco-Hidalgo L., García-Guzmán A., Pliego-Villanueva C., Alavez-Pérez N.S., Montesinos-Correa H., Torres-Espíndola L.M. (2025). Omega-3 Fatty Acids and Chemotherapy-Induced Toxicities: Mechanisms and Emerging Evidence with a Pediatric Focus. Nutr. Metab..

[151] Chidananda C., Thakur G., Datta D., Popat K. (2025). A Comprehensive Review of Alkaloids in Cancer Therapy: Focusing on Molecular Mechanisms and Synergistic Potential of Piperine in Colorectal Cancer. 3 Biotech..

[152] Cecerska-Heryć E., Wiśniewska Z., Serwin N., Polikowska A., Goszka M., Engwert W., Michałów J., Pękała M., Budkowska M., Michalczyk A. (2024). Can Compounds of Natural Origin Be Important in Chemoprevention? Anticancer Properties of Quercetin, Resveratrol, and Curcumin—A Comprehensive Review. Int. J. Mol. Sci..

[153] Sun J., Chen S., Zang D., Sun H., Sun Y., Chen J. (2024). Butyrate as a promising therapeutic target in cancer: From pathogenesis to clinic. Int. J. Oncol..

[154] Wang J., Zhao Q., Zhang S., Liu J., Fan X., Han B., Hou Y., Ai X. (2026). Microbial short-chain fatty acids: Effective histone deacetylase inhibitors in immune regulation (review). Int. J. Mol. Med..

[155] Yang W., Cong Y. (2021). Gut Microbiota-Derived Metabolites in the Regulation of Host Immune Responses and Immune-Related Inflammatory Diseases. Cell. Mol. Immunol..

[156] Kumar S., Gupta S. (2021). Dietary Phytochemicals and their Role in Cancer Chemoprevention. J. Cancer Metastasis Treat..

[157] Zhang S., Mao B., Cui S., Zhang Q., Zhao J., Tang X., Chen W. (2024). Absorption, Metabolism, Bioactivity, and Biotransformation of Epigallocatechin Gallate. Crit. Rev. Food Sci. Nutr..

[158] Almatroodi S.A., Alsahli M.A., Rahmani A.H. (2022). Berberine: An Important Emphasis on Its Anticancer Effects through Modulation of Various Cell Signaling Pathways. Molecules.

[159] Mcluskie A., Bowers M., Bayly J., Yule M.S., Maddocks M., Fallon M., Skipworth R.J., Laird B.J.A. (2025). Nutritional Interventions in Randomized Clinical Trials for People with Incurable Solid Cancer: A Systematic Review. Clin. Nutr..

[160] Ilerhunmwuwa N.P., Abdul Khader A.H.S., Smith C., Cliff E.R.S., Booth C.M., Hottel E., Aziz M., Lee-Smith W., Goodman A., Chakraborty R. (2024). Dietary Interventions in Cancer: A Systematic Review of all Randomized Controlled Trials. J. Natl. Cancer Inst..

[161] Gutsche L.C., Dörfler J., Hübner J. (2025). Curcumin as a Complementary Treatment in Oncological Therapy: A Systematic Review. Eur. J. Clin. Pharmacol..

[162] Parada Venegas D., De la Fuente M.K., Landskron G., González M.J., Quera R., Dijkstra G., Harmsen H.J.M., Faber K.N., Hermoso M.A. (2019). Short Chain Fatty Acids (SCFAs)-Mediated Gut Epithelial and Immune Regulation and Its Relevance for Inflammatory Bowel Diseases. Front. Immunol..

[163] Prajapati S.K., Yadav D., Katiyar S., Jain S., Yadav H. (2025). Postbiotics as Mitochondrial Modulators in Inflammatory Bowel Disease: Mechanistic Insights and Therapeutic Potential. Biomolecules.

[164] Hindle V.K., Veasley N.M., Holscher H.D. (2025). Microbiota-Focused Dietary Approaches to Support Health: A Systematic Review. J. Nutr..

[165] Maghsoumi-Norouzabad L., Bagherzadeh-Karimi A., Aliakbari Majd S., Hosseini L., Shahi F. (2025). The Effects of Prebiotic Dietary Fibers, Probiotics, and Synbiotics on Gut Permeability and Immunity: A Systematic Review. Iran. J. Med. Sci..

[166] Ding G., Yang X., Li Y., Wang Y., Du Y., Wang M., Ye R., Wang J., Zhang Y., Chen Y. (2025). Gut Microbiota Regulates Gut Homeostasis, Mucosal Immunity and Influences Immune-Related Diseases. Mol. Cell. Biochem..

[167] Du Y., He C., An Y., Huang Y., Zhang H., Fu W., Wang M., Shan Z., Xie J., Yang Y. (2024). The Role of Short Chain Fatty Acids in Inflammation and Body Health. Int. J. Mol. Sci..

[168] Ahn S.-I., Cho S., Jeon E., Park M., Chae B., Ditengou I.C.P., Choi N.-J. (2022). The Effect of Probiotics on Intestinal Tight Junction Protein Expression in Animal Models: A Meta-Analysis. Appl. Sci..

[169] Dmytriv T.R., Storey K.B., Lushchak V.I. (2024). Intestinal Barrier Permeability: The Influence of Gut Microbiota, Nutrition, and Exercise. Front. Physiol..

[170] Ran L., Li C., Wang P., Tang J., Qu Z., Hao Y., Zhang Y. (2026). Aryl Hydrocarbon Receptor: A Potential Target for Natural Products in the Treatment of Inflammatory Bowel Disease. Front. Immunol..

[171] Cui X., Qiu J., Xu Y., Guan Z., Ding Y., Yu S., Fan S., Song G., Ren X. (2026). Mechanisms by Which Tryptophan Metabolites Enhance Intestinal Barrier Function to Prevent Necrotizing Enterocolitis in Preterm Infants. Front. Immunol..

[172] Lei Y., Sun X., Ruan T., Lu W., Deng B., Zhou R., Mu D. (2025). Effects of Probiotics and Diet Management in Patients with Irritable Bowel Syndrome: A Systematic Review and Network Meta-Analysis. Nutr. Rev..

[173] Liu W., Zhang S., Dong C., Lv X., Zheng X., Zhao W., Jamali M., Abedi R., Saedisomeolia A. (2025). Probiotics and Inflammatory Bowel Disease: An Umbrella Meta-Analysis of Relapse, Recurrence, and Remission Outcomes. Nutr. Metab..

[174] Zheng Q., Li C., Yang L., Qiu Y. (2025). Efficacy and Safety of Probiotics as Adjuvant Treatment for Crohn’s Disease: A Meta-Analysis of Randomized Controlled Trials. Curr. Res. Food Sci..

[175] Sanz Y., Cryan J.F., Deschasaux-Tanguy M., Elinav E., Lambrecht R., Veiga P. (2025). The Gut Microbiome Connects Nutrition and Human Health. Nat. Rev. Gastroenterol. Hepatol..

[176] Smolinska S., Popescu F.-D., Zemelka-Wiacek M. (2025). A Review of the Influence of Prebiotics, Probiotics, Synbiotics, and Postbiotics on the Human Gut Microbiome and Intestinal Integrity. J. Clin. Med..

[177] Zeng L., Qian Y., Cui X., Zhao J., Ning Z., Cha J., Wang K., Ge C., Jia J., Dou T. (2025). Immunomodulatory Role of Gut Microbial Metabolites: Mechanistic Insights and Therapeutic Frontiers. Front. Microbiol..

[178] Quinn-Bohmann N., Carr A.V., Gibbons S.M. (2025). Metabolic Modeling Reveals Determinants of Prebiotic and Probiotic Treatment Efficacy across Two Human Intervention Trials. medRxiv.

[179] Kamel M., Aleya S., Alsubih M., Aleya L. (2024). Microbiome Dynamics: A Paradigm Shift in Combatting Infectious Diseases. J. Pers. Med..

[180] Seo H., Kim S., Beck S., Song H.-Y. (2024). Perspectives on Microbiome Therapeutics in Infectious Diseases: A Comprehensive Approach Beyond Immunology and Microbiology. Cells.

[181] Jones K., de Brito C.B., Byndloss M.X. (2025). Metabolic Tug-of-War: Microbial Metabolism Shapes Colonization Resistance against Enteric Pathogens. Cell Chem. Biol..

[182] De Rossi L., Rocchetti G., Lucini L., Rebecchi A. (2025). Antimicrobial Potential of Polyphenols: Mechanisms of Action and Microbial Responses—A Narrative Review. Antioxidants.

[183] Siriphap A., Kiddee A., Duangjai A., Yosboonruang A., Pook-In G., Saokaew S., Sutheinkul O., Rawangkan A. (2022). Antimicrobial Activity of the Green Tea Polyphenol (−)-Epigallocatechin-3-Gallate (EGCG) against Clinical Isolates of Multidrug-Resistant Vibrio cholerae. Antibiotics.

[184] Davidova S., Galabov A.S., Satchanska G. (2024). Antibacterial, Antifungal, Antiviral Activity, and Mechanisms of Action of Plant Polyphenols. Microorganisms.

[185] Huang J., Zaynab M., Sharif Y., Khan J., Al-Yahyai R., Sadder M., Ali M., Alarab S.R., Li S. (2024). Tannins as Antimicrobial Agents: Understanding Toxic Effects on Pathogens. Toxicon.

[186] Cruz A.S.d.P., de Sousa T.C., Kruklis N.E., Neto N.A.d.S., Lira B.O.D.V., de Andrade G.R., Franco O.L., Brand G.D., Ramada M.H.S. (2026). Synergistic Potential of Plant Alkaloids and Intragenic Antimicrobial Peptides in Treating Multidrug-Resistant Infectious Diseases. Antibiotics.

[187] Basil M.C., Levy B.D. (2016). Specialized Pro-Resolving Mediators: Endogenous Regulators of Infection and Inflammation. Nat. Rev. Immunol..

[188] Estevinho M.M., Yuan Y., Rodríguez-Lago I., Sousa-Pimenta M., Dias C.C., Barreiro-de Acosta M., Jairath V., Magro F. (2024). Efficacy and Safety of Probiotics in IBD: An Overview of Systematic Reviews and Updated Meta-Analysis of Randomized Controlled Trials. United Eur. Gastroenterol. J..

[189] Hashim N.T., Babiker R., Padmanabhan V., Islam M.S., Mohammed R., Priya S.P., Chaitanya N.C.S.K., Parveen Dasnadi S., Ahmed A., Gobara Gismalla B. (2025). Polyphenolic Compounds in Combating MDR Periodontal Pathogens: Current Research and Future Directions. Front. Pharmacol..

[190] Yang X., Wang Y., Li L., Tang D., Yan Z., Li M., Jiang J., Bi D. (2025). Berberine and Its Nanoformulations and Extracts: Potential Strategies and Future Perspectives against Multi-Drug Resistant Bacterial Infections. Front. Microbiol..

[191] Serhan C.N., Chiang N., Nshimiyimana R. (2024). Low-Dose Pro-Resolving Mediators Temporally Reset the Resolution Response to Microbial Inflammation. Mol. Med..

[192] Kris-Etherton P.M., Petersen K.S., LaMarche B., Karmally W., Guyton J.R., Champagne C., Lichtenstein A.H., Bray G.A., Sacks F.M., Maki K.C. (2025). The Role of Nutrition-Related Clinical Trials in Informing Dietary Recommendations for Health and Treatment of Diseases. J. Clin. Lipidol..

[193] Wallach J.D., Yoon S., Doernberg H., Glick L.R., Ciani O., Taylor R.S., Mooghali M., Ramachandran R., Ross J.S. (2024). Associations Between Surrogate Markers and Clinical Outcomes for Nononcologic Chronic Disease Treatments. JAMA.

[194] Djuricic I., Calder P.C. (2025). N-3 Fatty Acids (EPA and DHA) and Cardiovascular Health—Updated Review of Mechanisms and Clinical Outcomes. Curr. Atheroscler. Rep..

[195] Li P., Wang Q., Chen K., Zou S., Shu S., Lu C., Wang S., Jiang Y., Fan C., Luo Y. (2022). Red Yeast Rice for Hyperlipidemia: A Meta-Analysis of 15 High-Quality Randomized Controlled Trials. Front. Pharmacol..

[196] Arisi T.O.P., da Silva D.S., Stein E., Weschenfelder C., de Oliveira P.C., Marcadenti A., Lehnen A.M., Waclawovsky G. (2024). Effects of Cocoa Consumption on Cardiometabolic Risk Markers: Meta-Analysis of Randomized Controlled Trials. Nutrients.

[197] Behrouz V., Zahroodi M., Clark C.C.T., Mir E., Atashi N., Rivaz R. (2026). Effects of Garlic Supplementation on Cardiovascular Risk Factors in Adults: A Comprehensive Updated Systematic Review and Meta-Analysis of Randomized Controlled Trials. Nutr. Rev..

[198] Gutierrez-Mariscal F.M., de la Cruz-Ares S., Torres-Peña J.D., Alcalá-Diaz J.F., Yubero-Serrano E.M., López-Miranda J. (2021). Coenzyme Q10 and Cardiovascular Diseases. Antioxidants.

[199] Potì F., Santi D., Spaggiari G., Zimetti F., Zanotti I. (2019). Polyphenol Health Effects on Cardiovascular and Neurodegenerative Disorders: A Review and Meta-Analysis. Int. J. Mol. Sci..

[200] Estruch R., Ros E., Salas-Salvadó J., Covas M.I., Corella D., Arós F., Gómez-Gracia E., Ruiz-Gutiérrez V., Fiol M., Lapetra J. (2018). PREDIMED Study Investigators. Primary Prevention of Cardiovascular Disease with a Mediterranean Diet Supplemented with Extra-Virgin Olive Oil or Nuts. N. Engl. J. Med..

[201] Liu D., Zhao H., Zhang Y., Hu J., Xu H. (2025). Efficacy and Safety of Berberine on the Components of Metabolic Syndrome: A Systematic Review and Meta-Analysis of Randomized Placebo-Controlled Trials. Front. Pharmacol..

[202] Reimer R.A., Theis S., Zanzer Y.C. (2024). The Effects of Chicory Inulin-Type Fructans Supplementation on Weight Management Outcomes: Systematic Review, Meta-Analysis, and Meta-Regression of Randomized Controlled Trials. Am. J. Clin. Nutr..

[203] Farhat G. (2024). Polyphenols in Obesity and Weight Management: Are They Worth Further Research? An Umbrella Review. Nutr. Bull..

[204] Bineid M.M., Liu L., Ventura E.F., Bansal S., Curi-Quinto K., Del Valle-Mendoza J., Walton G.E., Vimaleswaran K.S. (2025). The Effect of Probiotics, Prebiotics and Synbiotics on Gut Microbial Community Profile in Overweight and Obese Latin American and Caribbean Populations: A Systematic Review of Human Trials. Gut Microbiome.

[205] Baroni I., Fabrizi D., Luciani M., Magon A., Conte G., De Angeli G., Paglione G., Ausili D., Caruso R. (2024). Probiotics and Synbiotics for Glycemic Control in Diabetes: A Systematic Review and Meta-Analysis of Randomized Controlled Trials. Clin. Nutr..

[206] Virk J.P., Fernando M.G., Asih P.R., Martins R.N. (2026). Translational Feasibility of Curcumin for Treatment of Alzheimer’s Disease: A Critical Appraisal of Clinical Challenges. Antioxidants.

[207] Godos J., Micek A., Mena P., Del Rio D., Galvano F., Castellano S., Grosso G. (2024). Dietary (Poly)phenols and Cognitive Decline: A Systematic Review and Meta-Analysis of Observational Studies. Mol. Nutr. Food Res..

[208] Hu J., Guo Y., Ren L., Zhang H., Qin X., Yin F., Liu X. (2026). Natural Products Targeting the PI3K/Akt/mTOR-Mediated Autophagy Pathway in Cancer Therapy: Recent Advances and Clinical Perspectives. J. Nat. Prod..

[209] Singla R.K., Wang X., Gundamaraju R., Joon S., Tsagkaris C., Behzad S., Khan J., Gautam R., Goyal R., Rakmai J. (2023). Natural Products Derived from Medicinal Plants and Microbes Might Act as a Game-Changer in Breast Cancer: A Comprehensive Review of Preclinical and Clinical Studies. Crit. Rev. Food Sci. Nutr..

[210] Wali A.F., Talath S., Babiker R., El-Tanani M., Rangraze I.R., Ibraheem W., Al Aldhaheri Y., Satyam S.M., El-Tanani Y. (2026). Natural Products as Kinase Inhibitors in Lung Cancer: Molecular Mechanisms, Therapeutic Potential, and Clinical Trials. Front. Pharmacol..

[211] Deng R., Zong G.F., Wang X., Yue B.J., Cheng P., Tao R.Z., Li X., Wei Z.H., Lu Y. (2025). Promises of Natural Products as Clinical Applications for Cancer. Biochim. Biophys. Acta (BBA) -Rev. Cancer.

[212] Hijová E. (2025). Probiotics in IBD: Evidence and Perspectives on Patient Health and Disease Management. Int. J. Mol. Sci..

[213] Ballena-Caicedo J., Zuzunaga-Montoya F.E., Acosta-Porzoliz R., García-Ahumada F., Rivera-Lozada O., Valladares-Garrido M.J., Vera-Ponce V.J. (2026). Probiotics in Irritable Bowel Syndrome: An Umbrella Review of 27 Systematic Reviews on Methodological Quality and Certainty of Evidence. J. Clin. Med..

[214] Mohseni S., Tavakoli A., Ghazipoor H., Pouralimohamadi N., Zare R., Rampp T., Shayesteh M., Pasalar M. (2025). Curcumin for the Clinical Treatment of Inflammatory Bowel Diseases: A Systematic Review and Meta-Analysis of Placebo-Controlled Randomized Clinical Trials. Front. Nutr..

[215] Bertin L., Facchin S., Barberio B., Maniero D., Lorenzon G., Cesaroni F., Zanconato M., Romanelli G., Francini-Pesenti F., Busetto L. (2026). Diet and Gut Microbiota in Inflammatory Bowel Disease: A Clinical and Nutritional Perspective. Pharmaceuticals.

[216] Peng Z., Li D., Wu N., Wang X.Y., Sun G.X., Gao H.B., Li H.X. (2025). Safety and Efficacy of Curcumin in the Treatment of Ulcerative Colitis: An Updated Systematic Review and Meta-Analysis of Randomized Controlled Trials. Explore.

[217] Esmaeilinezhad Z., Ghosh N.R., Walsh C.M., Steen J.P., Burgman A.M., Mertz D., Johnston B.C. (2025). Probiotics for the Prevention of Clostridioides difficile-Associated Diarrhea in Adults and Children. Cochrane Database Syst. Rev..

[218] Goodman C., Keating G., Georgousopoulou E., Hespe C., Levett K. (2021). Probiotics for the Prevention of Antibiotic-Associated Diarrhoea: A Systematic Review and Meta-Analysis. BMJ Open.

[219] Rau S., Gregg A., Yaceczko S., Limketkai B. (2024). Prebiotics and Probiotics for Gastrointestinal Disorders. Nutrients.

[220] Moračanin S.V., Danilović B., Milijašević M., Milijašević J.B., Tambur Z., Moračanin M. (2025). Probiotics, Prebiotics and Synbiotics for Combating Antimicrobial Resistance in the Food Chain. Processes.

[221] Zhang Y., Xu Y., Hu L., Wang X. (2025). Advancements Related to Probiotics for Preventing and Treating Recurrent Respiratory Tract Infections in Children. Front. Pediatr..

[222] Bettocchi S., Comotti A., Elli M., De Cosmi V., Berti C., Alberti I., Mazzocchi A., Rosazza C., Agostoni C., Milani G.P. (2025). Probiotics and Fever Duration in Children With Upper Respiratory Tract Infections: A Randomized Clinical Trial. JAMA Netw. Open.

[223] Sharma V., Sharma D., Saini M., Jain A., Chaudhary J., Kaur N., Sahoo S., Singh S.K., Goyal K., Rekha A. (2025). Flavonoids as Antimicrobial Agents: A Comprehensive Review of Mechanisms and Therapeutic Potential. Curr. Pharm. Biotechnol..

[224] Mandal M.K., Domb A.J. (2024). Antimicrobial Activities of Natural Bioactive Polyphenols. Pharmaceutics.

[225] Zhou H., Wang W., Cai L., Yang T. (2023). Potentiation and Mechanism of Berberine as an Antibiotic Adjuvant Against Multidrug-Resistant Bacteria. Infect. Drug Resist..

[226] Duda-Madej A., Viscardi S., Bazan H., Sobieraj J. (2025). Exploring the Role of Berberine as a Molecular Disruptor in Antimicrobial Strategies. Pharmaceuticals.

[227] Vomero M., Lamberti L., Corberi E., Currado D., Marino A., Berardicurti O., Fava M., Leuti A., Maccarrone M., Giacomelli R. (2025). Specialized Pro-Resolving Mediators and Autoimmunity: Recent Insights and Future Perspectives. Autoimmun. Rev..

[228] Favari C., Rinaldi de Alvarenga J.F., Sánchez-Martínez L., Tosi N., Mignogna C., Cremonini E., Manach C., Bresciani L., Del Rio D., Mena P. (2024). Factors Driving the Inter-Individual Variability in the Metabolism and Bioavailability of (Poly)phenolic Metabolites: A Systematic Review of Human Studies. Redox Biol..

[229] Cui Y., Zhou Q., Jin M., Jiang S., Shang P., Dong X., Li L. (2024). Research Progress on Pharmacological Effects and Bioavailability of Berberine. Naunyn-Schmiedeberg’s Arch. Pharmacol..

[230] Murray M. (2024). Omega-3 Polyunsaturated Fatty Acid Derived Lipid Mediators: A Comprehensive Update on Their Application in Anti-Cancer Drug Discovery. Expert Opin. Drug Discov..

[231] Grant J.K., Dangl M., Ndumele C.E., Michos E.D., Martin S.S. (2024). A Historical, Evidence-Based, and Narrative Review on Commonly Used Dietary Supplements in Lipid-Lowering. J. Lipid Res..

[232] El-Saadony M.T., Saad A.M., Korma S.A., Salem H.M., Abd El-Mageed T.A., Alkafaas S.S., Elsalahaty M.I., Elkafas S.S., Mosa W.F.A., Ahmed A.E. (2024). Garlic Bioactive Substances and Their Therapeutic Applications for Improving Human Health: A Comprehensive Review. Front. Immunol..

[233] Nyulas K.I., Simon-Szabó Z., Pál S., Fodor M.A., Dénes L., Cseh M.J., Barabás-Hajdu E., Csipor B., Szakács J., Preg Z. (2024). Cardiovascular Effects of Herbal Products and Their Interaction with Antihypertensive Drugs—Comprehensive Review. Int. J. Mol. Sci..

[234] El-Saadony M.T., Saad A.M., Mohammed D.M., Alkafaas S.S., Ghosh S., Negm S.H., Salem H.M., Fahmy M.A., Mosa W.F.A., Ibrahim E.H. (2025). Curcumin, an Active Component of Turmeric: Biological Activities, Nutritional Aspects, Immunological, Bioavailability, and Human Health Benefits—A Comprehensive Review. Front. Immunol..

[235] El-Saadony M.T., Saad A.M., Sitohy M., Alkafaas S.S., Dladla M., Ghosh S., Mohammed D.M., Ibrahim E.H., Fahmy M.A., Elkelish A. (2026). Probiotics and Human Health: Biological Activities, Nutritional Aspects, Immunomodulatory Properties, Applications, and Future Perspectives—A Comprehensive Review. Front. Immunol..

[236] Chatsirisakul O., Leenabanchong N., Siripaopradit Y., Chang C.-W., Buhngamongkol P., Pongpirul K. (2025). Strain-Specific Therapeutic Potential of Lactiplantibacillus plantarum: A Systematic Scoping Review. Nutrients.

[237] Morand C. (2024). How to Better Consider and Understand Interindividual Variability in Response to Polyphenols in Clinical Trials. Front. Nutr..

[238] Evans M., Lewis E.D., Antony J.M., Crowley D.C., Guthrie N., Blumberg J.B. (2022). Breaking New Frontiers: Assessment and Re-Evaluation of Clinical Trial Design for Nutraceuticals. Front. Nutr..

[239] Schiffmann R. (2025). Role of Biomarkers in Diagnosing Disease, Assessing the Severity and Progression of Disease, and Evaluating the Efficacy of Therapies. J. Inherit. Metab. Dis..

[240] Healey G.R., Murphy R., Brough L., Butts C.A., Coad J. (2017). Interindividual Variability in Gut Microbiota and Host Response to Dietary Interventions. Nutr. Rev..

[241] Marx W., Visser M., Wallace C., Jacka F.N., Bayes J., Francis H., Opie R., Hockey M., Teasdale S.B., Sanchez Villegas A. (2025). Methodological and Reporting Recommendations for Clinical Trials in Nutritional Psychiatry: Guidelines from the International Society for Nutritional Psychiatry Research. Br. J. Nutr..

[242] Fogaça A.L., Ostolin T.L.V.D.P., Pagano R., Santana A.B.N., Fonseca D.C., Oliveira L.T.D., Alves B.S., Bersch-Ferreira Â.C. (2026). A Standardized Framework for Dietary Intake Data: Implementation and Monitoring of the 24-Hour Dietary Recalls in the PROVEN-DIA Trial. Front. Nutr..

[243] Hernández-Lorca M., Timón I.M., Ballester P., Henarejos-Escudero P., García-Muñoz A.M., Victoria-Montesinos D., Barcina-Pérez P. (2025). Dietary Modulation of CYP3A4 and Its Impact on Statins and Antidiabetic Drugs: A Narrative Review. Pharmaceuticals.

[244] Javaid M., Kadhim K., Bawamia B., Cartlidge T., Farag M., Alkhalil M. (2024). Bleeding Risk in Patients Receiving Omega-3 Polyunsaturated Fatty Acids: A Systematic Review and Meta-Analysis of Randomized Clinical Trials. J. Am. Heart Assoc..

[245] Asghari P., Babaei A., Zamanian N., Eshtivani E.N. (2025). Berberine’s Impact on Health: Comprehensive Biological, Pharmacological, and Nutritional Perspectives. Metab. Open.

[246] Woldeselassie M., Tamene A. (2024). Therapeutic Controversies over Use of Antioxidant Supplements During Cancer Treatment: A Scoping Review. Front. Nutr..

[247] Turck D., Bohn T., Cámara M., Castenmiller J., De Henauw S., Hirsch-Ernst K.I., Jos Á., Mangelsdorf I., McNulty B., EFSA Panel on Nutrition, Novel Foods and Food Allergens (NDA) (2025). Scientific Opinion on Additional Scientific Data Related to the Safety of Monacolins from Red Yeast Rice Submitted Pursuant to Article 8(4) of Regulation (EC) No 1925/2006. EFSA J..

[248] Oketch-Rabah H.A., Roe A.L., Rider C.V., Bonkovsky H.L., Giancaspro G.I., Navarro V., Paine M.F., Betz J.M., Marles R.J., Casper S. (2020). United States Pharmacopeia (USP) Comprehensive Review of the Hepatotoxicity of Green Tea Extracts. Toxicol. Rep..

[249] Theodosis-Nobelos P., Rekka E.A. (2024). The Antioxidant Potential of Vitamins and Their Implication in Metabolic Abnormalities. Nutrients.

[250] Chang J.P., Tseng P.T., Zeng B.S., Chang C.H., Su H., Chou P.H., Su K.P. (2023). Safety of Supplementation of Omega-3 Polyunsaturated Fatty Acids: A Systematic Review and Meta-Analysis of Randomized Controlled Trials. Adv. Nutr..

[251] Ma C., Monagas M., Bronstein L., Cadwallader A., Goldman V. (2025). Dietary Supplement Adulteration: Laboratory Approaches to Risk Mitigation. J. Nat. Prod..

[252] Jasińska-Balwierz A., Krypel P., Świsłowski P., Rajfur M., Balwierz R., Ochędzan-Siodłak W. (2025). Heavy Metal Contamination in Adaptogenic Herbal Dietary Supplements: Experimental, Assessment and Regulatory Safety Perspectives. Biology.

[253] Wongtaweepkij K., Srinonghang S., Youngpattana W., Summa K., Papenkort S., Cox A.R., Jarernsiripornkul N. (2025). Public Self-Reported Adverse Experience and Knowledge About Use of Herbal and Dietary Supplements. BMC Complement. Med. Ther..

[254] Costa R., Ferreira C., Alves A., Nunes S., Reis F., Malva J., Viana S. (2025). Lipid-Lowering Statins and Polyphenol-Based Supplementation: A Scoping Review on Drug–Food Interaction Potential. Front. Pharmacol..

[255] Stępień K.A., Kalicka A., Giebułtowicz J. (2024). Screening the Quality of Legal and Illegal Dietary Supplements by LC-MS/MS. Food Addit. Contam. Part B.

[256] Shaleha R.R., Yuliana A., Amin S., Levita J., Sumiwi S.A. (2025). Monascus purpureus Red Yeast Rice: A Review of the In Vitro and In Vivo Pharmacological Activities, Studies in Humans, and Case Reports. Clin. Pharmacol. Adv. Appl..

[257] Zawistowska-Rojek A., Rybak J., Smoleń P., Kociszewska A., Rudnicki-Velasquez P., Węgrzyńska K., Zaręba T., Tyski S., Baraniak A. (2026). Evaluating the Quality of Selected Commercial Probiotic Products, Both Dietary Supplements and Foods for Special Medical Purposes. Foods.

[258] Manasa P.S.L., Kamble A.D., Chilakamarthi U. (2023). Various Extraction Techniques of Curcumin: A Comprehensive Review. ACS Omega.

[259] Subramanian P. (2021). Lipid-Based Nanocarrier System for the Effective Delivery of Nutraceuticals. Molecules.

[260] Wang H., Chen Y., Wang L., Liu Q., Yang S., Wang C. (2023). Advancing Herbal Medicine: Enhancing Product Quality and Safety Through Robust Quality Control Practices. Front. Pharmacol..

[261] Ekor M. (2014). The Growing Use of Herbal Medicines: Issues Relating to Adverse Reactions and Challenges in Monitoring Safety. Front. Pharmacol..

